# Sound Colless-like balance indices for multifurcating trees

**DOI:** 10.1371/journal.pone.0203401

**Published:** 2018-09-25

**Authors:** Arnau Mir, Lucía Rotger, Francesc Rosselló

**Affiliations:** 1 Dept. of Mathematics and Computer Science, University of the Balearic Islands, E-07122 Palma, Spain; 2 Dept. of Mathematics and Computing, University of La Rioja, E-26004 Logroño, Spain; 3 Balearic Islands Health Research Institute (IdISBa), E-07010 Palma, Spain; Oklahoma State University, UNITED STATES

## Abstract

The Colless index is one of the most popular and natural balance indices for bifurcating phylogenetic trees, but it makes no sense for multifurcating trees. In this paper we propose a family of *Colless-like* balance indices $C_{D,f}$ that generalize the Colless index to multifurcating phylogenetic trees. Each $C_{D,f}$ is determined by the choice of a dissimilarity *D* and a weight function $f:N\to R_{\geq 0}$. A balance index is *sound* when the most balanced phylogenetic trees according to it are exactly the fully symmetric ones. Unfortunately, not every Colless-like balance index is sound in this sense. We prove then that taking *f*(*n*) = ln(*n* + *e*) or *f*(*n*) = *e*^*n*^ as weight functions, the resulting index $C_{D,f}$ is sound for every dissimilarity *D*. Next, for each one of these two functions *f* and for three popular dissimilarities *D* (the variance, the standard deviation, and the mean deviation from the median), we find the most unbalanced phylogenetic trees according to $C_{D,f}$ with any given number *n* of leaves. The results show that the growth pace of the function *f* influences the notion of “balance” measured by the indices it defines. Finally, we introduce our R package “CollessLike,” which, among other functionalities, allows the computation of Colless-like indices of trees and their comparison to their distribution under Chen-Ford-Winkel’s *α*-*γ*-model for multifurcating phylogenetic trees. As an application, we show that the trees in TreeBASE do not seem to follow either the uniform model for multifurcating trees or the *α*-*γ*-model, for any values of *α* and *γ*.

## Introduction

Since the early 1970s, the shapes of phylogenetic trees have been used to test hypothesis about the evolutive forces underlying their assembly [1]. The most used topological feature of phylogenetic trees in this regard is their symmetry, which captures the symmetry of the evolutionary histories described by them. The symmetry of a tree is usually measured through its *balance* (see [2], pp. 559–560), the tendency of the children of any given node to have the same number of descendant leaves. Several *balance indices* have been proposed so far to quantify the balance of a phylogenetic tree. The two most popular ones are the *Colless index* [3], whose definition we recall below and that only works for bifurcating trees, and the *Sackin index* [4–6], which is defined as the sum of the depths of the leaves in the tree and can be used on multifurcating trees. Other balance indices for bifurcating trees introduced so far include the variance of the depths of the leaves [4, 5], the sum of the reciprocals of the orders of the rooted subtrees [6], and the number of cherries [7]. As for balance indices for multifurcating trees, two recent additions are the total cophenetic index [8] and the quartet index [9]; for more proposals, see the section “Measures of overall asymmetry” in Felsenstein’s book [2] (pp. 562–563). This abundance of balance indices is partly motivated by Shao and Sokal’s advice on using more than one such index to quantify tree balance: see [6], p. 1990.

The *Colless index* *C*(*T*) of a bifurcating phylogenetic tree *T* is defined as the sum of the balance values of its internal nodes, where by the *balance value* of an internal node we mean the absolute value of the difference between the number of descendant leaves of its pair of children. In this way, the Colless index of a bifurcating tree measures the average balance value of its internal nodes, and therefore it quantifies in a very intuitive way its balance. In particular, *C*(*T*) = 0 if, and only if, *T* is a fully symmetric bifurcating tree with 2^*m*^ leaves, for some *m*.

**Fig 1:**
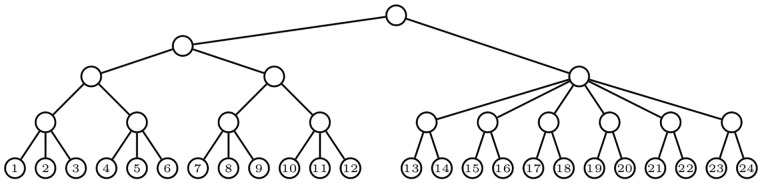
Each node in this asymmetric tree has all its children with the same number of descendant leaves as well as with the same number of descendant nodes.

Unfortunately, the Colless index can only be used as defined on bifurcating trees. A natural generalization to multifurcating trees would be to define the balance value of a node as some measure of the dissimilarity of the numbers of descendant leaves of its children, like for instance their standard deviation, and then to add up all these balance values. But this definition has a drawback: this sum can be 0 on non-symmetric multifurcating trees, and hence the resulting index need not capture the symmetry of a tree in a sound way. For an example of this misbehavior, consider the tree depicted in Fig 1: each one of its nodes has all its children with the same number of descendant leaves and therefore the balance value of each node is 0 independently on the dissimilarity used to define it, but the tree is not symmetric. Replacing the number of descendant leaves by the number of descendant nodes, which in a bifurcating tree is simply twice the number of descendant leaves minus 1, does not overcome this drawback: again, all children of each node in the tree depicted in Fig 1 have the same number of descendant nodes.

In this paper we solve this problem by taking a suitable function $f:N\to R_{\geq 0}$ and then replacing in this schema the number of descendant leaves or the number of descendant nodes of a node by the *f*-*size* of the subtree rooted at the node, defined as the sum of the images under *f* of the out-degrees of the nodes in the subtree. Then, we define the *balance value* (relative to such a function *f* and a dissimilarity *D*) of an internal node in a phylogenetic tree as the value of *D* applied to the *f*-sizes of the subtrees rooted at the children of the node. Finally, we define the *Colless-like index* $C_{D,f}$ of a phylogenetic tree as the sum of the balance values relative to *f* and *D* of its internal nodes.

The advantage of such a general definition is that there exist functions *f* such that, for every dissimilarity *D*, the resulting index $C_{D,f}$ satisfies that $C_{D,f}\left(T\right)=0$ if, and only if, *T* is *fully symmetric*, in the sense that, for every internal node *v*, the subtrees rooted at the children of *v* have all the same shape. Two such functions turn out to be *f*(*n*) = ln(*n* + *e*) and *f*(*n*) = *e*^*n*^.

The different growth pace of these two functions make them quantify the trees’ balance in different ways. We show it by finding the trees that are maximally unbalanced according to $C_{D,f}$, that is, the trees with largest $C_{D,f}$ value, when *f* is one of these two functions and *D* is the variance, the standard deviation, and the mean deviation from the median. We show that the choice of the dissimilarity *D* does not cause any major difference in the maximally unbalanced trees relative to $C_{D,f}$ for a fixed *f*, but that changing the function *f* implies completely different maximally unbalanced trees.

We have written an R package called *CollessLike*, available at the CRAN, that, among other functionalities, computes Colless-like indices and simulates their distribution under the *α*-*γ*-model for multifurcating trees [10]. We have used the functions in this package to perform two experiments on the TreeBASE phylogenetic database [11]. First, we have compared the behavior of the Colless-like index obtained by taking *f*(*n*) = ln(*n* + *e*) and as dissimilarity *D* the mean deviation from the median, MDM, with two other balance indices for multifurcating trees: the Sackin index and the total cophenetic index. Next, we have used this Colless-like index to contrast the goodness of fit of the trees in TreeBASE to the uniform distribution for multifurcating trees and to the *α*-*γ*-model.

## Materials

### Notations and conventions

Throughout this paper, by a *tree* we always mean a rooted, finite tree without out-degree 1 nodes. As usual, we consider such a tree to be a directed graph, with its arcs pointing away from the root. Given a tree *T*, we shall denote its sets of nodes, of *internal* (that is, non-leaf) nodes, and of arcs by *V*(*T*), *V*_*int*_(*T*), and *E*(*T*), respectively, and the out-degree of a node *v* ∈ *V*(*T*) by deg(*v*). A tree *T* is *bifurcating* when deg(*v*) = 2 for every *v* ∈ *V*_*int*_(*T*). Whenever we want to emphasize the fact that a tree need not be bifurcating, we shall call it *multifurcating*. The *depth* of a node in a tree *T* is the length (i.e., the number of arcs) of the directed path from the root to it, and the *depth* of *T* is the largest depth of any of its leaves. We shall always make the abuse of language of saying that two isomorphic trees are equal, and hence we shall always identify any tree with its isomorphism class. We shall denote by $T_{n}^{*}$ the set of (isomorphism classes of) trees with *n* leaves, and by $T^{*}$ the union $\cup_{n\geq 1}T_{n}^{*}$.

A *phylogenetic tree* on a (non-empty, finite) set *X* of *labels* is a tree with its leaves bijectively labelled in the set *X*. We shall always identify every leaf in a phylogenetic tree *T* on *X* with its label, and in particular we shall denote its set of leaves by *X*. Two phylogenetic trees *T*_1_, *T*_2_ on *X* are *isomorphic* when there exists an isomorphism of directed graphs between them that preserves the labelling of the leaves. We shall also make always the abuse of language of considering two isomorphic phylogenetic trees as equal. Given a set of labels *X*, we shall denote by $T_{X}$ the set of (isomorphism classes of) phylogenetic trees on *X*, and we shall denote by $T_{n}$, for every *n* ≥ 1, the set $T_{\left\{1,2,\ldots ,n\right\}}$. Notice that if |*X*| = *n*, then any bijection *X* ↔ {1, 2, …, *n*} induces a bijection $T_{X}↔T_{n}$. Moreover, if |*X*| = *n*, there is a forgetful mapping $\pi_{X}:T_{X}\to T_{n}^{*}$ that sends every phylogenetic tree to the corresponding unlabeled tree, which we shall call its *shape*.

No closed formula is known for the numbers $\left|T_{n}^{*}\right|$ or $\left|T_{n}\right|$. Felsenstein gives in Chapter 3 in [2] an easy recurrence to compute $\left|T_{n}\right|$ and describes how to obtain such a recurrence for $\left|T_{n}^{*}\right|$; an explicit algorithm to compute the latter is provided in [12]. These numbers $(|T_{n}|{)}_{n}$ and $(|T_{n}^{*}|{)}_{n}$ form sequences A000311 and A000669, respectively, in Sloane’s *On*-*Line Encyclopedia of Integer Sequences* [13], where more information about them can be found.

**Fig 2:**
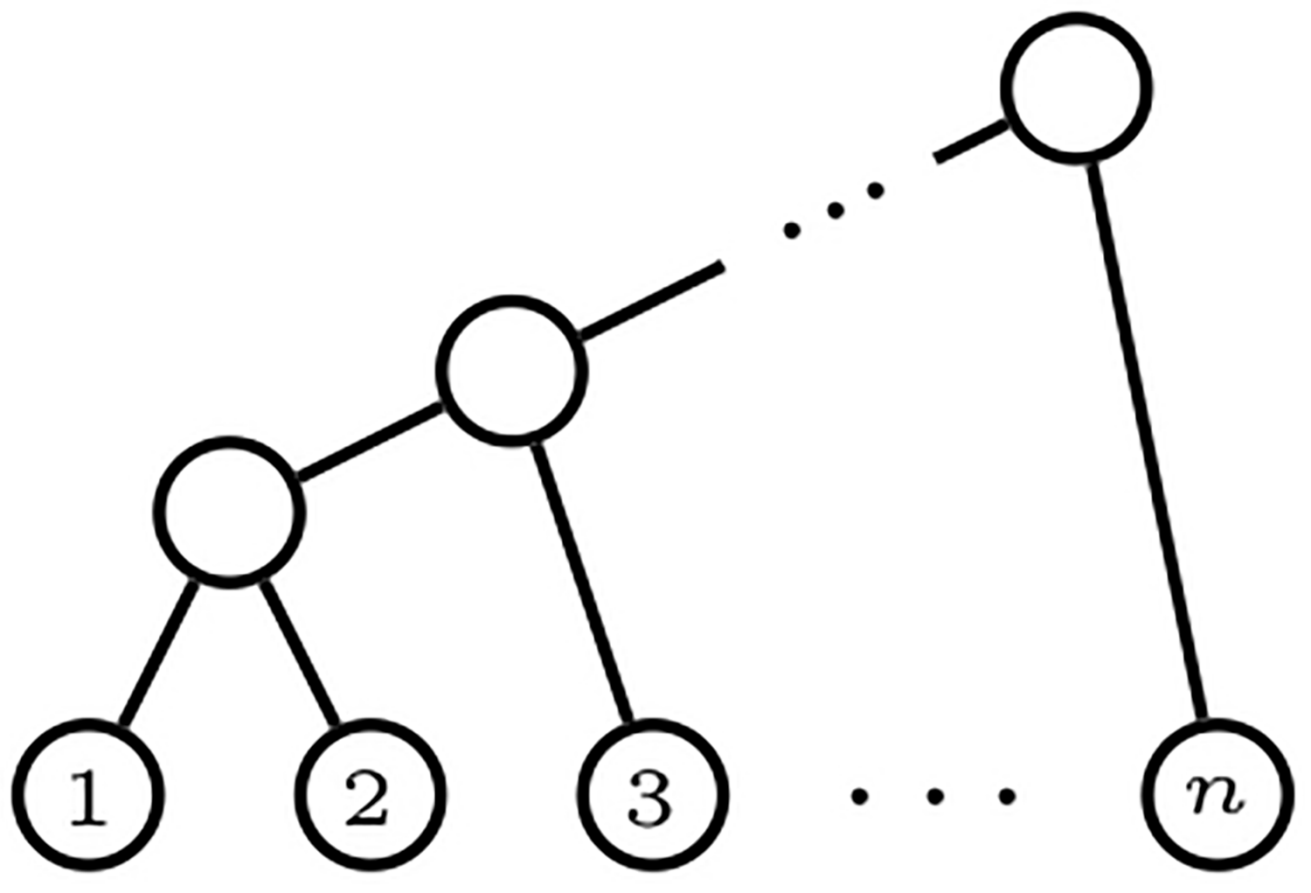
A comb *K*_*n*_ with *n* leaves.

**Fig 3:**
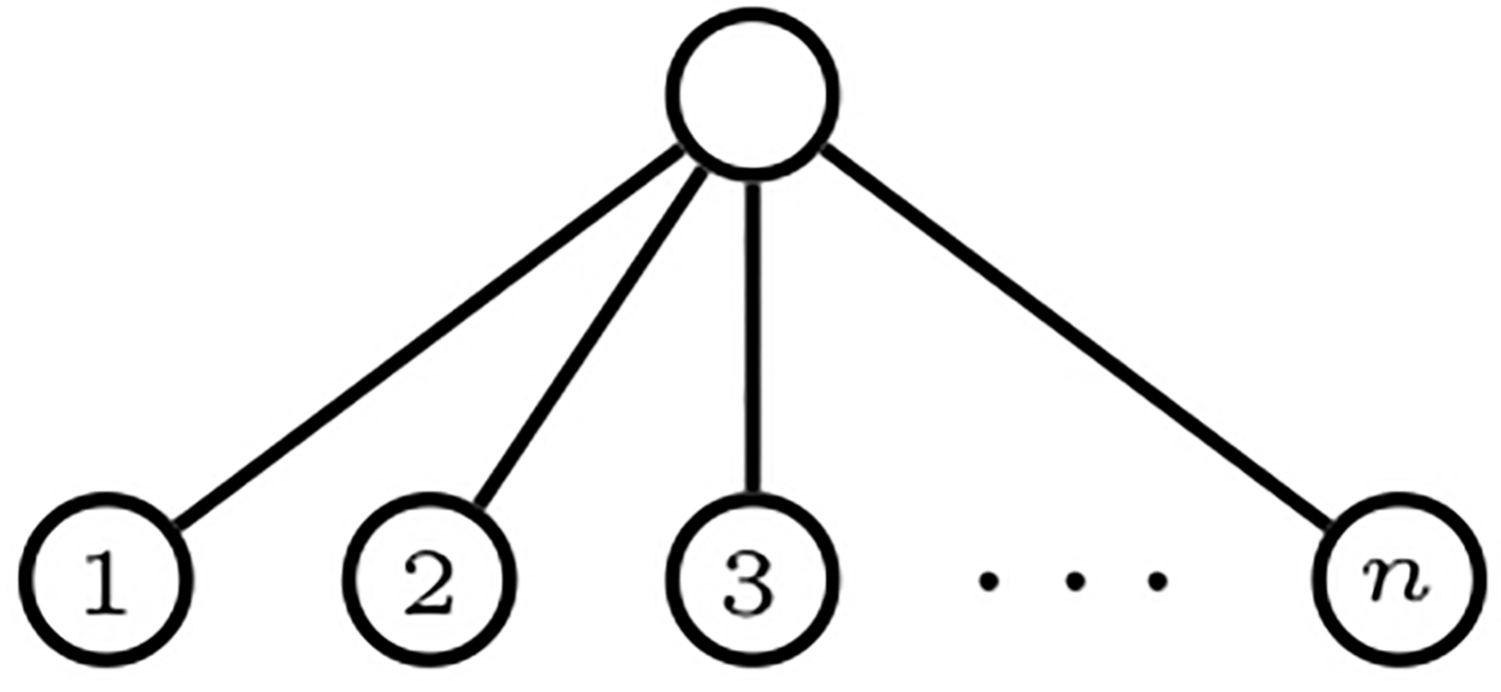
A star *FS*_*n*_ with *n* leaves.

A *comb* is a bifurcating phylogenetic tree with all its internal nodes having a leaf child: see Fig 2. We shall generically denote every comb in $T_{n}$, as well as their shape in $T_{n}^{*}$, by *K*_*n*_. A *star* is a phylogenetic tree of depth 1: see Fig 3. For consistency with later notations, we shall denote the star in $T_{n}$, and its shape in $T_{n}^{*}$, by *FS*_*n*_.

**Fig 4:**
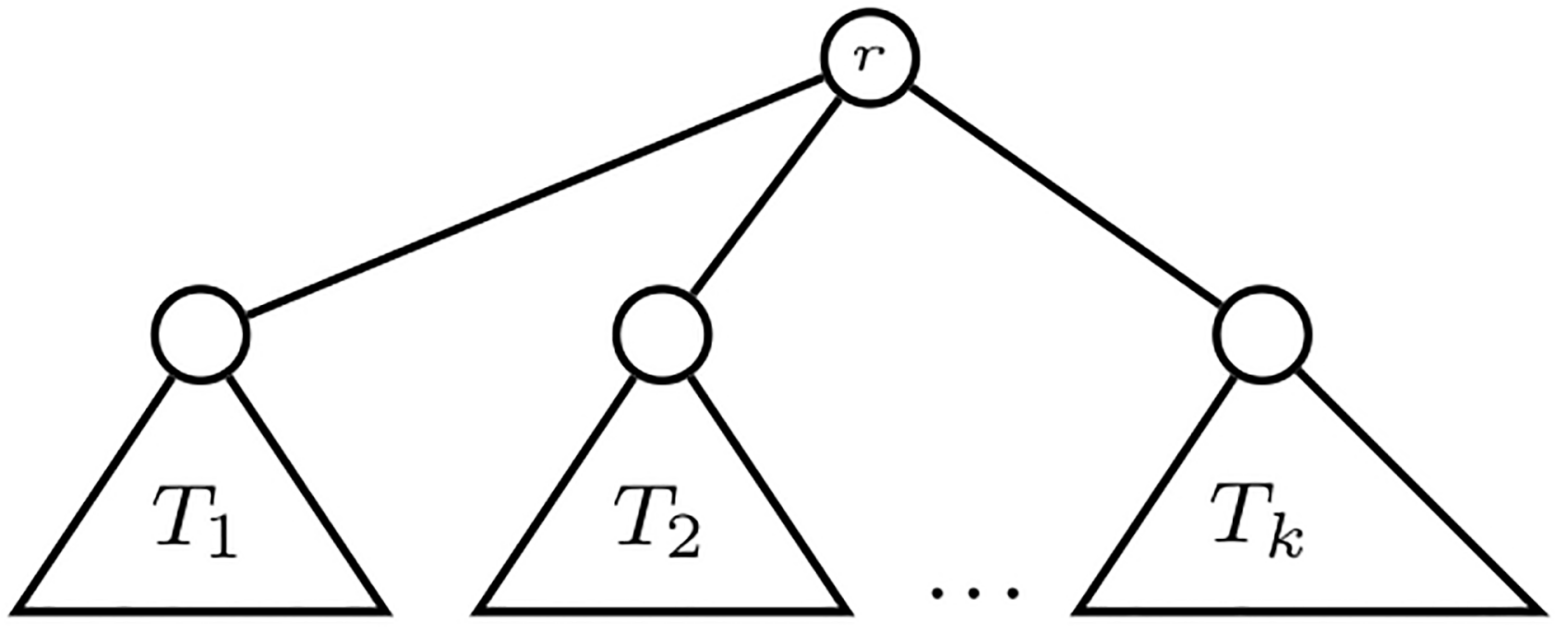
The (phylogenetic) tree *T*_1_ ⋆ ⋯ ⋆*T*_*k*_.

Let *T*_1_, …, *T*_*k*_ be phylogenetic trees on pairwise disjoint sets of labels *X*_1_, …, *X*_*k*_, respectively. The phylogenetic tree *T*_1_⋆ ⋯ ⋆*T*_*k*_ on *X*_1_ ∪ ⋯ ∪ *X*_*k*_ is obtained by adding to the disjoint union of *T*_1_, …, *T*_*k*_ a new node *r* and new arcs from *r* to the root of each *T*_*i*_. In this way, the trees *T*_1_, …, *T*_*k*_ become the subtrees of *T*_1_⋆ ⋯ ⋆*T*_*k*_ rooted at the children of its root *r*; cf. Fig 4. A similar construction produces a tree *T*_1_⋆ ⋯ ⋆*T*_*k*_ from a set of (unlabeled) trees *T*_1_, …, *T*_*k*_.

Given a node *v* in a tree *T*, we shall denote by *T*_*v*_ the subtree of *T* rooted at *v* and by *κ*_*v*_ its number of descendant leaves, that is, the number of leaves of *T*_*v*_. An internal node *v* of a tree *T* is *symmetric* when, if *v*_1_, …, *v*_*k*_ are its children, the trees $T_{v_{1}},\ldots ,T_{v_{k}}$ are isomorphic. A tree *T* is *fully symmetric* when all its internal nodes are symmetric, and a phylogenetic tree is *fully symmetric* when its shape is so.

**Fig 5:**
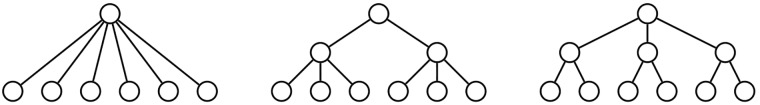
Three fully symmetric trees with 6 leaves: From left to right, *FS*_6_, *FS*_2,3_ and *FS*_3,2_.

Given a number *n* of leaves, there may exist several fully symmetric trees with *n* leaves. For instance, there are three fully symmetric trees with 6 leaves, depicted in Fig 5. In fact, every fully symmetric tree with *n* leaves is characterized by an ordered factorization *n*_1_ … *n*_*k*_ of *n*, with *n*_1_, …, *n*_*k*_ ≥ 2. More specifically, for every *k* ≥ 1 and $\left(n_{1},\ldots ,n_{k}\right)\in N^{k}$ with *n*_1_, …, *n*_*k*_ ≥ 2, let $FS_{n_{1},\ldots ,n_{k}}$ be the tree defined, up to isomorphism, recursively as follows:

- ${FS}_{n_{1}}$ is the star with *n*_1_ leaves.
- If *k* ≥ 2, $FS_{n_{1},\ldots ,n_{k}}$ is a tree whose root has *n*_1_ children, and the subtrees at each one of these children are (isomorphic to) $FS_{n_{2},\ldots ,n_{k}}$.

Every $FS_{n_{1},\ldots ,n_{k}}$ is fully symmetric, and every fully symmetric tree is isomorphic to some $FS_{n_{1},\ldots ,n_{k}}$. Therefore, for every *n*, the number of fully symmetric trees with *n* leaves is equal to the number *H*(*n*) of ordered factorizations of *n* (sequence A074206 in Sloane’s *On*-*Line Encyclopedia of Integer Sequences* [13]).

### The Colless index

The *Colless index C*(*T*) of a bifurcating tree *T* with *n* leaves is defined as follows [3]: if, for every *v* ∈ *V*_*int*_(*T*), we denote by *v*_1_ and *v*_2_ its two children and by $\kappa_{v_{1}}$ and $\kappa_{v_{2}}$ their respective numbers of descendant leaves, then

$$C\left(T\right)=\sum_{v\in V_{int}\left(T\right)}\left|\kappa_{v_{1}}-\kappa_{v_{2}}\right|.$$

The Colless index of a phylogenetic tree is simply defined as the Colless index of its shape.

It is well-known that the maximum Colless index on the set of bifurcating trees with *n* leaves is reached at the comb *K*_*n*_, and it is

$$C\left(K_{n}\right)=\left(\begin{array}{c} n-1\\ 2 \end{array}\right)$$

(see, for instance, [14]). In fact, this maximum is only reached at the comb. Since we have not been able to find an explicit reference for this last result in the literature and we shall make use of it later, we provide a proof here.

**Lemma 1**. *For every bifurcating tree T with n leaves*, *if T* ≠ *K*_*n*_, *then C*(*T*) < *C*(*K*_*n*_).

**Fig 6:**
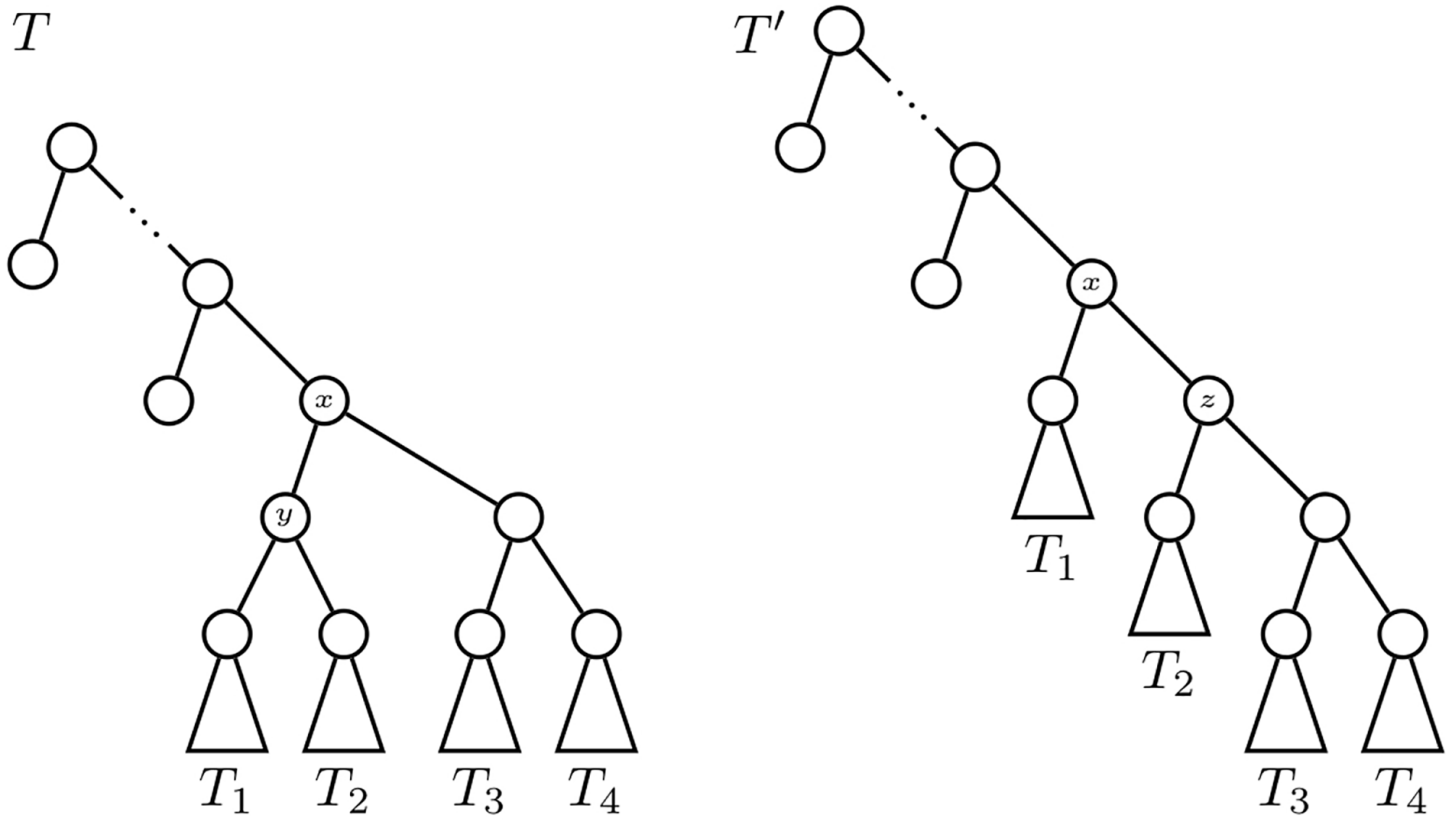
The trees *T* and *T*′ in the proof of Lemma 1.

**Proof**. Let *T* a bifurcating tree with *n* leaves different from the comb *K*_*n*_. Let *x* be an internal node of smallest depth in it without any leaf child, and let *T*_1_ ⋆ *T*_2_ and *T*_3_ ⋆ *T*_4_ be the subtrees rooted at its children (see Fig 6); for every *i* = 1, 2, 3, 4, let *t*_*i*_ be the number of leaves of *T*_*i*_. Assume, without any loss of generality, that *t*_1_ ≤ *t*_2_ and *t*_1_ + *t*_2_ ≤ *t*_3_ + *t*_4_. Let then *T*′ be the tree obtained by pruning *T*_2_ from *T* and regrafting it to the other arc starting in *x* (see again Fig 6).

Then *C*(*T*′) > *C*(*T*). Indeed, the only nodes whose children change their numbers of descendant leaves from *T* to *T*′ are (cf. Fig 6): the node *x*; the parent *y* of the roots of *T*_1_ and *T*_2_ in *T*, which is removed in *T*′; and the parent *z* of the root of *T*_2_ in *T*′, which does not exist in *T*. Therefore,

$$\begin{array}{c} \begin{array}{c} C\left(T^{\prime }\right)-C\left(T\right)\\ \;=|t_{3}+t_{4}-t_{2}|+[t_{3}+t_{4}+t_{2}-t_{1}\left|-\right|t_{2}-t_{1}\left|-\right|t_{3}+t_{4}-t_{2}-t_{1}|\\ \;=t_{3}+t_{4}-t_{2}+t_{3}+t_{4}+t_{2}-t_{1}-t_{2}+t_{1}-t_{3}-t_{4}+t_{2}+t_{1}\\ \;=t_{1}+t_{3}+t_{4}>0. \end{array} \end{array}$$

So, this procedure takes a bifurcating tree with *n* leaves *T* ≠ *K*_*n*_ and produces a new bifurcating tree *T*′ with the same number *n* of leaves and strictly larger Colless index. Since the number of bifurcating trees with *n* leaves is finite, the Colless index cannot increase indefinitely, which means that if we iterate this procedure, we must eventually stop at a comb *K*_*n*_. And since the Colless index strictly increases at each iteration, we conclude that if *T* ≠ *K*_*n*_, then *C*(*T*) < *C*(*K*_*n*_).

## Methods

### Colless-like indices

Let $f:N\to R_{\geq 0}$ be a function that sends each natural number to a positive real number. The *f*-*size* of a tree $T\in T^{*}$ is defined as

$$\delta_{f}\left(T\right)=\sum_{v\in V(T)}f\left(\text{deg}\left(v\right)\right).$$

If $T\in T_{X}$, for some set of labels *X*, then *δ*_*f*_(*T*) is defined as *δ*_*f*_(*π*_*X*_(*T*)).

Therefore, *δ*_*f*_(*T*) is the sum of the degrees of all nodes in *T*, with these degrees weighted by means of the function *f*. Examples of *f*-sizes include:

- The *number of leaves*, *κ*, which is obtained by taking *f*(0) = 1 and *f*(*n*) = 0 if *n* > 0.
- The *order* (the number of nodes), *τ*, which corresponds to *f*(*n*) = 1 for every n∈N.
- The usual *size* (the number of arcs), *θ*, which corresponds to *f*(*n*) = *n* for every n∈N.

Notice that *δ*_*f*_ satisfies the following recursion:

$$\delta_{f}\left(T_{1}⋆\cdots ⋆T_{k}\right)=\delta_{f}\left(T_{1}\right)+\cdots +\delta_{f}\left(T_{k}\right)+f\left(k\right).$$

Table A in S2 File gives the abstract values of *δ*_*f*_(*T*) for every $T\in T_{n}^{*}$ with *n* = 2, 3, 4, 5.

**Example 2**. *If T is a bifurcating tree with n leaves*, *and hence with n* − 1 *internal nodes*, *all of them of out-degree 2*, *then*

$$\delta_{f}\left(T\right)=\left(f\left(0\right)+f\left(2\right)\right)n-f\left(2\right).$$

**Example 3**. *For every fully symmetric tree* $FS_{n_{1},\ldots ,n_{k}}$,

$$\delta_{f}\left(FS_{n_{1},\ldots ,n_{k}}\right)=n_{1}\cdots n_{k}\cdot f\left(0\right)+n_{1}\cdots n_{k-1}\cdot f\left(n_{k}\right)+\cdots +n_{1}\cdot f\left(n_{2}\right)+f\left(n_{1}\right).$$

Now let

$$R^{+}=\underset{k\geq 1}{\cup }R^{k}=\left\{\left(x_{1},\ldots ,x_{k}\right)|k\geq 1,x_{1},\ldots ,x_{k}\in R\right\}$$

be the set of all non-empty finite-length sequences of real numbers. A *dissimilarity* on $R^{+}$ is any mapping $D:R^{+}\to R_{\geq 0}$ satisfying the following conditions: for every $\left(x_{1},\ldots ,x_{k}\right)\in R^{+}$,

- *D*(*x*_1_, …, *x*_*k*_) = *D*(*x*_*σ*(1)_, …, *x*_*σ*(*k*)_), for every permutation *σ* of {1, …, *k*};
- *D*(*x*_1_, …, *x*_*k*_) = 0 if, and only if, *x*_1_ = ⋯ = *x*_*k*_.

The dissimilarities that we shall explicitly use in this paper are the *mean deviation from the median*,

$$\text{MDM}\left(x_{1},\ldots ,x_{k}\right)=\frac{1}{k}\sum_{i=1}^{k}|x_{i}-Median\left(x_{1},\ldots ,x_{k}\right)|,$$

the (*sample*) *variance*,

$$\text{var}\left(x_{1},\ldots ,x_{k}\right)=\frac{1}{k-1}\sum_{i=1}^{k}(x_{i}-Mean\left(x_{1},\ldots ,x_{k}\right){)}^{2},$$

and the (*sample*) *standard deviation*,

$$sd\left(x_{1},\ldots ,x_{k}\right)=+\sqrt{\text{var}(x_{1},\ldots ,x_{k})}.$$

Let *D* be a dissimilarity on $R^{+}$, $f:N\to R_{\geq 0}$ a function, and *δ*_*f*_ the corresponding *f*-size, and let $T\in T^{*}$. For every internal node *v* in *T*, with children *v*_1_, …, *v*_*k*_, the (*D*, *f*)-*balance value* of *v* is

$${bal}_{D,f}\left(v\right)=D\left(\delta_{f}\left(T_{v_{1}}\right),\ldots ,\delta_{f}\left(T_{v_{k}}\right)\right).$$

So, *bal*_*D*,*f*_(*v*) measures, through *D*, the spread of the *f*-sizes of the subtrees rooted at the children of *v*. In particular, *bal*_*D*, *f*_(*v*) = 0 if, and only if, $\delta_{f}\left(T_{v_{1}}\right)=\cdots =\delta_{f}\left(T_{v_{k}}\right)$.

**Definition 4**. *Let D be a dissimilarity on* $R^{+}$ *and* $f:N\to R_{\geq 0}$ *a function*. *For every* $T\in T^{*}$, *its* Colless-like index relative to *D* and *f*, $C_{D,f}\left(T\right)$, *is the sum of the* (*D*, *f*)-*balance values of the internal nodes of T*:

$$C_{D,f}\left(T\right)=\sum_{v\in V_{int}\left(T\right)}{bal}_{D,f}\left(v\right).$$

*If* $T\in T_{X}$, *for some set of labels X*, *then* $C_{D,f}\left(T\right)$ *is defined as* $C_{D,f}\left(\pi_{X}\left(T\right)\right)$.

**Example 5**. *If we take* *D* = MDM *and f the constant mapping 1*, *so that δ*_*f*_ = *τ*, *the usual order of a tree*, *then*

$$\begin{array}{c} \begin{array}{cc} C_{\text{MDM},\tau }\left(T\right) & =\sum_{v\in V_{int}\left(T\right)}\text{MDM}\left(\tau_{v_{1}},\ldots ,\tau_{v_{\text{deg}(v)}}\right)\\ & =\sum_{v\in V_{int}\left(T\right)}\frac{1}{\text{deg}(v)}\sum_{i=1}^{\text{deg}(v)}\left|\tau_{v_{1}}-Median\left(\tau_{v_{1}},\ldots ,\tau_{v_{\text{deg}(v)}}\right)\right|, \end{array} \end{array}$$

*where*, *for every v* ∈ *V*_*int*_(*T*), *v*_1_, …, *v*_deg(*v*)_ *denote its children and* $\tau_{v_{1}},\ldots ,\tau_{v_{\text{deg}(v)}}$ *their numbers of descendant nodes*.

Notice that $C_{D,f}$ gets larger as the *f*-sizes of the subtrees rooted at siblings get more different, and therefore it behaves as a balance index for trees, in the same way as, for instance, the Colless index for bifurcating trees: the smaller the value of $C_{D,f}\left(T\right)$, the more balanced is *T* relative to the *f*-size *δ*_*f*_.

It is clear that $C_{D,f}$ satisfies the following recursion:

$$C_{D,f}\left(T_{1}⋆\cdots ⋆T_{k}\right)=C_{D,f}\left(T_{1}\right)+\cdots +C_{D,f}\left(T_{k}\right)+D\left(\delta_{f}\left(T_{1}\right),\ldots ,\delta_{f}\left(T_{k}\right)\right).$$

Therefore these Colless-like indices are *recursive tree shape statistics* in the sense of [15], relative to the *f*-size *δ*_*f*_. Table A in S2 File also gives the abstract values of $C_{D,f}\left(T\right)$, for *D* = MDM, var, and *sd*, and for every $T\in T_{n}^{*}$ with *n* = 2, 3, 4, 5.

The next result shows that, if we take *D* = MDM or *D* = *sd*, then any index $C_{D,f}$ restricted to only bifurcating trees defines, up to a constant factor, the usual Colless index.

**Proposition 6**. *Let T be a bifurcating tree with n leaves and* $f:N\to R_{\geq 0}$ *any function*. *Then*,

$$C_{\text{MDM},f}\left(T\right)=\frac{f(0)+f(2)}{2}\cdot C\left(T\right),\;C_{sd,f}\left(T\right)=\frac{f(0)+f(2)}{\sqrt{2}}\cdot C\left(T\right).$$

**Proof**. Notice that, for every x,y∈R, $\text{MDM}\left(x,y\right)=\frac{1}{2}\left|x-y\right|$ and $sd\left(x,y\right)=\frac{1}{\sqrt{2}}\left|x-y\right|$. We shall prove the statement for MDM; the proof for *sd* is identical, replacing the 2 in the denominator by $\sqrt{2}$. For every internal node *v* in a bifurcating tree *T*, if *v*_1_ and *v*_2_ denote its children,

$$\begin{array}{c} {bal}_{MDM,f}\left(v\right)=\frac{1}{2}\left|\delta_{f}\left(T_{v_{1}}\right)-\delta_{f}\left(T_{v_{2}}\right)\right|\\ \;\;=\frac{1}{2}\left|\left(\left(f\left(0\right)+f\left(2\right)\right)\kappa_{v_{1}}-f\left(2\right)\right)-\left(\left(f\left(0\right)+f\left(2\right)\right)\kappa_{v_{2}}-f\left(2\right)\right)\right|\\ \;\;(\text{by}\;\text{Example}\;2)\\ \;\;=\frac{f(0)+f(2)}{2}\cdot \left|\kappa_{v_{1}}-\kappa_{v_{2}}\right| \end{array}$$

and therefore

$$\begin{array}{c} \begin{array}{cc} C_{MDM,f}\left(T\right) & =\sum_{v\in V_{int}\left(T\right)}{bal}_{MDM,f}\left(v\right)=\frac{f(0)+f(2)}{2}\cdot \;\sum_{v\in V_{int}\left(T\right)}\left|\kappa_{v_{1}}-\kappa_{v_{2}}\right|\\ & =\frac{f(0)+f(2)}{2}\cdot C\left(T\right), \end{array} \end{array}$$

as we claimed.

If we define the *quadratic Colless index* of a bifurcating tree *T* as

$$C^{\left(2\right)}\left(T\right)=\sum_{v\in V_{int}\left(T\right)}{\left(\kappa_{v_{1}}-\kappa_{v_{2}}\right)}^{2}$$

(where, for every *v* ∈ *V*_*int*_(*T*), *v*_1_, *v*_2_ denote its children), then, given that $\text{var}\left(x,y\right)=\frac{1}{2}{\left(x-y\right)}^{2}$, a similar argument proves the following result.

**Proposition 7**. *Let T be a bifurcating tree with n leaves and* $f:N\to R_{\geq 0}$ *any function*. *Then*,

$$C_{\text{var},f}\left(T\right)=\frac{{\left(f\left(0\right)+f\left(2\right)\right)}^{2}}{2}\cdot C^{\left(2\right)}\left(T\right).$$

As for the cost of computing Colless-like indices, we have the following result.

**Proposition 8**. *If the cost of computing D*(*x*_1_, …, *x*_*k*_) *is in O*(*k*) *and the cost of computing each f*(*k*) *is at most in O*(*k*), *then*, *for every* $T\in T_{n}^{*}$, *the cost of computing* $C_{D,f}\left(T\right)$ *is in O*(*n*).

**Proof**. Assume that every *f*(*k*) is computed in time at most *O*(*k*). For every *k* ≥ 2, let *m*_*k*_ be the number of internal nodes in *T* of out-degree *k*. Since the sizes *δ*_*f*_(*v*) are additive, in the sense that if *v* has children *v*_1_, …, *v*_*k*_, then $\delta_{f}\left(v\right)=\sum_{i=1}^{k}\delta_{f}\left(v_{i}\right)+f\left(k\right)$, we can compute the whole vector (*δ*_*f*_(*v*))_*v*∈*V*(*T*)_ in time *O*(*n* + ∑_*k*≥2_ *m*_*k*_ ⋅ *k*) = *O*(*n*) by traversing the tree in post-order.

Assume now that *D*(*x*_1_, …, *x*_*k*_) can be computed in time *O*(*k*). Then, for every internal node *v* of out-degree *k*, ${bal}_{D,f}\left(v\right)=D\left(\delta_{f}\left(T_{v_{1}}\right),\ldots ,\delta_{f}\left(T_{v_{k}}\right)\right)$ can be computed in time *O*(*k*), by simply reading the *k* *f*-sizes of its children (which are already computed) and applying *D* to them. This shows that the whole vector (*bal*_*D*, *f*_(*v*))_*v* ∈ *V*(*T*)_ can be computed again in time *O*(∑_*k*≥2_ *m*_*k*_ ⋅ *k*) = *O*(*n*). Finally, we compute $C_{D,f}\left(T\right)$ by adding up the entries of (*bal*_*D*, *f*_(*v*))_*v*∈*V*(*T*)_, which still can be done in time *O*(*n*).

The dissimilarities mentioned previously in this subsection can be computed in a number of sums and multiplications that is linear in the length of the input vector, and the specific functions *f* that we shall consider in the next subsection, basically exponentials and logarithms, can be approximated to any desired precision in constant time by using addition and look-up tables [16].

### Sound Colless-like indices

It is clear that for every dissimilarity *D*, for every function $f:N\to R_{\geq 0}$ and for every fully symmetric tree $FS_{n_{1},\ldots ,n_{k}}$, $C_{D,f}\left(FS_{n_{1},\ldots ,n_{k}}\right)=0$ because *bal*_*D*, *f*_(*v*) = 0 for every $v\in V_{int}\left(FS_{n_{1},\ldots ,n_{k}}\right)$. We shall say that a Colless-like index $C_{D,f}$ is *sound* when the converse implication is true.

**Definition 9**. *A Colless-like index* $C_{D,f}$ *is sound when*, *for every* $T\in T^{*}$, $C_{D,f}\left(T\right)=0$ *if*, *and only if*, *T is fully symmetric*.

In other words, $C_{D,f}$ is sound when, according to it, the most balanced trees are exactly the fully symmetric trees.

The Colless index *C* and its quadratic version *C*^(2)^ are sound for *bifurcating* trees. Unfortunately, this is not always so for Colless-like indices for multifurcating trees. It is not so even for direct generalizations of *C* or *C*^(2)^. For instance, $C_{\text{MDM},\kappa }$, $C_{sd,\kappa }$ and $C_{\text{var},\kappa }$, where *κ* denotes the number of leaves, are not sound; neither are $C_{\text{MDM},\tau }$, $C_{sd,\tau }$ and $C_{\text{var},\tau }$, where *τ* denotes the number of nodes; and they are not sound even when replacing *τ* by *θ*, the usual size, which is simply *τ* − 1. For example, the tree *T* in Fig 1 is not fully symmetric, but $C_{\text{MDM},\kappa }\left(T\right)=C_{\text{var},\kappa }\left(T\right)=C_{sd,\kappa }\left(T\right)=C_{\text{MDM},\tau }\left(T\right)=C_{\text{var},\tau }\left(T\right)=C_{sd,\tau }\left(T\right)=0$.

The following lemma shows that the soundness of $C_{D,f}\left(T\right)=0$ does not depend on *D*, but only on *f*.

**Lemma 10**. $C_{D,f}$ *is sound if*, *and only if*, *δ*_*f*_(*T*_1_) ≠ *δ*_*f*_(*T*_2_) *for every pair of different fully symmetric trees T*_1_, *T*_2_.

**Proof**. For the “only if” implication: if there exist two different (i.e., non isomorphic) fully symmetric trees *T*_1_, *T*_2_ such that *δ*_*f*_(*T*_1_) = *δ*_*f*_(*T*_2_), then the tree *T* = *T*_1_ ⋆ *T*_2_ is not fully symmetric, but

$$C_{D,f}\left(T\right)=C_{D,f}\left(T_{1}\right)+C_{D,f}\left(T_{2}\right)+D\left(\delta_{f}\left(T_{1}\right),\delta_{f}\left(T_{2}\right)\right)=0.$$

Conversely, assume that, for every pair of fully symmetric trees *T*_1_, *T*_2_, if *δ*_*f*_(*T*_1_) = *δ*_*f*_(*T*_2_) then *T*_1_ = *T*_2_. We shall prove by complete induction on *n* that if *T* is a tree with *n* leaves such that $C_{D,f}\left(T\right)=0$, then *T* is fully symmetric. If *T* has only one leaf, it is clearly fully symmetric. Now, assume that *n* > 1 and hence that *T* has depth at least 1. Let *T*_1_, …, *T*_*k*_, *k* ≥ 2, be its subtrees rooted at the children of its root, so that *T* = *T*_1_⋆ ⋯ ⋆*T*_*k*_. Then,

$$0=C_{D,f}\left(T\right)=\sum_{i=1}^{k}C_{D,f}\left(T_{i}\right)+D\left(\delta_{f}\left(T_{1}\right),\ldots ,\delta_{f}\left(T_{k}\right)\right)$$

implies, on the one hand, that $C_{D,f}\left(T_{1}\right)=\cdots =C_{D,f}\left(T_{k}\right)=0$, and hence, by induction, that *T*_1_, …, *T*_*k*_ are fully symmetric, and, on the other hand, that *D*(*δ*_*f*_(*T*_1_), …, *δ*_*f*_(*T*_*k*_)) = 0, and hence that *δ*_*f*_(*T*_1_) = ⋯ = *δ*_*f*_(*T*_*k*_), which, by assumption, implies that *T*_1_ = ⋯ = *T*_*k*_: in summary, *T* is fully symmetric.

The following problem now arises:

**Problem**. *To find functions* $f:N\to R_{\geq 0}$ *such that* $C_{D,f}$ *is sound*.

Unfortunately, many natural functions *f* do not define sound Colless-like indices, as the following examples show.

**Example 11**. *If f*(*n*) = *an*^2^ + *bn* + *c*, *for any a*, *b*, *c*, *then* $C_{D,f}$ *is not sound*, *because*, *for example*, *δ*_*f*_(*FS*_2,2,2,7_) = *δ*_*f*_(*FS*_14,4_) = 420*a* + 70*b* + 71*c*.

**Example 12**. *If f*(*n*) = *n*^*d*^, *for any d* ≥ 0, *then* $C_{D,f}$ *is not sound*. *Indeed*, *for every d* ≥ 3 (*the case when d* ≤ 2 *is a particular case of the last example*), *take*

- *k* = 2^*d*^ + 1 *and l* = 2;
- $n_{i}=2^{{\left(d-1\right)}^{i}d^{k-i-1}}$ *for i* = 1, …, *k* − 1;
- *n*_*k*_ = 2;
- $m_{1}=2^{(d-1)d^{k-2}+1}$;
- $m_{2}=2^{({\left(d-1\right)}^{2}(d^{k-2}-{\left(d-1\right)}^{k-2})+d-1)/d}$; *notice that this exponent is an integer number*, *because k is odd and therefore d divides* (*d* − 1)^*k*^ + 1.

*Then*

$$n_{1}\ldots n_{i-1}\cdot n_{i}^{d}=n_{1}^{d}$$

*and hence*, *on the one hand*,

$$\begin{array}{c} \begin{array}{cc} n_{1}^{d}+\cdots +n_{1}\cdots n_{k-2}\cdot n_{k-1}^{d} & =\left(k-1\right)n_{1}^{d}=2^{d}\cdot 2^{\left(d-1\right)d^{k-1}}\\ & =(2^{1+\left(d-1\right)d^{k-2}}{)}^{d}=m_{1}^{d}, \end{array} \end{array}$$

*and*, *on the other hand*,

$$\begin{array}{c} \begin{array}{cc} n_{1}\cdots n_{k-1}\cdot n_{k}^{d} & =n_{1}^{\frac{\left(1-{\left(\frac{d-1}{d}\right)}^{k-1}\right)}{\left(1-\frac{d-1}{d}\right)}}\;\cdot n_{k}^{d}=n_{1}^{\frac{d^{k-1}-{\left(d-1\right)}^{k-1}}{d^{k-2}}}n_{k}^{d}\\ & =2^{\left(d-1\right)(d^{k-1}-{\left(d-1\right)}^{k-1})+d}=m_{1}m_{2}^{d}. \end{array} \end{array}$$

*Therefore*, $\delta_{n^{d}}\left(FS_{n_{1},\ldots ,n_{k}}\right)=\delta_{n^{d}}\left(FS_{m_{1},m_{2}}\right)$.

*Of course*, *for any given d there may exist* “*smaller*” *counterexamples*: *for instance*, $\delta_{n^{3}}(FS_{2,10,4})=\delta_{n^{3}}(FS_{6,8})=3288$ *and* $\delta_{n^{4}}(FS_{2,6,2,3})=\delta_{n^{4}}(FS_{8,3})=4744$.

**Example 13**. *If f*(*n*) = log_*a*_(*n*) (*for some a* > 1) *when n* > 0, *and f*(0) = 0, *then* $C_{D,f}$ *is not sound*: *for instance*, *δ*_*f*_(*FS*_2,2_) = *δ*_*f*_(*FS*_8_) = log_*a*_(8). *In a similar way*, *if f*(*n*) = log_*a*_(*n* + 1) (*for some a* > 1), *then* $C_{D,f}$ *is not sound*, *either*: *for instance*, *δ*_*f*_(*FS*_2,3,3_) = *δ*_*f*_(*FS*_5,7_) = log_*a*_(196608).

On the positive side, we shall show now two functions that define sound indices. The following lemmas will be useful to prove it.

**Lemma 14**. *For every k*, *l* ≥ 1 and *n*_1_, *n*_2_, …, *n*_*k*_, *m*_1_, *m*_2_, …, *m*_*l*_ ≥ 2, *if* $\delta_{f}\left(FS_{n_{1},n_{2},\ldots ,n_{k}}\right)=\delta_{f}\left(FS_{m_{1},m_{2},\ldots ,m_{l}}\right)$, *n*_1_ ⋅ *n*_2_⋯*n*_*k*_ = *m*_1_ ⋅ *m*_2_⋯*m*_*l*_, *and n*_*k*_ = *m*_*l*_, *then* $\delta_{f}\left(FS_{n_{1},\ldots ,n_{k-1}}\right)=\delta_{f}\left(FS_{m_{1},\ldots ,m_{l-1}}\right)$.

**Proof**. *If n*_1_⋯*n*_*k*_ = *m*_1_⋯*m*_*l*_ and *n*_*k*_ = *m*_*l*_, then *n*_1_⋯*n*_*k*−1_ = *m*_1_⋯*m*_*l*−1_. If, moreover, $\delta_{f}\left(FS_{n_{1},n_{2},\ldots ,n_{k}}\right)=\delta_{f}\left(FS_{m_{1},m_{2},\ldots ,m_{l}}\right)$, that is,

$$\begin{array}{c} \begin{array}{c} n_{1}\cdots n_{k}f\left(0\right)+n_{1}\cdots n_{k-1}f\left(n_{k}\right)+n_{1}\cdots n_{k-2}f\left(n_{k-1}\right)+\cdots +f\left(n_{1}\right)\\ \;=m_{1}\cdots m_{l}f\left(0\right)\;+m_{1}\cdots m_{l-1}f\left(m_{l}\right)\;+m_{1}\cdots m_{l-2}f\left(m_{l-1}\right)\;+\cdots +\;f\left(m_{1}\right), \end{array} \end{array}$$

then

$$\begin{array}{c} \begin{array}{c} n_{1}\cdots n_{k-2}f\left(n_{k-1}\right)+\cdots +n_{1}f\left(n_{2}\right)+f\left(n_{1}\right)\\ \;\;=m_{1}\cdots m_{l-2}f\left(m_{l-1}\right)+\cdots +m_{1}f\left(m_{2}\right)+f\left(m_{1}\right) \end{array} \end{array}$$

and hence

$$\begin{array}{c} \begin{array}{c} \delta_{f}\left(FS_{n_{1},n_{2},\ldots ,n_{k-1}}\right)\\ \;=n_{1}\cdots n_{k-1}f\left(0\right)+n_{1}\cdots n_{k-2}f\left(n_{k-1}\right)+\cdots +n_{1}f\left(n_{2}\right)+f\left(n_{1}\right)\\ \;=m_{1}\cdots m_{l-1}f\left(0\right)+m_{1}\cdots m_{l-2}f\left(m_{l-1}\right)+\cdots +m_{1}f\left(m_{2}\right)+f\left(m_{1}\right)\\ \;=\delta_{f}\left(FS_{m_{1},\ldots ,m_{l-1}}\right) \end{array} \end{array}$$

as claimed.

**Lemma 15**. *If n*_1_, …, *n*_*k*_ ≥ 2, *then*

$$1+n_{1}+n_{1}n_{2}+\cdots +n_{1}\cdots n_{k-1}<n_{1}\cdots n_{k}.$$

**Proof**. By induction on *k*. If *k* = 1, the statement says that 1 < *n*_1_, which is true by assumption. Assume now that the statement is true for any *n*_1_, …, *n*_*k*_ ≥ 2, and let *n*_*k*+1_ ≥ 2. Then,

$$\begin{array}{c} \begin{array}{c} 1+n_{1}+n_{1}n_{2}+\cdots +n_{1}\cdots n_{k-1}+n_{1}\cdots n_{k}<n_{1}\cdots n_{k}+n_{1}\cdots n_{k}\\ =2n_{1}\cdots n_{k}\leq n_{1}\cdots n_{k}\cdot n_{k+1}. \end{array} \end{array}$$

**Proposition 16**. *If f*(*n*) = *e*^*n*^, *then* $C_{D,f}$ *is sound*.

**Proof**. Assume that there exist two non-isomorphic fully symmetric trees $FS_{n_{1},\ldots ,n_{k}}$ and $FS_{m_{1},\ldots ,m_{l}}$ such that

$$\delta_{e^{n}}\left(FS_{n_{1},\ldots ,n_{k}}\right)=\delta_{e^{n}}\left(FS_{m_{1},\ldots ,m_{l}}\right),$$

that is, such that

$$\begin{array}{c} \begin{array}{c} n_{1}\cdots n_{k}+n_{1}\cdots n_{k-1}e^{n_{k}}+\cdots +n_{1}e^{n_{2}}+e^{n_{1}}\\ \;=m_{1}\cdots m_{l}+m_{1}\cdots m_{l-1}e^{m_{l}}+\cdots +m_{1}e^{m_{2}}+e^{m_{1}}. \end{array} \end{array}$$

Assume that *l* is the smallest depth of a fully symmetric tree with *e*^*n*^-size equal to the *e*^*n*^-size of another fully symmetric tree non-isomorphic to it.

Since *e* is transcendental, the equality above implies the equality of polynomials in Z[x]

$$\begin{array}{c} \begin{array}{c} n_{1}\cdots n_{k}+n_{1}\cdots n_{k-1}x^{n_{k}}+\cdots +n_{1}x^{n_{2}}+x^{n_{1}}\\ \;\;=m_{1}\cdots m_{l}+m_{1}\cdots m_{l-1}x^{m_{l}}+\cdots +m_{1}x^{m_{2}}+x^{m_{1}}. \end{array} \end{array}$$

If *l* = 1, the right-hand side polynomial is simply $m_{1}+x^{m_{1}}$ and then the equality of polynomials implies that *k* = 1 and *n*_1_ = *m*_1_, which contradicts the assumption that $FS_{n_{1},\ldots ,n_{k}}\neq FS_{m_{1},\ldots ,m_{l}}$. Now assume that *l* ≥ 2. This equality of polynomials implies the equality of their independent terms: *n*_1_ ⋯ *n*_*k*_ = *m*_1_⋯*m*_*l*_. On the other hand, the non-zeroth power of *x* with the largest coefficient in the left-hand side polynomial is $x^{n_{k}}$ (because all coefficients are non-negative, and, by Lemma 15, *n*_1_ ⋯ *n*_*k*−1_ alone is larger than the sum *n*_1_⋯*n*_*k*−2_ + ⋯ + *n*_1_ + 1 of all other coefficients of non-zeroth powers of *x*) and, by the same reason, the non-zeroth power of *x* with the largest coefficient in the right-hand side polynomial is $x^{m_{l}}$. The equality of polynomials implies then that *n*_*k*_ = *m*_*l*_ and hence, by Lemma 14, that $\delta_{e^{n}}\left(FS_{n_{1},\ldots ,n_{k-1}}\right)=\delta_{e^{n}}\left(FS_{m_{1},\ldots ,m_{l-1}}\right)$, against the assumption on *l*. We reach thus a contradiction that implies that there does not exist any pair of non-isomorphic fully symmetric trees with the same *e*^*n*^-size. By Lemma 10, this implies that $C_{D,e^{n}}$ is sound.

The same argument shows that $C_{D,f}$ is sound for every exponential function *f*(*n*) = *r*^*n*^ with base *r* a transcendental real number. However, if *r* is not transcendental, then $C_{D,r^{n}}$ need not be sound. For instance, $\delta_{2^{n}}(FS_{2,3})=\delta_{2^{n}}(FS_{3,2})=26$ and $\delta_{{\sqrt{2}}^{n}}\left(FS_{8,10}\right)=\delta_{{\sqrt{2}}^{n}}\left(FS_{12,8}\right)=352$.

**Proposition 17**. *If f*(*n*) = ln(*n* + *e*), *then* $C_{D,f}$ *is sound*.

**Proof**. The argument is similar to that of the previous proof. Let *f*(*n*) = ln(*n* + *e*) and assume that there exist two non-isomorphic fully symmetric trees $FS_{n_{1},\ldots ,n_{k}}$ and $FS_{m_{1},\ldots ,m_{l}}$ such that $\delta_{f}\left(FS_{n_{1},\ldots ,n_{k}}\right)=\delta_{f}\left(FS_{m_{1},\ldots ,m_{l}}\right)$, that is, such that

$$\begin{array}{c} \begin{array}{c} n_{1}\cdots n_{k}+n_{1}\cdots n_{k-1}\;\text{ln}\left(n_{k}+e\right)+\cdots +\text{ln}\left(n_{1}+e\right)\\ \;=m_{1}\cdots m_{l}+m_{1}\cdots m_{l-1}\;\text{ln}\left(m_{l}+e\right)+\cdots +\text{ln}\left(m_{1}+e\right). \end{array} \end{array}$$

Assume that *l* is the smallest depth of a fully symmetric tree with *f*-size equal to the *f*-size of a fully symmetric tree non-isomorphic to it.

Applying the exponential function to both sides of the equality above, we obtain

$$\begin{array}{c} \begin{array}{c} e^{n_{1}\cdots n_{k}}{\left(n_{k}+e\right)}^{n_{1}\cdots n_{k-1}}\cdots {\left(n_{2}+e\right)}^{n_{1}}\left(n_{1}+e\right)\\ \;\;=e^{m_{1}\cdots m_{l}}{\left(m_{l}+e\right)}^{m_{1}\cdots m_{l-1}}\cdots {\left(m_{2}+e\right)}^{m_{1}}\left(m_{1}+e\right). \end{array} \end{array}$$

Since *e* is transcendental, this implies the equality of polynomials in Z[x]

$$\begin{array}{c} \begin{array}{c} x^{n_{1}\cdots n_{k}}{\left(n_{k}+x\right)}^{n_{1}\cdots n_{k-1}}\cdots {\left(n_{2}+x\right)}^{n_{1}}\left(n_{1}+x\right)\\ \;\;=x^{m_{1}\cdots m_{l}}{\left(m_{l}+x\right)}^{m_{1}\cdots m_{l-1}}\cdots {\left(m_{2}+x\right)}^{m_{1}}\left(m_{1}+x\right), \end{array} \end{array}$$

which, since *n*_1_, …, *n*_*k*_, *m*_1_, …, *m*_*l*_ ≥ 2, on its turn implies the equalities

$$\begin{array}{c} \begin{array}{c} x^{n_{1}\cdots n_{k}}=x^{m_{1}\cdots m_{l}},\;\text{i.e.,}\;n_{1}\cdots n_{k}=m_{1}\cdots m_{l},\\ {\left(x+n_{k}\right)}^{n_{1}\cdots n_{k-1}}\cdots {\left(x+n_{2}\right)}^{n_{1}}\left(x+n_{1}\right)\\ \;\;={\left(x+m_{l}\right)}^{m_{1}\cdots m_{l-1}}\cdots {\left(x+m_{2}\right)}^{m_{1}}\left(x+m_{1}\right). \end{array} \end{array}$$

If *l* = 1, the right-hand side polynomial in the second equality is simply *x* + *m*_1_ and then this equality of polynomials implies that *k* = 1 and *n*_1_ = *m*_1_, which contradicts the assumption that $FS_{n_{1},\ldots ,n_{k}}\neq FS_{m_{1},\ldots ,m_{l}}$. Now assume that *l* ≥ 2. From the first equality we know that *n*_1_⋯*n*_*k*_ = *m*_1_⋯*m*_*l*_. But, the root of the left-hand side polynomial in the second equality with largest multiplicity is −*n*_*k*_ (because, by Lemma 15, *n*_1_⋯*n*_*k*−1_ alone is greater than the degree of ${\left(x+n_{k-1}\right)}^{n_{1}\cdots n_{k-2}}\cdots {\left(x+n_{2}\right)}^{n_{1}}\left(x+n_{1}\right)$) and, similarly, the root of the right-hand side polynomial in the second equality with largest multiplicity is −*m*_*l*_. Therefore, the equality of both polynomials implies that *n*_*k*_ = *m*_*l*_ and hence, by Lemma 14, $\delta_{f}\left(FS_{n_{1},\ldots ,n_{k-1}}\right)=\delta_{f}\left(FS_{m_{1},\ldots ,m_{l-1}}\right)$, against the assumption on *l*. As in the previous proof, this contradiction implies that $C_{D,f}$ is sound.

The same argument proves that, for every transcendental number *r* > 1, the function *f*(*n*) = log_*r*_(*n* + *r*) defines sound indices $C_{D,f}$. However, if *r* is not transcendental, then such a $C_{D,f}$ need not be sound. For instance, $\delta_{{\text{log}}_{2}(n+2)}(FS_{9,6})=\delta_{{\text{log}}_{2}(n+2)}(FS_{20,2})=81+{\text{log}}_{2}(11)$.

Summarizing, each one of the functions *f*(*n*) = ln(*n* + *e*) and *f*(*n*) = *e*^*n*^ defines, for every dissimilarity *D*, a Colless-like index $C_{D,f}$ that reaches its minimum value on each $T_{n}^{*}$, 0, at exactly the fully symmetric trees.

## Results

### Maximally unbalanced trees

The next results give the maximum values of $C_{D,f}$ on $T_{n}^{*}$ when *D* = MDM, var or *sd* and *f*(*n*) = ln(*n*+ *e*) or *f*(*n*) = *e*^*n*^. These maxima define the range of each $C_{D,f}$ on $T_{n}^{*}$, and, dividing by them, we can define normalized Colless-like indices that can be used to compare the balance of trees with different numbers of leaves.

We begin with the function *f*(*n*) = ln(*n* + *e*), which is covered by the following theorem.

**Theorem 18**. *Let f be a function* $N\to R_{\geq 0}$ *such that* 0 < *f*(*k*) < *f*(*k* − 1) + *f*(2), *for every k* ≥ 3. *Then*, *for every n* ≥ 2, *the indices* $C_{\text{MDM},f}$, $C_{sd,f}$ *and* $C_{\text{var},f}$ *reach their maximum values on* $T_{n}^{*}$ *exactly at the comb K*_*n*_. *These maximum values are*, *respectively*,

$$\begin{array}{c} \begin{array}{c} C_{\text{MDM},\delta_{f}}\left(K_{n}\right)=\frac{f(0)+f(2)}{4}\left(n-1\right)\left(n-2\right),\\ C_{sd,\delta_{f}}\left(K_{n}\right)=\frac{f(0)+f(2)}{2\sqrt{2}}\left(n-1\right)\left(n-2\right),\\ C_{\text{var},\delta_{f}}\left(K_{n}\right)=\frac{{\left(f\left(0\right)+f\left(2\right)\right)}^{2}}{12}\left(n-1\right)\left(n-2\right)\left(2n-3\right). \end{array} \end{array}$$

The proof of this theorem is very long, and we devote to it the first three sections in S1 File, one section for each dissimilarity.

It is straightforward to check that the function *f*(*n*) = ln(*n* + *e*) satisfies the hypothesis of Theorem 18 (for the inequality *f*(*k*) ≤ *f*(*k* − 1) + *f*(2), notice that ln(*k* + *e*) ≤ ln(*k* + *e* − 1) + ln(2) if, and only if, *k* + *e* ≤ 2(*k* + *e* − 1), and this last inequality is true for every k∈N). Therefore, $C_{\text{MDM},\text{ln}(n+e)}$, $C_{\text{var},\text{ln}(n+e)}$, and $C_{sd,\text{ln}(n+e)}$ take their maximum values on $T_{n}^{*}$ at the comb *K*_*n*_. In other words, the combs are the most unbalanced trees according to these indices. Table B in S2 File gives the values of $C_{\text{MDM},\text{ln}(n+e)}$, $C_{\text{var},\text{ln}(n+e)}$, and $C_{sd,\text{ln}(n+e)}$ on $T_{n}^{*}$, for *n* = 2, 3, 4, 5, and the positions of the different trees in each $T_{n}^{*}$ according to the increasing order of the corresponding index.

And for *f*(*n*) = *e*^*n*^, we have the following result. We have also moved its proof to the S1 File.

**Fig 7:**
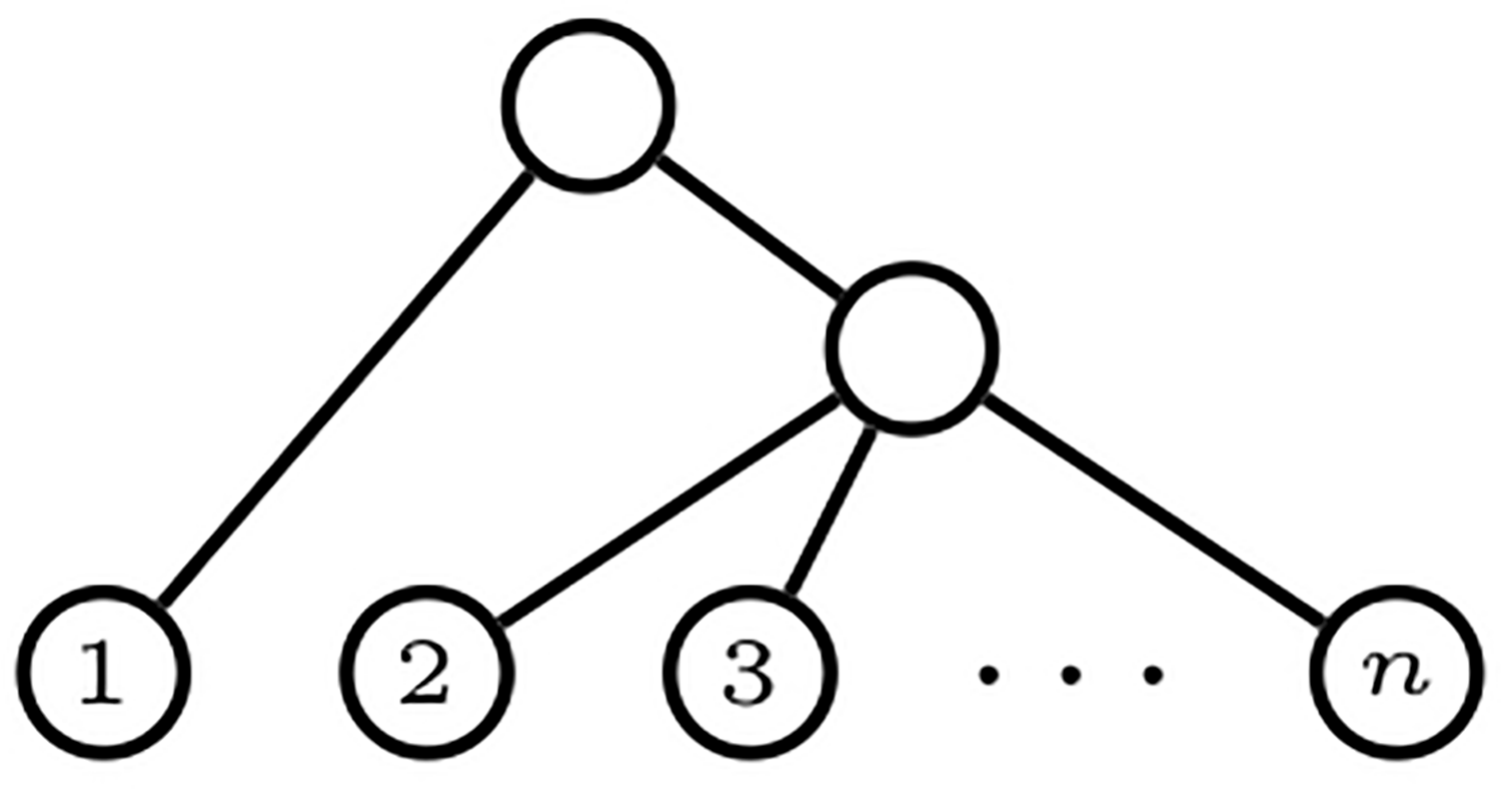
The tree *FS*_1_ ⋆ *FS*_*n*−1_.

**Theorem 19**. *For every* *n* ≥ 2:

(*a*) *If n* ≠ 4, *then both* $C_{\text{MDM},e^{n}}$ *and* $C_{sd,e^{n}}$ *reach their maximum on* $T_{n}^{*}$ *exactly at the tree FS*_1_ ⋆ *FS*_*n*−1_ (*see* Fig 7), *and these maximum values are*
  $$\begin{array}{c} \begin{array}{c} C_{\text{MDM},e^{n}}\left(FS_{1}⋆FS_{n-1}\right)=\frac{1}{2}\left(e^{n-1}+n-2\right),\\ C_{sd,e^{n}}\left(FS_{1}⋆FS_{n-1}\right)=\frac{1}{\sqrt{2}}\left(e^{n-1}+n-2\right). \end{array} \end{array}$$
(*b*) *Both* $C_{\text{MDM},e^{n}}$ *and* $C_{sd,e^{n}}$ *reach their maximum on* $T_{4}^{*}$ *exactly at the comb K*_4_, *and these maximum values are*
  $$\begin{array}{c} \begin{array}{c} C_{\text{MDM},e^{n}}\left(K_{4}\right)=\frac{3}{2}\left(e^{2}+1\right),\\ C_{sd,e^{n}}\left(K_{4}\right)=\frac{3}{\sqrt{2}}\left(e^{2}+1\right). \end{array} \end{array}$$
(*c*) $C_{\text{var},e^{n}}$ *always reaches its maximum on* $T_{n}^{*}$ *exactly at the tree FS*_1_ ⋆ *FS*_*n*−1_, *and the maximum value is*
  $$C_{\text{var},e^{n}}\left(FS_{1}⋆FS_{n-1}\right)=\frac{1}{2}{\left(e^{n-1}+n-2\right)}^{2}.$$

So, according to $C_{\text{MDM},e^{n}}$, $C_{\text{var},e^{n}}$, and $C_{sd,e^{n}}$, the trees of the form *FS*_1_ ⋆ *FS*_*n*−1_ are the most unbalanced (except for *n* = 4 and *D* = MDM or *sd*, in which case the most unbalanced tree is the comb). Table B in S2 File also gives the values of these indices on $T_{n}^{*}$, for *n* = 2, 3, 4, 5, and the positions of the different trees in each $T_{n}^{*}$ according to the increasing order of the corresponding index.

### The R package “CollessLike”

We have written an R package called *CollessLike*, available at the CRAN (https://cran.r-project.org/web/packages/CollessLike/index.html), that computes the Colless-like indices and their normalized version, as well as several other balance indices, and simulates the distribution of these indices on $T_{n}$ under the *α*-*γ*-model [10]. This package contains functions that:

- Compute the following balance indices for multifurcating trees: the Sackin index *S* [4–6], the total cophenetic index Φ [8], and the Colles-like index $C_{D,f}$ for several predefined dissimilarities *D* and functions *f* as well as for any user-defined ones.
  Our functions also compute the normalized versions (obtained by subtracting their minimum value and dividing by the width of their range, so that they take values in [0, 1]) of *S*, Φ and the Colless-like indices $C_{D,f}$ for which we have computed the range in Theorems 18 and 19. Recall from the aforementioned references that, for every *n* ≥ 2:
  - the range of *S* on $T_{n}^{*}$ is *S*(*FS*_*n*_) = *n* to $S\left(K_{n}\right)=\frac{1}{2}\left(n+2\right)\left(n-1\right)$
  - the range of Φ on $T_{n}^{*}$ is Φ(*FS*_*n*_) = 0 to $\Phi \left(K_{n}\right)=(\frac{n}{3})$
  Therefore, for every $T\in T_{n}$, the normalized Sackin and total cophenetic index are, respectively,
  $$S_{norm}\left(T\right)=\frac{S\left(T\right)-n}{\frac{1}{2}\left(n+2\right)\left(n-1\right)-n},\;\Phi_{norm}\left(T\right)=\frac{\Phi \left(T\right)}{\left(\begin{array}{c} n\\ 3 \end{array}\right)},$$
  while, for instance, the normalized version of $C_{\text{MDM},\text{ln}(n+e)}$ is
  $$C_{\text{MDM},\text{ln}(n+e),norm}\left(T\right)=\frac{C(T)}{\frac{1+\text{ln}(e+2)}{4}\left(n-1\right)\left(n-2\right)}.$$
- Given two natural numbers *n* and *N*, produce a random sample of *N* values of a balance index *S*, Φ, or $C_{D,f}$ on trees in $T_{n}$ generated following an *α*-*γ*-model: the parameters *N*, *n*, *α*, *γ* (with 0 ≤ *γ* ≤ *α* ≤ 1) can be set by the user.
  Due to the computational cost of this function, we have stored the values of *S*, Φ, and $C_{\text{MDM},\text{ln}(n+e)}$ (denoted henceforth simply by C) on the random samples of *N* = 5000 trees in each $T_{n}$ (for every *n* = 3, …, 50 and for every *α*, *γ* ∈ {0, 0.1, 0.2, …, 0.9, 1} with *γ* ≤ *α*) generated in the study reported in the next subsection. In this way, if the user is interested in this range of numbers of leaves and this range of parameters, he or she can study the distribution of the corresponding balance index efficiently and quickly.
- Given a tree $T\in T_{n}$, estimate the percentile *q*_*T*,*n*,*α*,*γ*_ of its balance index *S*, Φ, or $C_{D,f}$ with respect to the distribution of this index on $T_{n}$ under some *α*-*γ*-model. If *n*, *α*, *γ* are among those mentioned in the previous item, for the sake of efficiency this function uses the database of computed indices to simulate the distribution of the balance index on $T_{n}$ under this *α*-*γ*-model.

**Fig 8:**
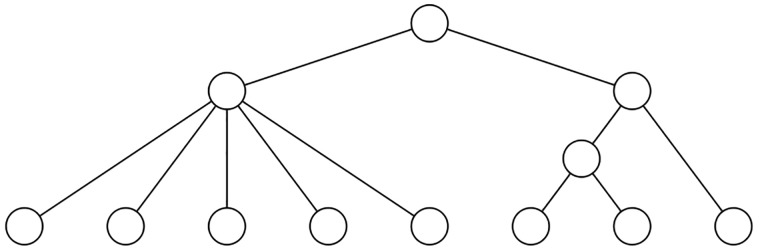
A tree with 8 leaves randomly generated under the *α*-*γ*-model with *α* = 0.7 and *γ* = 0.4. Its indices are C(T)=1.746, *S*(*T*) = 18, and Φ(*T*) = 14, and its normalized indices are $C_{norm}\left(T\right)=0.06518$, *S*_*norm*_(*T*) = 0.3704, and Φ_*norm*_(*T*) = 0.25.

For instance, the unlabeled tree $T\in T_{8}^{*}$ in Fig 8 is the shape of a phylogenetic tree randomly generated under the *α*-*γ*-model with *α* = 0.7 and *γ* = 0.4 (using set.seed(1000) for reproducibility). The values of its balance indices are given in the figure’s caption.

**Fig 9:**
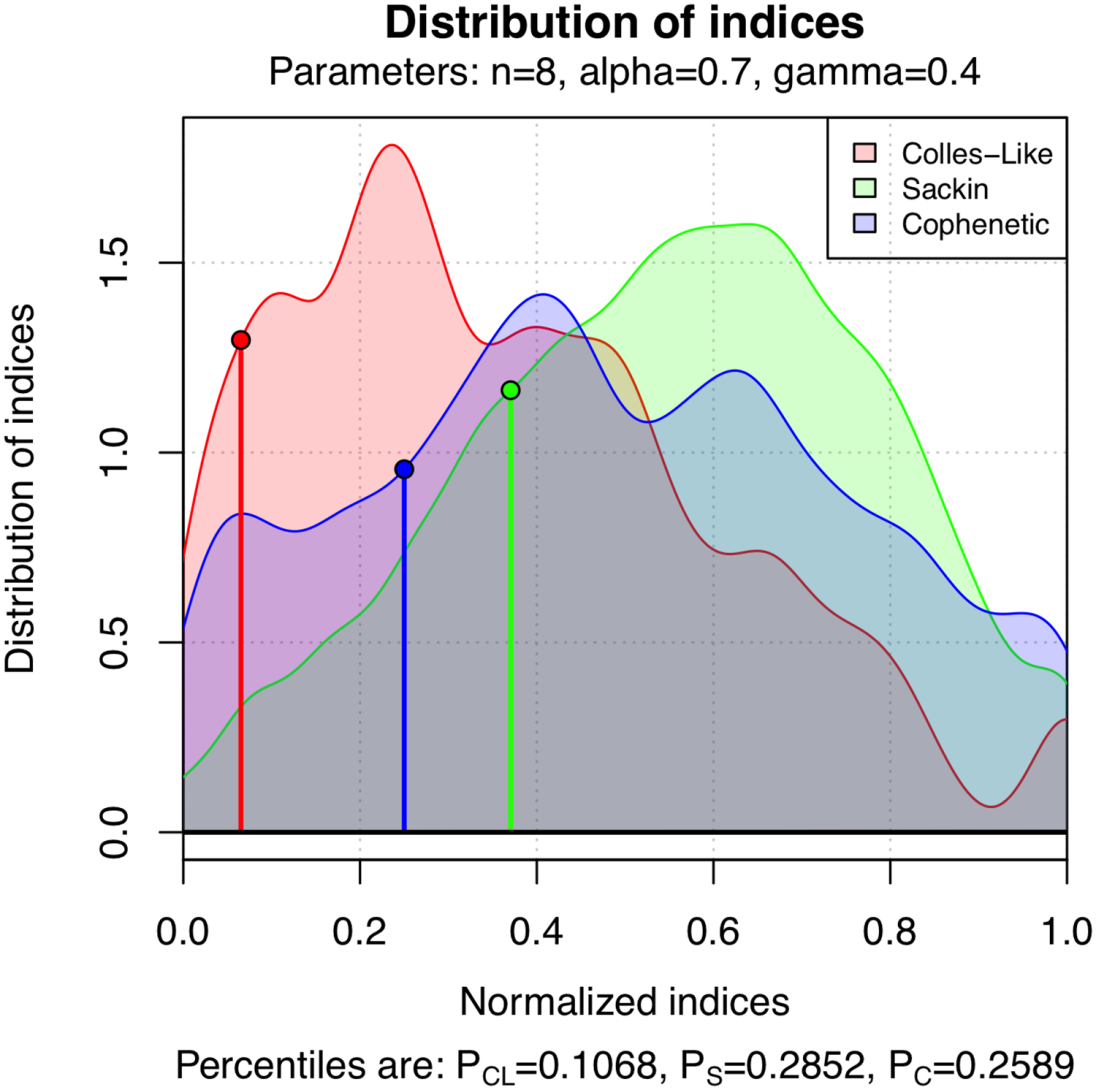
The estimated density function of the distribution of C, *S* and Φ on $T_{8}$ under the *α*-*γ*-model with *α* = 0.7 and *γ* = 0.4. The percentiles of the tree in Fig 8 are also represented.

Fig 9 displays the estimation of the density function of the balance indices C, *S*, and Φ under the *α*-*γ*-model with *α* = 0.7 and *γ* = 0.4 on $T_{8}$, obtained using the 5000 random trees gathered in our database. Moreover, the estimated percentiles of the balance indices of the tree of Fig 8 are also shown in the figure.

**Fig 10:**
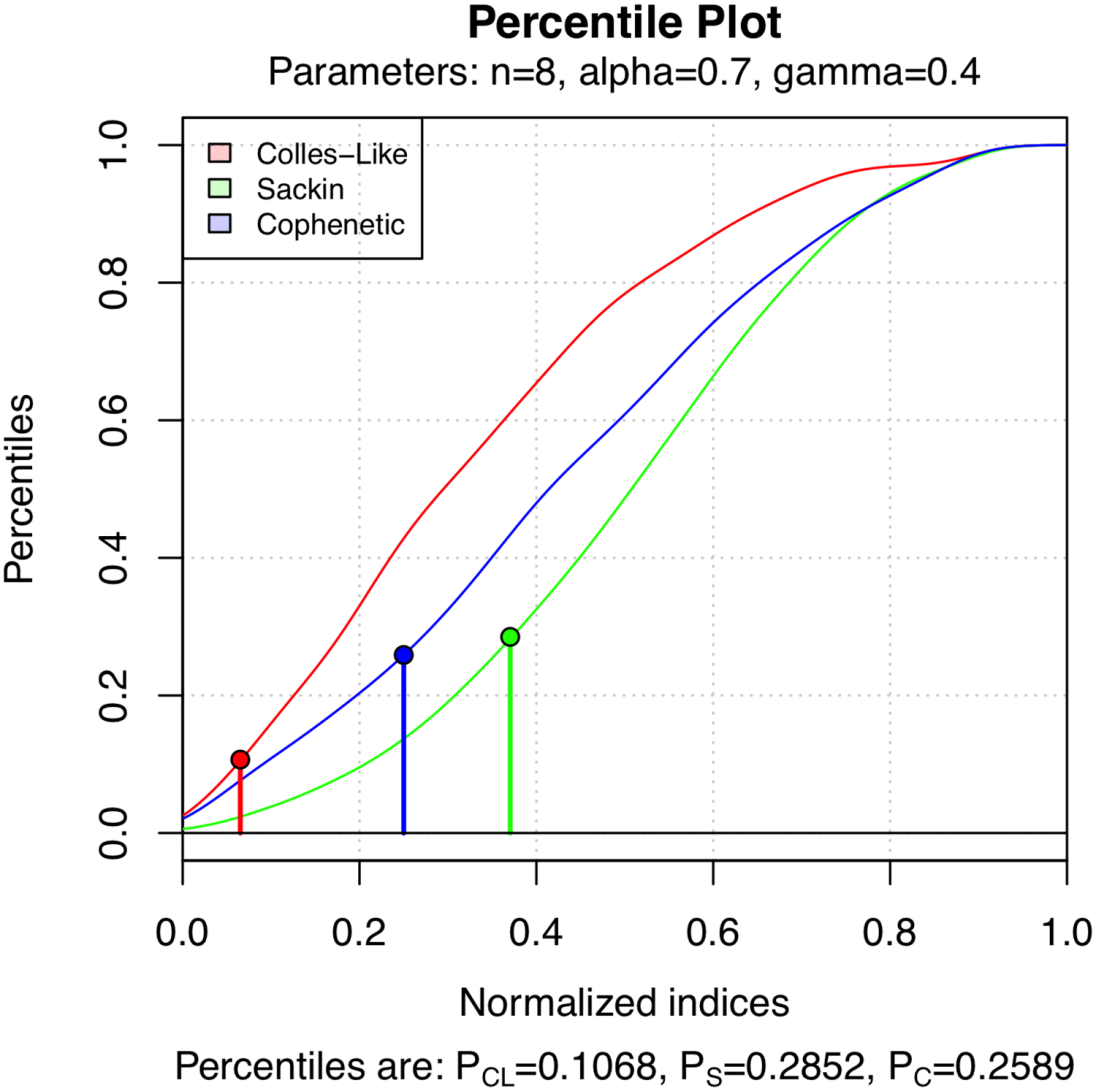
Percentile plot of the distribution of C, *S* and Φ on $T_{8}$ under the *α*-*γ*-model with *α* = 0.7 and *γ* = 0.4. The percentiles of the tree of Fig 8 are also highlighted.

Fig 10 shows a percentile plot of C, *S*, and Φ under the *α*-*γ*-model for *α* = 0.7 and *γ* = 0.4 on $T_{8}$. The percentiles of the tree of Fig 8 are given by the area to the left of the vertical lines.

**Fig 11:**
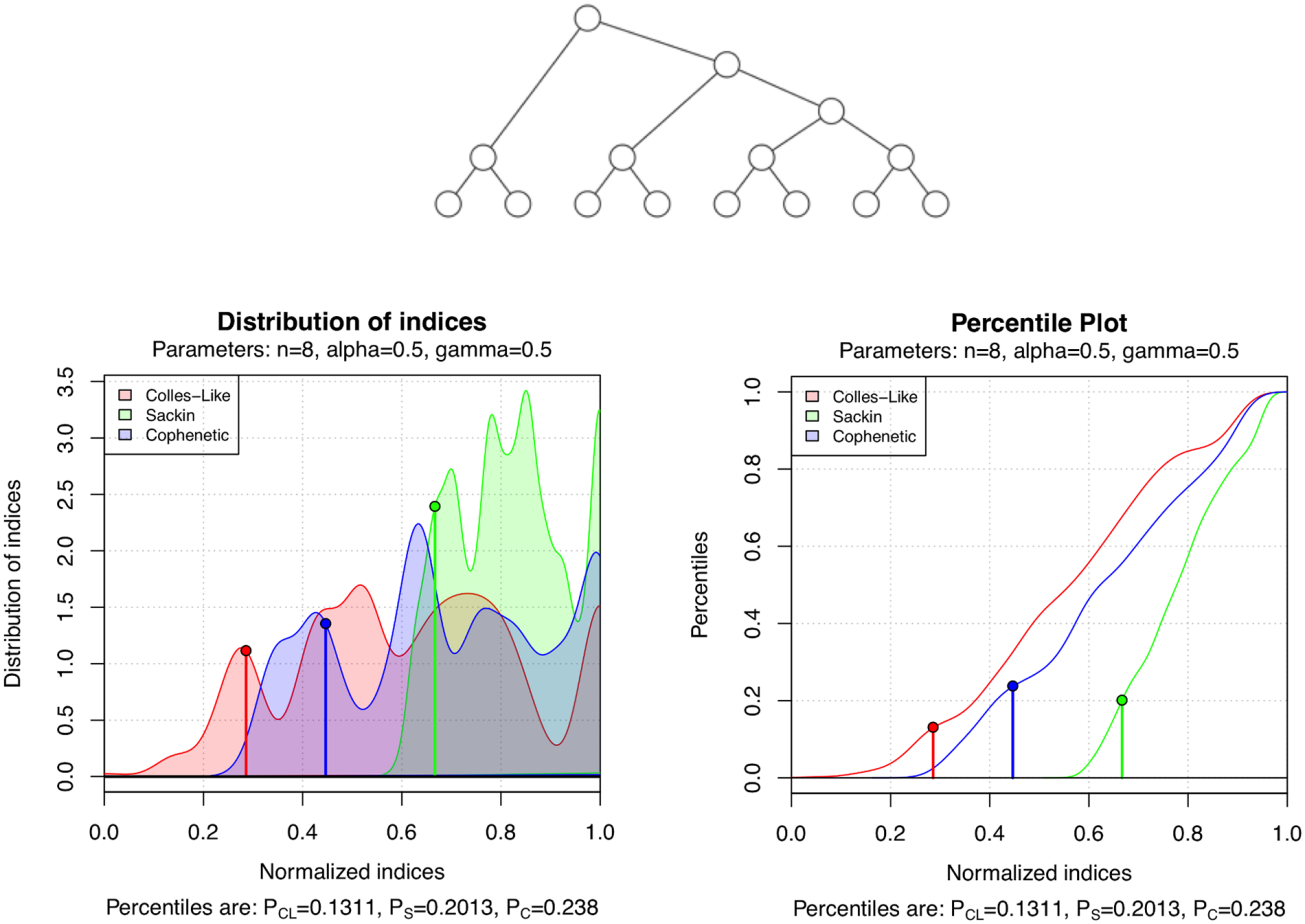
A bifurcating tree randomly generated under the uniform model, the estimated density function of the distribution of the three balance indices on $T_{8}$ under the uniform model, and their percentile plot.

A special case of the *α*-*γ*-model, corresponding to the case *α* = *γ*, is Ford’s *α*-model for bifurcating phylogenetic trees [17]. This model includes as special cases the Yule, or Equal-Rate Markov, model [18, 19] and the uniform, or Proportional to Distinguishable Arrangements, model [20, 21]. So, this package allows also to study this model. For example, the unlabeled tree in Fig 11 has been generated (with set.seed(1000)) using *n* = 8 and *α* = *γ* = 0.5, which corresponds to the uniform model. The figure also depicts the estimation of the density functions and of the percentile plots of C, *S*, and Φ on $T_{8}$ under this model, as well as the percentile values of the tree.

### Experimental results on TreeBASE

To assess the performance of $C_{\text{MDM},\text{ln}(n+\text{e})}$, which we abbreviate by C, we downloaded (December 13-14, 2015) all phylogenetic trees in the TreeBASE database [11] using the function search_treebase() of the R package treebase [22]. We obtained 13,008 trees, from which 80 had format problems that prevented R from reading them, so we restricted ourselves to the remaining 12,928 trees. To simplify the language, we shall still refer to this slightly smaller subset of phylogenetic trees as “all trees in TreeBASE”. Only 4,814 among these 12,928 trees in TreeBASE are bifurcating.

Then, for every phylogenetic tree *T* in this set, we have computed its Colless-like index C(T), its Sackin index *S*(*T*), and its total cophenetic index Φ(*T*). We have compared the results in the ways we show next (all analysis have been performed with R [23]).

#### Behavior as functions of the number of leaves

For every number of leaves *n*, we have computed the mean and the variance of C, *S* and Φ on all trees with *n* leaves in TreeBASE. Then, we have computed the regression of these values as a function of *n*.

For the means, the best fits have been:

- *Colless-like index*: $\bar{C}\approx 0.5351\cdot n^{1.5848}$, with a coefficient of determination of *R*^2^ = 0.9869 and a p-value for the exponent *p* < 2 ⋅ 10^−16^.
- *Sackin index*: $\bar{S}\approx 1.4512\cdot n^{1.4359}$, with a coefficient of determination of *R*^2^ = 0.9953 and a p-value for the exponent *p* < 2 ⋅ 10^−16^.
- *Total cophenetic index*: $\bar{\Phi }\approx 0.1894\cdot n^{2.5478}$, with a coefficient of determination of *R*^2^ = 0.9945 and a p-value for the exponent *p* < 2 ⋅ 10^−16^.

**Fig 12:**
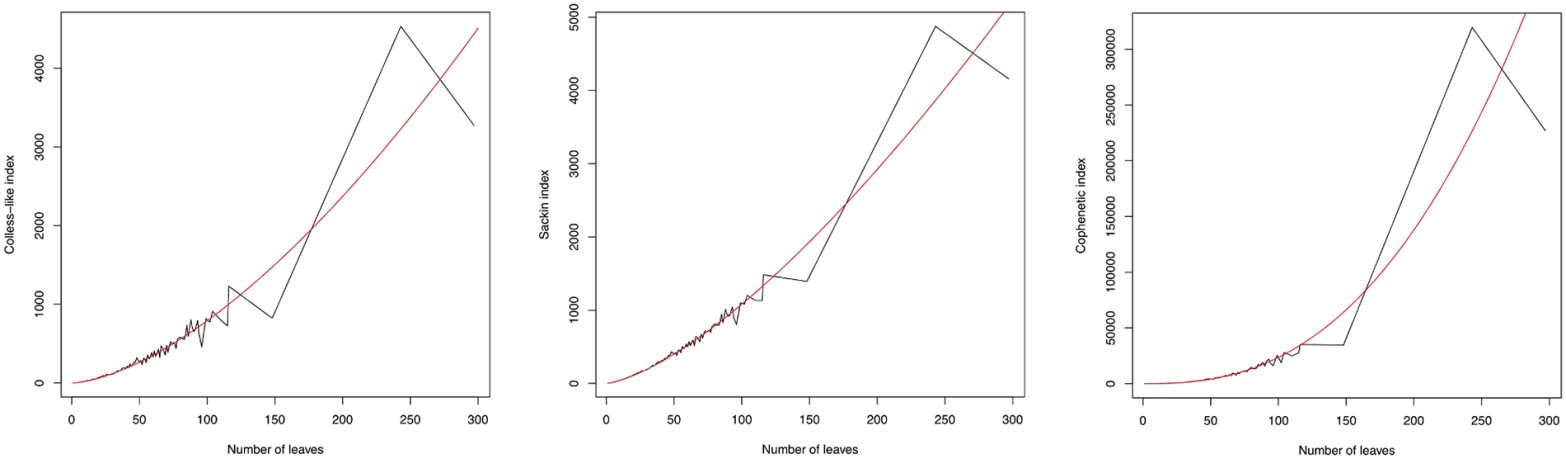
Growth of the mean value of C (left), *S* (center), and Φ (right) in TreeBASE, as functions of the trees’ numbers of leaves *n*.

Fig 12 depicts these mean values of C (left), *S* (center), and Φ (right) as functions of *n*.

Thus, *S* and C have similar mean growth rates, while Φ has a mean growth rate one order higher in magnitude. This difference vanishes if we normalize the indices by their range width, which is *O*(*n*^2^) for C and *S*, and *O*(*n*^3^) for Φ:

$$\begin{array}{c} \begin{array}{c} \bar{C_{norm}}\approx 0.8389\cdot n^{-0.4152}\\ \bar{S_{norm}}\approx 2.9024\cdot n^{-0.5641}\\ \bar{\Phi_{norm}}\approx 1.1364\cdot n^{-0.4522} \end{array} \end{array}$$

As for the behavior of the variances, the best fits are the following:

- *Colless index*: $\text{var}\left(C\right)\approx 0.07599\cdot n^{3.12831}$, with a coefficient of determination of *R*^2^ = 0.962 and a p-value for the exponent *p* < 2 ⋅ 10^−16^.
- *Sackin index*: Var(*S*) ≈ 0.03182 ⋅ *n*^3.22441^, with a coefficient of determination of *R*^2^ = 0.9575 and a p-value for the exponent *p* < 2 ⋅ 10^−16^.
- *Total cophenetic index*: Var(Φ) ≈ 0.0041 ⋅ *n*^5.2075^, with a coefficient of determination of *R*^2^ = 0.9812 and a p-value for the exponent *p* < 2 ⋅ 10^−16^.

The results are in the same line as before, with the variances of C and *S* having similar growth rates, and the variance of Φ having a growth rate two orders of magnitude higher. This difference vanishes again when we normalize the indices:

$$\begin{array}{c} \begin{array}{c} \text{var}\left(C_{norm}\right)\approx 0.18677\cdot n^{-0.87169}\\ \text{var}\left(S_{norm}\right)\approx 0.12728\cdot n^{-0.77559}\\ \text{var}\left(\Phi_{norm}\right)\approx 0.1476\cdot n^{-0.7925} \end{array} \end{array}$$

So, in summary, C has, on TreeBASE and relative to the range of values, a slightly larger mean growth rate and a slightly smaller variance growth rate than the other two indices.

#### Numbers of ties

The number of ties (that is, of pairs of different trees with the same index value) of a balance index is an interesting measure of quality, because the smaller its frequency of ties, the bigger its ability to rank the balance of any pair of different trees. Although, in our opinion, this ability need not always be an advantage: for instance, neither Φ nor *S* take the same, minimum, value on all different fully symmetric trees with the same numbers of leaves (for example, *S*(*FS*_6_) = 6 but *S*(*FS*_2,3_) = *S*(*FS*_3,2_) = 12; and Φ(*FS*_6_) = 0, but Φ(*FS*_3,2_) = 3 and Φ(*FS*_2,3_) = 6; cf. Fig 5), while C applied to any fully symmetric tree is always 0. In this case, we believe that these ties are fair.

**Fig 13:**
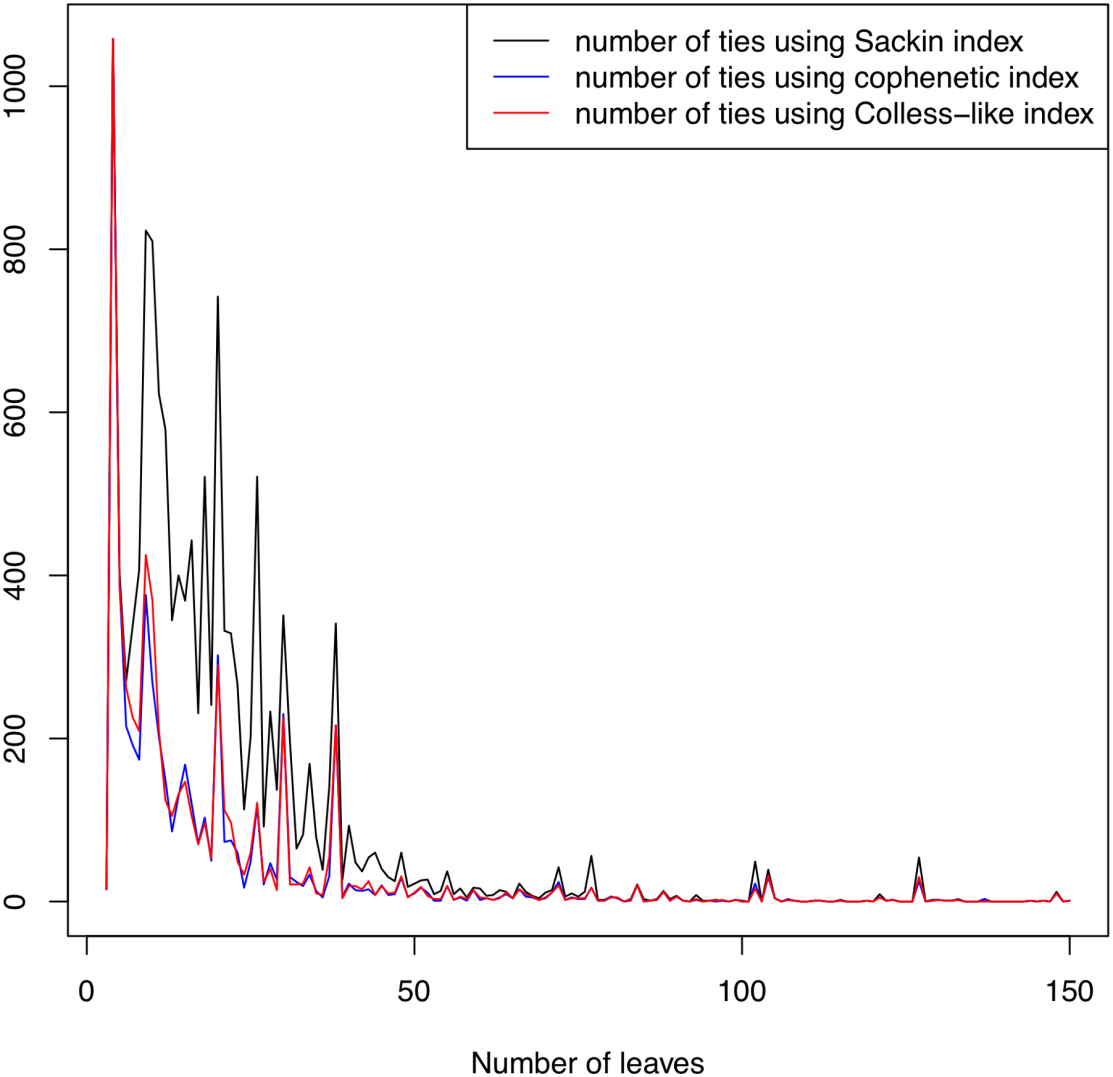
Numbers of ties of C, *S*, and Φ in TreeBASE, as functions of the trees’ numbers of leaves *n*.

Anyway, for every number of leaves *n* and for every one of all three indices under scrutiny, we have computed the numbers of pairs of trees with *n* leaves in TreeBASE having the same value of the corresponding index (in the case of C, up to 16 decimal digits). Fig 13 plots the frequencies of ties of C, *S* and Φ as functions of *n*. As it can be seen in this graphic, C and Φ have a similar number of ties, and consistently less ties than *S*.

#### Spearman’s rank correlation

In order to measure whether all three indices sort the trees according to their balance in the same way or not, we have computed the Spearman’s rank correlation coefficient [24] of the indices on all trees in TreeBASE, as well as grouping them by their number of leaves *n*.

**Fig 14:**
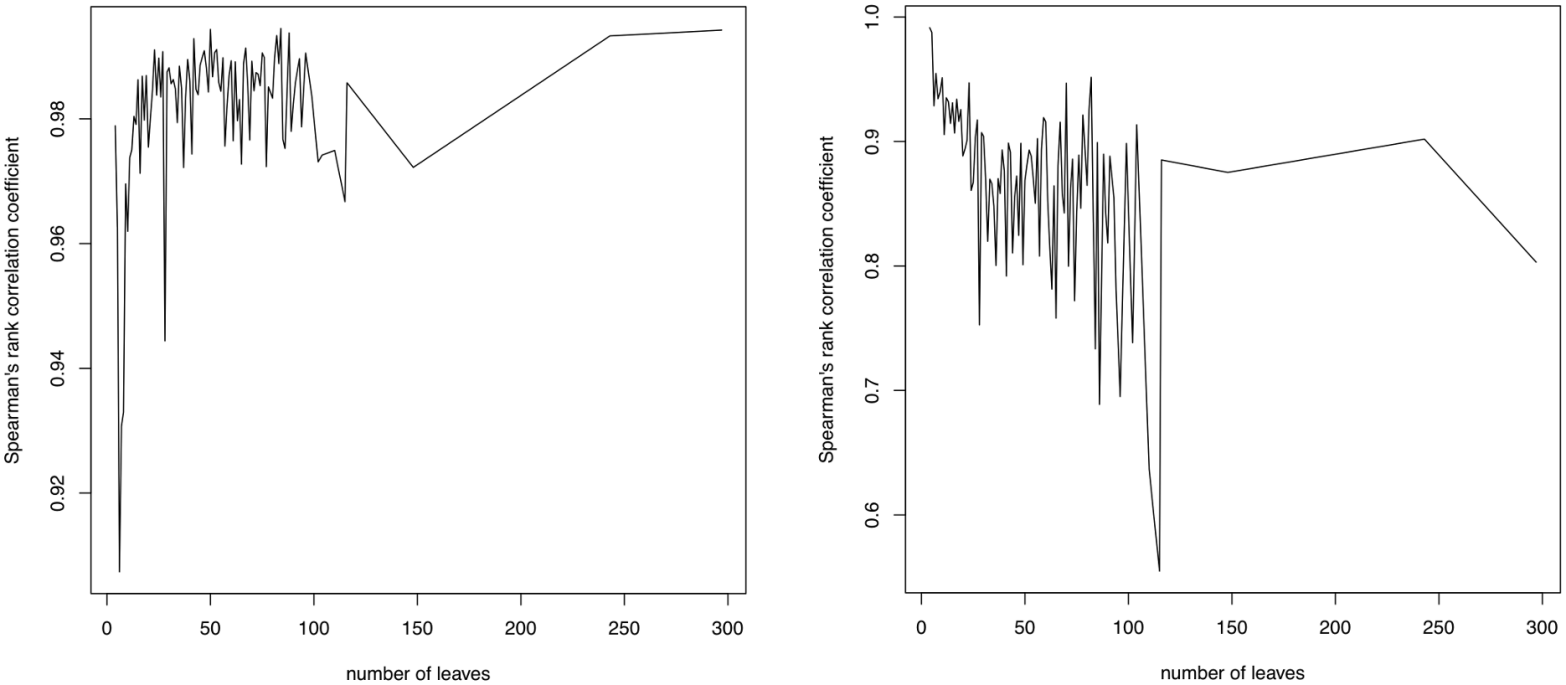
Spearman’s rank correlation coefficient of C and *S* (left) and of C and Φ (right) in TreeBASE, as functions of the trees’ numbers of leaves *n*.

The global Spearman’s rank correlation coefficient of C and *S* is 0.9765, and that of C and Φ is 0.9619. The graphics in Fig 14 plot these coefficients as functions of *n*. As it can be seen, Spearman’s rank correlation coefficient for C and *S* grows with *n*, approaching to 1, while the coefficient for C and Φ shows a decreasing tendency with *n*.

### Does TreeBASE fit the uniform model or the alpha-gamma model?

In this subsection, we test whether the distribution of the Colless-like index of the phylogenetic trees in TreeBASE agrees with its theoretical distribution under either the uniform model for multifurcating phylogenetic trees [25] or the *α*-*γ*-model [10] for some parameters *α*, *γ*. To do it, we use the normalized version $C_{norm}$ of C, which can be used simultaneously on trees with different numbers of leaves.

To estimate the theoretical distribution of this index under the two aforementioned theoretical models, for every *n* = 3, …, 50 we have generated, on the one hand, 10,000 random phylogenetic trees in $T_{n}$ under the uniform model using the algorithm described in [25], and, on the other hand, 5000 random phylogenetic trees in $T_{n}$ under the *α*-*γ*-model for every pair of parameters (*α*, *γ*)∈{0, 0.1, 0.2, …, 0.9, 1}^2^ with *γ* ≤ *α*. We have computed the value of $C_{norm}$ on all these trees, and we have used the distribution of these values as an estimation of the corresponding theoretical distribution. To test whether the distribution of the normalized Colless-like index on TreeBASE (or on some subset of it: see below) fits one of these theoretical distributions, we have performed two non-parametric statistical tests on the observed set of indices of TreeBASE and the corresponding simulated set of indices: Pearson’s chi-squared test and the Kolmogorov-Smirnov test, using bootstrapping techniques in the latter to avoid problems with ties.

**Fig 15:**
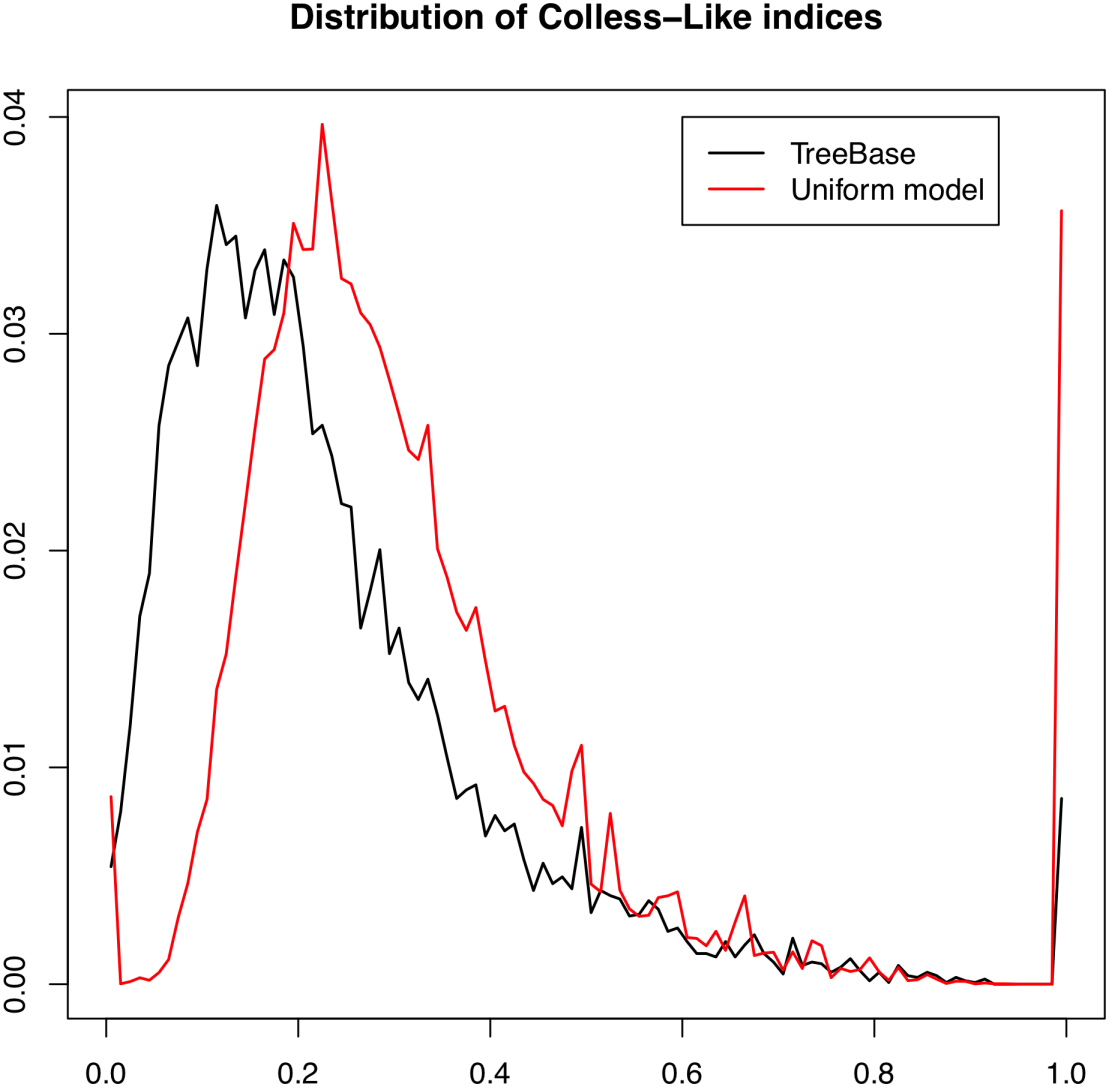
The distribution of $C_{norm}$ on all trees in TreeBASE (black line) and its estimated theoretical distribution under the uniform model (red line).

As a first approach, we have performed these tests on the whole set of trees in TreeBASE. The p-values obtained in all tests, be it for the uniform model or for any considered pair (*α*, *γ*), have turned out to be negligible. Then, we conclude confidently that the distribution of the normalized Colless-like index on TreeBASE does not fit either the uniform model or any *α*-*γ*-model when we round *α*, *γ* to one decimal place. For instance, Fig 15 displays the distribution of $C_{norm}$ on TreeBASE and its estimated theoretical distribution under the uniform model. As it can be seen, these distributions are quite different, which confirms the conclusion of the statistical test.

**Fig 16:**
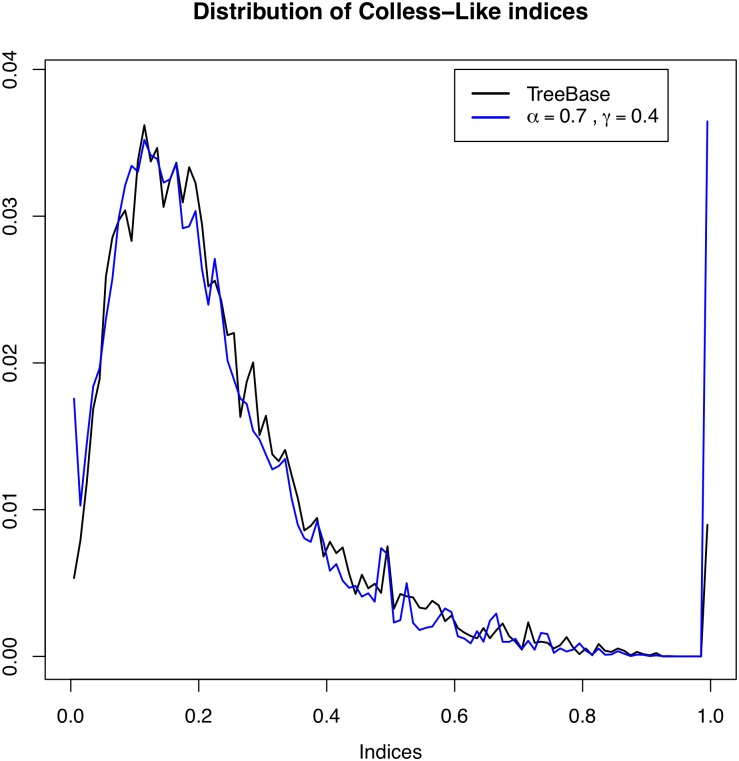
The distribution of $C_{norm}$ on all trees in TreeBASE (black line) and its estimated theoretical distribution under the *α*-*γ*-model with *α* = 0.7 and *γ* = 0.4 (blue line).

Fig 16 displays the distribution of $C_{norm}$ for all trees in TreeBASE and its estimated theoretical distribution under the *α*-*γ*-model for the pair of parameters *α*, *γ* that gave the largest p-values in the goodness of fit tests, which are *α* = 0.7 and *γ* = 0.4. Although graphically both distributions are quite similar, the p-values of the Pearson chi-squared test and of the Kolmogorov-Smirnov test are virtually zero. One might think that the high “peaks” of the theoretical distribution near 0 and 1 could have influenced the outcome of these statistical tests. For this reason, we have repeated them without taking into account these “extreme” values, and the results have been the same.

Since TreeBASE gathers phylogenetic trees of different types and from different sources, we have also considered subsets of it defined by means of attributes. More specifically, besides the whole TreeBASE as explained above, we have also considered the following subsets of it:

- All trees in TreeBASE up to repetitions: we have removed 513 repeated trees (which represent about a 4% of the total).
- All trees with their kind attribute equal to “Species”. This kind attribute can take three values: “Barcode tree”, “Gene Tree” and “Species Tree”.
- All trees with their kind attribute equal to “Species” and their type attribute equal to “Consensus”. This type attribute can take two values: “Consensus” and “Single”.
- All trees with their kind attribute equal to “Species” and their type attribute equal to “Single”.

We have repeated the study explained above for these four subsets of TreeBASE, comparing the distribution of the normalized Colless-like indices of their trees with the estimated theoretical distributions by means of goodness-of-fit tests, and the results have been the same, that is, all p-values have also turned out to be negligible. Our conclusion is, then, that neither the whole TreeBASE nor any of these four subsets of it seem to fit either the uniform model or some *α*-*γ*-model.

## Conclusions

In this paper we have introduced a family of *Colless-like* balance indices $C_{D,f}$, which depend on a dissimilarity *D* and a function $f:N\to R_{\geq 0}$, that generalize the Colless index to multifurcating phylogenetic trees. We have proved that every combination of a dissimilarity *D* and a function either *f*(*n*) = ln(*n* + *e*) or *f*(*n*) = *e*^*n*^, defines a Colless-like index that is *sound* in the sense that the maximally balanced trees according to it are exactly the fully symmetric ones. But, the growth of the function *f* determines strongly which are the most unbalanced trees according to $C_{D,f}$, and hence it has influence on the very notion of “balance” measured by the index.

In our opinion, choosing ln(*n* + *e*) instead of *e*^*n*^ seems a more sensible decision, because, on the one hand, the most unbalanced trees according to the former are the expected ones—the combs—and, on the other hand, we have encountered several hard numeric problems when working with the extremely large figures that appear when using *e*^*n*^-sizes on trees with internal nodes of high degree. With respect to the choice of the dissimilarity *D*, MDM and *sd* define indices that are proportional to the Colless index when applied to bifurcating trees. From these two options, we recommend to use MDM because it only involves linear operations, and hence it has less numerical precision problems than *sd*, that uses a square root of a sum of squares. This is the reason we have stuck to $C_{\text{MDM},\text{ln}(n+e)}$ in the numerical experiments reported in the Results section.

To end this paper, we would like to call the reader’s attention on the problem posed in the subsection “Sound Colless-like indices:” to find functions *f* such that $C_{D,f}$ is sound. Our conjecture is that there is no function f:N→N taking values in the set of natural numbers that satisfies this property.

## Supporting information

S1 File Proofs of Theorems 18 and 19. The file provides the detailed proofs of Theorems 18 and 19.
(PDF)

S2 FileTables.The file provides two tables, quoted in the main text, with the values of several Colless-like indices on $T_{n}^{*}$ for *n* = 2, 3, 4, 5.(PDF)
