# Supplementary material for: Sound Colless-like balance indices for multifurcating trees
Source: PLoS One. 2018 Sep 25;13(9):e0203401. doi: 10.1371/journal.pone.0203401 (PMC6155497; doi:10.1371/journal.pone.0203401)
Supplement: S1 File — The file provides the detailed proofs of Theorems 18 and 19. (PDF) [file pone.0203401.s001.pdf]

Sound Colless-like balance indices for multifurcating trees. Supplementary file S1: Proofs of Theorems 18 and 19

Arnau Mir, Francesc Rosselló, Lucía Rotger

This supplementary document contains the proofs of Theorems 18 (Sections A–C) and 19 (Sections D–F) in the main text.

A Proof of the thesis of Theorem 18 for $\mathfrak{C}_{\mathrm{MDM},f}$

Let in this section, and in the next two ones, $f:\mathbb{N}\to\mathbb{R}_{\geqslant 0}$ be any mapping such that $0<f(k)<f(k-1)+f(2)$ for every $k\geqslant 3$. Notice that if f satisfies this condition then $f(k)>0$ not only for every $k\geqslant 3$, but also for $k=2$, because $0<f(3)<2f(2)$. To simplify the notations, we shall denote in this section δ_f and $\mathfrak{C}_{\mathrm{MDM},f}$ by δ and $\mathfrak{C}$, respectively, and we shall denote $bal_{\mathrm{MDM},f}$ on a tree T by bal_T or simply by bal when it is not necessary to specify the tree.

We split this proof into several lemmas.

Lemma S1.1. *For every $(x_1,\ldots,x_{2n+1})\in\mathbb{R}^{2n+1}$ with $n\geqslant 1$, if x_i is the median of $\{x_1,\ldots,x_n\}$, then*

$$\mathrm{MDM}(x_1,\ldots,x_{2n+1})\leqslant \mathrm{MDM}(x_1,\ldots,x_{i-1},x_{i+1},\ldots,x_{2n+1}).$$

Moreover, this inequality is strict unless $x_1=\cdots=x_{2n+1}$.

Proof. After rearranging $x_1,\ldots,x_{2n+1}$ if necessary, we assume that $x_1\leqslant\cdots\leqslant x_{2n+1}$, in which case their median (i.e., their middle value) is x_{n+1}, and the median of $x_1,\ldots,x_n,x_{n+2},\ldots,x_{2n+1}$ is $M=(x_n+x_{n+2})/2$. We want to prove that

$$\mathrm{MDM}(x_1,\ldots,x_{2n+1})\leqslant \mathrm{MDM}(x_1,\ldots,x_n,x_{n+2},\ldots,x_{2n+1}).$$

This inequality is true, because

$$\begin{aligned}
\mathrm{MDM}(x_1,\ldots,x_{2n+1}) &= \frac{1}{2n+1}\sum_{i=1}^{2n+1}|x_i-x_{n+1}|\\
&= \frac{1}{2n+1}\Big(\sum_{i=1}^{n}(x_{n+1}-x_i)+\sum_{i=n+2}^{2n+1}(x_i-x_{n+1})\Big)=\frac{1}{2n+1}\Big(\sum_{i=n+2}^{2n+1}x_i-\sum_{i=1}^{n}x_i\Big)\\
\mathrm{MDM}(x_1,\ldots,x_n,x_{n+2},\ldots,x_{2n+1}) &= \frac{1}{2n}\Big(\sum_{i=1}^{n}|x_i-M|+\sum_{i=n+2}^{2n+1}|x_i-M|\Big)\\
&= \frac{1}{2n}\Big(\sum_{i=1}^{n}(M-x_i)+\sum_{i=n+2}^{2n+1}(x_i-M)\Big)=\frac{1}{2n}\Big(\sum_{i=n+2}^{2n+1}x_i-\sum_{i=1}^{n}x_i\Big),
\end{aligned}$$

and, clearly,

$$\frac{1}{2n+1}\Big(\sum_{i=n+2}^{2n+1}x_i-\sum_{i=1}^{n}x_i\Big)\leqslant\frac{1}{2n}\Big(\sum_{i=n+2}^{2n+1}x_i-\sum_{i=1}^{n}x_i\Big).$$

Moreover, the inequality is strict unless $\sum\limits_{i=n+2}^{2n+1}x_i=\sum\limits_{i=1}^{n}x_i$, which, under the assumption that $x_1\leqslant\cdots\leqslant x_{2n+1}$, is equivalent to $x_1=\cdots=x_{2n+1}$. □

Unfortunately, the thesis of this lemma is false for vectors of numbers of even length. For instance, consider the vector $(1,1,2,2)$: if we remove any single element, its MDM decreases. But, providentially, we can always increase the MDM of an even quantity of real numbers by removing *two* of them.

Lemma S1.2. *For every $(x_1,\dots,x_{2n})\in\mathbb{R}^{2n}$ with $n\geqslant 2$, if x_i,x_j, with $i<j$, are the middle values of $\{x_1,\dots,x_{2n}\}$, then*

$$\mathrm{MDM}(x_1,\dots,x_{2n})\leqslant \mathrm{MDM}(x_1,\dots,x_{i-1},x_{i+1},\dots,x_{j-1},x_{j+1},\dots,x_{2n}).$$

Moreover, this inequality is strict unless $\{x_1,\dots,x_{2n}\}$ consists either of $2n$ copies of a single element or of n copies of two different elements.

Proof. After rearranging $x_1,\dots,x_{2n}$ if necessary, we assume that $x_1\leqslant\cdots\leqslant x_{2n}$, so that their middle values are x_n,x_{n+1}, and hence their median is $M=(x_n+x_{n+1})/2$ and the median of $x_1,\dots,x_{n-1},x_{n+2},\dots,x_{2n}$ is $M'=(x_{n-1}+x_{n+2})/2$. We want to prove that

$$\mathrm{MDM}(x_1,\dots,x_{2n})\leqslant \mathrm{MDM}(x_1,\dots,x_{n-1},x_{n+2},\dots,x_{2n}).$$

And, indeed,

$$\begin{aligned}
&\mathrm{MDM}(x_1,\dots,x_{2n})\leqslant \mathrm{MDM}(x_1,\dots,x_{n-1},x_{n+2},\dots,x_{2n})\\
&\iff \frac{1}{2n}\sum_{i=1}^{2n}|x_i-M|\leqslant\frac{1}{2n-2}\Big(\sum_{i=1}^{n-1}|x_i-M'|+\sum_{i=n+2}^{2n}|x_i-M'|\Big)\\
&\iff (2n-2)\Big(\sum_{i=1}^{n}(M-x_i)+\sum_{i=n+1}^{2n}(x_i-M)\Big)\leqslant 2n\Big(\sum_{i=1}^{n-1}(M'-x_i)+\sum_{i=n+2}^{2n}(x_i-M')\Big)\\
&\iff (n-1)\Big(\sum_{i=n+1}^{2n}x_i-\sum_{i=1}^{n}x_i\Big)\leqslant n\Big(\sum_{i=n+2}^{2n}x_i-\sum_{i=1}^{n-1}x_i\Big)=n\Big(\sum_{i=n+1}^{2n}x_i-\sum_{i=1}^{n}x_i\Big)-n(x_{n+1}-x_n)\\
&\iff n(x_{n+1}-x_n)\leqslant\sum_{i=n+1}^{2n}x_i-\sum_{i=1}^{n}x_i=\sum_{i=1}^{n}(x_{n+i}-x_{n+1-i})
\end{aligned}$$

and this last inequality is true because $x_{n+1}-x_n\leqslant x_{n+i}-x_{n+1-i}$ for every $i=1,\dots,n$. Moreover, the inequality is strict unless $x_{n+1}-x_n=x_{n+i}-x_{n+1-i}$ for every $i=1,\dots,n$, that is, unless $x_1=\cdots=x_n$ and $x_{n+1}=\cdots=x_{2n}$. □

Lemma S1.3. *Let f be a mapping $\mathbb{N}\to\mathbb{R}_{\geqslant 0}$ such that $f(k)>0$, for every $k\geqslant 2$, and let T be a tree of the form $T_1\star\cdots\star T_k$, with $k\geqslant 3$ (see Fig. 4 in the main text).*

(a) If k is an odd number and if $\delta(T_1)$ is the median of $\{\delta(T_1),\dots,\delta(T_k)\}$, then the tree $T'=T_1\star(T_2\star\cdots\star T_k)$ (cf. Fig. 1) satisfies that $\mathfrak{C}(T')>\mathfrak{C}(T)$.

(b) If k is an even number and if $\delta(T_1),\delta(T_2)$ are the middle values of $\{\delta(T_1),\dots,\delta(T_k)\}$, with $\delta(T_1)\leqslant\delta(T_2)$, then the tree $T''=T_1\star(T_2\star(T_3\star\cdots\star T_k))$ (cf. Fig. 1) satisfies that $\mathfrak{C}(T'')>\mathfrak{C}(T)$.

Proof. Let $t_i=\delta(T_i)$, for every $i=1,\dots,k$.

As to (a), the only nodes in T or T' with different *bal* value in both trees are the roots and the new node v in T'. Therefore,

$$\begin{aligned}
\mathfrak{C}(T')-\mathfrak{C}(T)&=bal_{T'}(v)+bal_{T'}(r)-bal_T(r)\\
&=\mathrm{MDM}(t_2,\dots,t_k)+\frac12\Big|\sum_{i=2}^{k}t_i+f(k-1)-t_1\Big|-\mathrm{MDM}(t_1,\dots,t_k)\geqslant\frac12\Big|\sum_{i=2}^{k}t_i+f(k-1)-t_1\Big|>0,
\end{aligned}$$

where the first inequality is a consequence of Lemma S1.1 and the second inequality is strict because, since t_1 is the median of $\{t_1,\dots,t_k\}$ and $k\geqslant 3$, there is some $i\geqslant 2$ such that $t_i\geqslant t_1$ and hence

$$\Big|\sum_{i=2}^{k}t_i+f(k-1)-t_1\Big|=\sum_{i=2}^{k}t_i+f(k-1)-t_1\geqslant f(k-1)>0$$

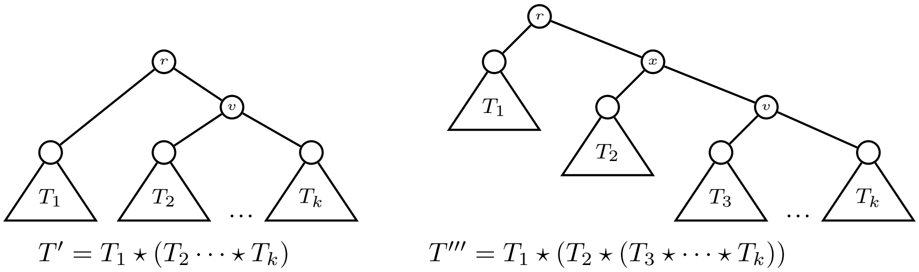

Figure 1: The trees T' and T'' in Lemma S1.3.

As far as (b) goes, the only nodes in T or T'' with different *bal* value in both trees are the roots and the new nodes x, v in T''. Therefore,

$$
\begin{aligned}
\mathfrak{C}(T'') - \mathfrak{C}(T) &= bal_{T''}(v) + bal_{T''}(x) + bal_{T''}(r) - bal_T(r) \\
&= \mathrm{MDM}(t_3, \ldots, t_k) + \frac{1}{2}\Big|\sum_{i=3}^{k} t_i + f(k-2) - t_2\Big| + \frac{1}{2}\Big|\sum_{i=2}^{k} t_i + f(k-2) + f(2) - t_1\Big| - \mathrm{MDM}(t_1, \ldots, t_k) \\
&\geqslant \frac{1}{2}\Big(\Big|\sum_{i=3}^{k} t_i + f(k-2) - t_2\Big| + \Big|\sum_{i=2}^{k} t_i + f(k-2) + f(2) - t_1\Big|\Big) > 0,
\end{aligned}
$$

where the first inequality is a consequence of Lemma S1.2 and the second inequality is strict because, by assumption, $t_1 \leqslant t_2$ and $k \geqslant 3$, and therefore

$$
\Big|\sum_{i=2}^{k} t_i + f(k-2) + f(2) - t_1\Big| = \sum_{i=2}^{k} t_i + f(k-2) + f(2) - t_1 \geqslant f(k-2) + f(2) > 0
$$

□

Lemma S1.4. *Let f be a mapping $\mathbb{N} \to \mathbb{R}_{\geqslant 0}$ such that $f(2) > 0$. Consider the trees T and T' depicted in Fig. 2, where, in both trees, all nodes in the path from r to x are binary, and T' is obtained from T by simply interchanging the subtrees T_l and T_{l-1}. If $\delta(T_l) < \delta(T_{l-1})$ and $\delta(T_l) \leqslant \delta(T_0)$, then $\mathfrak{C}(T') > \mathfrak{C}(T)$.*

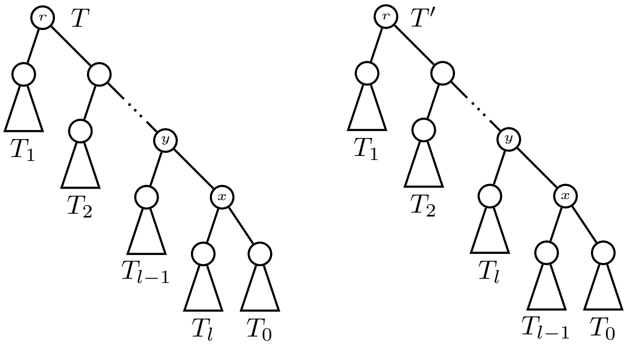

Figure 2: The trees T and T' in Lemma S1.4.

Proof. Let $t_i = \delta(T_i)$, for every $i = 0, \ldots, l$, so that $t_l < t_{l-1}$ and $t_l \leqslant t_0$. Since $\delta(T_y) = \delta(T'_y)$, the only nodes in T or T' with different *bal* value in both trees are x and y, and therefore,

$$
\begin{aligned}
\mathfrak{C}(T') - \mathfrak{C}(T) &= bal_{T'}(x) + bal_{T'}(y) - bal_T(x) - bal_T(y) \\
&= \frac{1}{2}|t_{l-1} - t_0| + \frac{1}{2}|t_{l-1} + t_0 + f(2) - t_l| - \frac{1}{2}|t_l - t_0| - \frac{1}{2}|t_l + t_0 + f(2) - t_{l-1}| = (*)
\end{aligned}
$$

Now we must distinguish two cases:

- If $t_l < t_{l-1} \leqslant t_0$, then

$$\begin{aligned}(*) &= \frac{1}{2}\big((t_0 - t_{l-1}) + (t_{l-1} + t_0 + f(2) - t_l) - (t_0 - t_l) - (t_l + t_0 + f(2) - t_{l-1})\big)\\ &= \frac{1}{2}(t_{l-1} - t_l) > 0\end{aligned}$$

- If $t_l \leqslant t_0 \leqslant t_{l-1}$ and $t_l < t_{l-1}$, then

$$\begin{aligned}(*) &= \frac{1}{2}\big((t_{l-1} - t_0) + (t_{l-1} + t_0 + f(2) - t_l) - (t_0 - t_l) - |t_l + t_0 + f(2) - t_{l-1}|\big)\\ &= \begin{cases} \frac{1}{2}\big(2t_{l-1} - t_0 + f(2) - (t_l + t_0 + f(2) - t_{l-1})\big) = \frac{1}{2}(3t_{l-1} - 2t_0 - t_l) \geqslant \frac{1}{2}(t_{l-1} - t_l) > 0 \\ \qquad (\text{if } t_l + t_0 + f(2) - t_{l-1} \geqslant 0) \\ \frac{1}{2}\big(2t_{l-1} - t_0 + f(2) - (t_{l-1} - t_l - t_0 - f(2))\big) = \frac{1}{2}(t_{l-1} + t_l + 2f(2)) > 0 \\ \qquad (\text{if } t_l + t_0 + f(2) - t_{l-1} \leqslant 0) \end{cases}\end{aligned}$$

and therefore, in all cases, $\mathfrak{C}(T') - \mathfrak{C}(T) > 0$, as we claimed. □

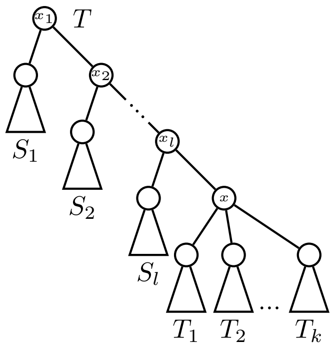

Figure 3: The tree T in Lemma S1.5.

Lemma S1.5. *Let f be a mapping $\mathbb{N} \to \mathbb{R}_{\geqslant 0}$ such that $0 < f(k) < f(k-1) + f(2)$, for every $k \geqslant 3$, and let T be the tree depicted in Fig. 3, where $l \geqslant 1$, x_1 is the root, all nodes in the path from x_1 to x_l are binary, and $k \geqslant 3$. Assume moreover that $\delta(S_1) \leqslant \delta(S_2) \leqslant \cdots \leqslant \delta(S_l)$.*

(a) Assume that k is odd and that $\delta(T_1)$ is the median of $\{\delta(T_1), \dots, \delta(T_k)\}$.

(a.1) If $\delta(S_l) \leqslant \delta(T_1)$, then the tree T' depicted in Fig. 4, obtained by pruning the subtree T_1 and regrafting it in the arc ending in x, satisfies that $\mathfrak{C}(T') > \mathfrak{C}(T)$.

(a.2) If $\delta(S_l) > \delta(T_1)$, then the tree T'' depicted in Fig. 4, obtained by pruning the subtree T_1 and regrafting it in the arc ending in x_l, satisfies that $\mathfrak{C}(T'') > \mathfrak{C}(T)$.

(b) Assume that k is even and that $\delta(T_1), \delta(T_2)$ are the middle values of $\{\delta(T_1), \dots, \delta(T_k)\}$.

(b.1) If $\delta(S_l) \leqslant \delta(T_1 \star T_2)$, then the tree T' depicted in Fig. 5, obtained by pruning the subtrees T_1 and T_2 and then inserting $T_1 \star T_2$ in the arc ending in x, satisfies that $\mathfrak{C}(T') > \mathfrak{C}(T)$.

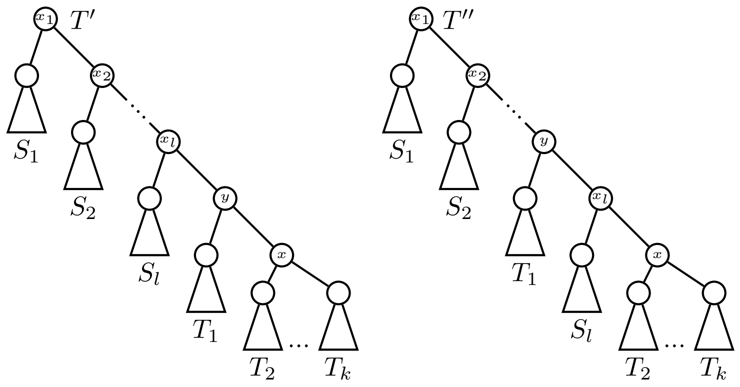

Figure 4: The trees T', T'' in Lemma S1.5.(a).

(b.2) If $\delta(S_l) > \delta(T_1 \star T_2)$, then the tree T'' depicted in Fig. 5, obtained by pruning the subtrees T_1 and T_2 and then inserting $T_1 \star T_2$ in the arc ending in x_l, satisfies that $\mathfrak{C}(T'') > \mathfrak{C}(T)$.

Proof. For every $i = 1, \ldots, l$, let x_i denote the parent of the root of S_i in all trees in the statement. Moreover, for every $i = 1, \ldots, l$, let $s_i = \delta(S_i)$ and, for every $i = 1, \ldots, k$, let $\delta(T_i) = t_i$, and let $t = \sum\limits_{i=1}^{k} t_i$. Recall that we are assuming throughout this proof that $s_1 \leqslant \cdots \leqslant s_l$.

(a) Assume that $k \geqslant 3$ is odd and that $\mathrm{MDM}(t_1, \ldots, t_k) \leqslant \mathrm{MDM}(t_2, \ldots, t_k)$.

As far as assertion (a.1) goes, let us assume that $s_l \leqslant t_1$ and therefore that $s_i \leqslant t$ for every $i = 1, \ldots, l$. In this case, the only nodes in T or T' with different *bal* value in both trees are $x_1, \ldots, x_l$, x and the new node y in T'. Then:

$$
\begin{aligned}
\mathfrak{C}(T') - \mathfrak{C}(T) &= bal_{T'}(x) - bal_T(x) + bal_{T'}(y) + \sum_{i=1}^{l}(bal_{T'}(x_i) - bal_T(x_i)) \\
&= \mathrm{MDM}(t_2, \ldots, t_k) - \mathrm{MDM}(t_1, \ldots, t_k) + \frac{1}{2}\Big|\sum_{i=2}^{k} t_i + f(k-1) - t_1\Big| \\
&\quad + \frac{1}{2}\sum_{i=1}^{l}\Big(\Big|t + \sum_{j=i+1}^{l} s_j + f(k-1) + (l-i+1)f(2) - s_i\Big| - \Big|t + \sum_{j=i+1}^{l} s_j + f(k) + (l-i)f(2) - s_i\Big|\Big) \\
&\geqslant \frac{1}{2}\sum_{i=1}^{l}\Big(\Big|t + \sum_{j=i+1}^{l} s_j + f(k-1) + (l-i+1)f(2) - s_i\Big| - \Big|t + \sum_{j=i+1}^{l} s_j + f(k) + (l-i)f(2) - s_i\Big|\Big) \\
&= \frac{1}{2}\sum_{i=1}^{l}\Bigg(\Big(t + \sum_{j=i+1}^{l} s_j + f(k-1) + (l-i+1)f(2) - s_i\Big) - \Big(t + \sum_{j=i+1}^{l} s_j + f(k) + (l-i)f(2) - s_i\Big)\Bigg) \\
&\quad \text{(because } s_i \leqslant t \text{ for every } i = 1, \ldots, l) \\
&= \frac{l}{2}(f(k-1) + f(2) - f(k)) > 0
\end{aligned}
$$

As far as assertion (a.2) goes, let us assume that $s_l > t_1$. Again, the only nodes in T or T'' with different *bal* value in both trees are $x_1, \ldots, x_l$, x and the new node y. Therefore:

$$\mathfrak{C}(T'') - \mathfrak{C}(T) = bal_{T''}(x) - bal_T(x) + bal_{T''}(y) + bal_{T''}(x_l) - bal_T(x_l) + \sum_{i=1}^{l-1}(bal_{T''}(x_i) - bal_T(x_i))$$

$$= \mathrm{MDM}(t_2, \ldots, t_k) - \mathrm{MDM}(t_1, \ldots, t_k) + \frac{1}{2}\Big|\sum_{i=2}^{k} t_i + s_l + f(k-1) + f(2) - t_1\Big|$$

$$+\frac{1}{2}\Big|\sum_{i=2}^{k} t_i + f(k-1) - s_l\Big| - \frac{1}{2}|t + f(k) - s_l|$$

$$+\frac{1}{2}\sum_{i=1}^{l-1}\Big(\Big|t + \sum_{j=i+1}^{l} s_j + f(k-1) + (l-i+1)f(2) - s_i\Big| - \Big|t + \sum_{j=i+1}^{l} s_j + f(k) + (l-i)f(2) - s_i\Big|\Big)$$

$$\geqslant \frac{1}{2}\Big(\Big|\sum_{i=2}^{k} t_i + s_l + f(k-1) + f(2) - t_1\Big| - |t + f(k) - s_l|\Big)$$

$$+\frac{1}{2}\sum_{i=1}^{l-1}\Big(\Big|t + \sum_{j=i+1}^{l} s_j + f(k-1) + (l-i+1)f(2) - s_i\Big| - \Big|t + \sum_{j=i+1}^{l} s_j + f(k) + (l-i)f(2) - s_i\Big|\Big)$$

$$= \frac{1}{2}\Big(\sum_{i=2}^{k} t_i + s_l + f(k-1) + f(2) - t_1 - |t + f(k) - s_l|\Big)$$

$$+\frac{1}{2}\sum_{i=1}^{l-1}\Big(\Big(t + \sum_{j=i+1}^{l} s_j + f(k-1) + (l-i+1)f(2) - s_i\Big) - \Big(t + \sum_{j=i+1}^{l} s_j + f(k) + (l-i)f(2) - s_i\Big)\Big)$$

(because $s_i \leqslant s_l$, for every $i = 1, \ldots, l$, and $s_l > t_1$)

$$= \frac{1}{2}\Big(\sum_{i=2}^{k} t_i + s_l + f(k-1) + f(2) - t_1 - |t + f(k) - s_l|\Big) + \frac{l-1}{2}(f(k-1) + f(2) - f(k))$$

$$\geqslant \frac{1}{2}\Big(\sum_{i=2}^{k} t_i + s_l + f(k-1) + f(2) - t_1 - |t + f(k) - s_l|\Big)$$

$$= \begin{cases} \frac{1}{2}(2(s_l - t_1) + f(k-1) + f(2) - f(k)) > 0 & (\text{if } s_l \leqslant t + f(k)) \\ \frac{1}{2}\Big(2\sum_{i=2}^{k} t_i + f(k-1) + f(2) + f(k)\Big) > 0 & (\text{if } s_l \geqslant t + f(k)) \end{cases}$$

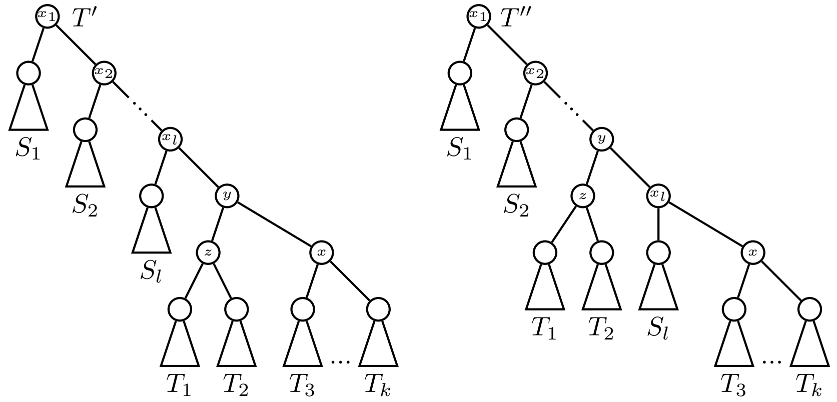

Figure 5: The trees in Lemma S1.5.(b).

(b) Assume now that $k \geqslant 3$ is even, and hence $k \geqslant 4$, and that $\mathrm{MDM}(t_1, \ldots, t_k) \leqslant \mathrm{MDM}(t_3, \ldots, t_k)$.

As far as assertion (b.1) goes, let us assume that $s_l \leqslant t_1 + t_2 + f(2) \leqslant t + f(2)$. The only nodes in T or T' with different *bal* value in both trees are $x_1, \ldots, x_l$, x and the new nodes y and z in T'. Therefore:

$$
\begin{aligned}
\mathfrak{C}(T') - \mathfrak{C}(T) &= bal_{T'}(x) - bal_T(x) + bal_{T'}(z) + bal_{T'}(y) + bal_{T'}(x_l) - bal_T(x_l) + \sum_{i=1}^{l-1}(bal_{T'}(x_i) - bal_T(x_i)) \\
&= \mathrm{MDM}(t_3, \ldots, t_k) - \mathrm{MDM}(t_1, \ldots, t_k) + \frac{1}{2}|t_2 - t_1| + \frac{1}{2}\Big|\sum_{i=3}^{k} t_i + f(k-2) - (t_2 + t_1 + f(2))\Big| \\
&\quad + \frac{1}{2}|t + f(k-2) + 2f(2) - s_l| - \frac{1}{2}|t + f(k) - s_l| \\
&\quad + \frac{1}{2}\sum_{i=1}^{l-1}\Big(\Big|t + \sum_{j=i+1}^{l} s_j + f(k-2) + (l-i+2)f(2) - s_i\Big| - \Big|t + \sum_{j=i+1}^{l} s_j + f(k) + (l-i)f(2) - s_i\Big|\Big) \\
&\geqslant \frac{1}{2}\big(t + f(k-2) + 2f(2) - s_l - |t + f(k) - s_l|\big) \\
&\quad + \frac{1}{2}\sum_{i=1}^{l-1}\Big(t + \sum_{j=i+1}^{l} s_j + f(k-2) + (l-i+2)f(2) - s_i - \Big(t + \sum_{j=i+1}^{l} s_j + f(k) + (l-i)f(2) - s_i\Big)\Big) \\
&\quad \text{(because } s_l \leqslant t + f(2) \text{ and } s_i \leqslant s_l, \text{ for every } i = 1, \ldots, l-1) \\
&= \frac{1}{2}\big(t + f(k-2) + 2f(2) - s_l - |t + f(k) - s_l|\big) + \frac{l-1}{2}(f(k-2) + 2f(2) - f(k)) \\
&\geqslant \frac{1}{2}\big(t + f(k-2) + 2f(2) - s_l - |t + f(k) - s_l|\big) + \frac{l-1}{2}(f(k-1) + f(2) - f(k)) \\
&\geqslant \frac{1}{2}\big(t + f(k-2) + 2f(2) - s_l - |t + f(k) - s_l|\big) \\
&= \begin{cases} \frac{1}{2}(f(k-2) + 2f(2) - f(k)) > 0 & \text{if } s_l \leqslant t + f(k) \\ \frac{1}{2}(2(t + f(2) - s_l) + f(k-2) + f(k)) > 0 & \text{if } s_l \geqslant t + f(k) \end{cases}
\end{aligned}
$$

As far as assertion (b.2) goes, let us assume now that $s_l > t_1 + t_2 + f(2)$. Again, the only nodes in T or T'' with different *bal* value in both trees are $x_1, \ldots, x_l$, x and the new nodes y, z. Therefore:

$$
\begin{aligned}
\mathfrak{C}(T'') - \mathfrak{C}(T) &= bal_{T''}(x) - bal_T(x) + bal_{T''}(z) + bal_{T''}(x_l) - bal_T(x_l) + bal_{T''}(y) + \sum_{i=1}^{l-1}(bal_{T''}(x_i) - bal_T(x_i)) \\
&= \mathrm{MDM}(t_3, \ldots, t_k) - \mathrm{MDM}(t_1, \ldots, t_k) + \frac{1}{2}|t_2 - t_1| + \frac{1}{2}\Big|\sum_{i=3}^{k} t_i + f(k-2) - s_l\Big| \\
&\quad - \frac{1}{2}|t + f(k) - s_l| + \frac{1}{2}\Big|\sum_{i=3}^{k} t_i + s_l + f(k-2) + f(2) - (t_1 + t_2 + f(2))\Big| \\
&\quad + \frac{1}{2}\sum_{i=1}^{l-1}\Big(\Big|t + \sum_{j=i+1}^{l} s_j + f(k-2) + (l-i+2)f(2) - s_i\Big| - \Big|t + \sum_{j=i+1}^{l} s_j + f(k) + (l-i)f(2) - s_i\Big|\Big) \\
&\geqslant \frac{1}{2}\Big(\sum_{i=3}^{k} t_i + s_l + f(k-2) + f(2) - (t_1 + t_2 + f(2)) - |t + f(k) - s_l|\Big) \\
&\quad + \frac{1}{2}\sum_{i=1}^{l-1}\Big(t + \sum_{j=i+1}^{l} s_j + f(k-2) + (l-i+2)f(2) - s_i - \Big(t + \sum_{j=i+1}^{l} s_j + f(k) + (l-i)f(2) - s_i\Big)\Big)
\end{aligned}
$$

$$
\begin{aligned}
&= \frac{1}{2}\Big(\sum_{i=3}^{k} t_i + s_l + f(k-2) - (t_1+t_2) - |t+f(k)-s_l|\Big) + \frac{l-1}{2}(f(k-2)+2f(2)-f(k))\\
&\geqslant \frac{1}{2}\Big(\sum_{i=3}^{k} t_i + s_l + f(k-2) - (t_1+t_2) - |t+f(k)-s_l|\Big) + \frac{l-1}{2}(f(k-1)+f(2)-f(k))\\
&\geqslant \frac{1}{2}\Big(\sum_{i=3}^{k} t_i + s_l + f(k-2) - (t_1+t_2) - |t+f(k)-s_l|\Big)\\
&= \begin{cases} \frac{1}{2}\big(2s_l - 2(t_1+t_2) + f(k-2) - f(k)\big) > \frac{1}{2}(2f(2)+f(k-2)-f(k)) > 0 & \text{if } s_l \leqslant t+f(k)\\ \frac{1}{2}\Big(2\sum_{i=3}^{k} t_i + f(k-2) + f(k)\Big) > 0 & \text{if } s_l \geqslant t+f(k) \end{cases}
\end{aligned}
$$

□

Corollary S1.6. *For every non-binary tree $T \in \mathcal{T}_n^*$, there always exists a binary tree $T' \in \mathcal{T}_n^*$ such that $\mathfrak{C}(T') > \mathfrak{C}(T)$.*

Proof. We shall prove by complete induction on the sum S of the degrees of the non-binary internal nodes in a tree $T \in \mathcal{T}_n^*$ that there always exists a binary tree $T' \in \mathcal{T}_n^*$ such that $\mathfrak{C}(T') \geqslant \mathfrak{C}(T)$. Moreover, it will be clear from the proof that if T isn't binary, then T' can be chosen so that this inequality is strict.

The assertion to be proved by induction is obviously true if $S = 0$ (which means that T is binary), so assume that $S > 0$. Let x be an internal non-binary node of T such that all nodes in the path from the root r to x, except x itself, are binary.

If x is the root, then we apply Lemma S1.3 and we obtain a tree T_0 with smaller S and larger $\mathfrak{C}$. Then, by induction, there exists a binary tree T' such that $\mathfrak{C}(T) < \mathfrak{C}(T_0) \leqslant \mathfrak{C}(T')$.

If x is not the root r, let $r, x_2, \dots, x_l, x$ be the path from the root to x: all these nodes except x are binary. By Lemma S1.4, if we rearrange the subtrees rooted at the children of the nodes $r, x_2, \dots, x_l$ in increasing order of their δ-sizes, the $\mathfrak{C}$ value of the resulting tree increases without modifying the value of S; let $\widehat{T}$ be the tree obtained in this way. Next, by Lemma S1.5, in $\widehat{T}$ we can either prune a subtree T_1 rooted at one child of x and regraft it to an arc in the path from r to x (adding a new binary node to the tree), or we can prune two subtrees T_1, T_2 rooted at two children of x and regraft their star $T_1 \star T_2$ to an arc in the path from r to x (adding two new binary nodes to the tree), in both cases in such a way that the resulting tree T_0 has a larger $\mathfrak{C}$ and a smaller S. Then, by induction, there exists a binary tree T' such that $\mathfrak{C}(T) \leqslant \mathfrak{C}(\widehat{T}) < \mathfrak{C}(T_0) \leqslant \mathfrak{C}(T')$.

This finishes the proof by induction. □

Therefore, the maximum $\mathfrak{C}$ value on $\mathcal{T}_n^*$ is reached at a binary tree, where, by Proposition 6 in the main text, it is equal to $(f(0)+f(2))/2$ times the Colless index, with $f(0)+f(2) \geqslant f(2) > 0$. Then, since, by Lemma 1 in the main text, the maximum Colless index of a binary tree with n leaves is reached exactly at the comb K_n, the same is true for $\mathfrak{C}$. So, the maximum value of $\mathfrak{C}$ on $\mathcal{T}_n^*$ is reached exactly at K_n, and it is

$$\mathfrak{C}(K_n) = \frac{f(0)+f(2)}{2} C(K_n) = \frac{f(0)+f(2)}{4}(n-1)(n-2).$$

B Proof of the thesis of Theorem 18 for $\mathfrak{C}_{sd,f}$

The proof of Theorem 18 for $D = sd$, the sample standard deviation, is very similar to the one provided for $D = \text{MDM}$ in the previous section, but simpler, because Lemmas S1.1 and S1.2 are replaced by Lemma S1.7 below, which guarantees that it is always enough to remove a suitable element in a non-constant numeric vector of length at least 3, in order to increase its variance. To simplify the notations, we shall denote in this section δ_f and $\mathfrak{C}_{sd,f}$ by δ and $\mathfrak{C}$, respectively, and we shall denote $bal_{sd,f}$ on a tree T by bal_T or simply by bal when it is not necessary to specify the tree.

Lemma S1.7. *For every $(x_1,\dots,x_n)\in\mathbb{R}^n$ with $n\geqslant 3$, if x_i is the value in the set $\{x_1,\dots,x_n\}$ closest to its mean, then*

$$\mathrm{var}(x_1,\dots,x_n)\leqslant \mathrm{var}(x_1,\dots,x_{i-1},x_{i+1},\dots,x_n),$$

and thus, taking positive square roots,

$$sd(x_1,\dots,x_n)\leqslant sd(x_1,\dots,x_{i-1},x_{i+1},\dots,x_n).$$

Moreover, these inequalities are strict unless either $x_1=\cdots=x_n$ or n is even and $\{x_1,\dots,x_n\}$ consists of $n/2$ copies of two different elements.

Proof. Let $\overline{x}=(x_1+\cdots+x_n)/n$ and, after rearranging $x_1,\dots,x_n$ if necessary, assume that $(x_n-\overline{x})^2\leqslant(x_i-\overline{x})^2$, for every $i=1,\dots,n-1$. We shall prove that

$$\mathrm{var}(x_1,\dots,x_n)\leqslant \mathrm{var}(x_1,\dots,x_{n-1}).$$

Indeed, let $\overline{x}'=(x_1+\cdots+x_{n-1})/(n-1)$. Then

$$\mathrm{var}(x_1,\dots,x_{n-1})\geqslant \mathrm{var}(x_1,\dots,x_n)\Longleftrightarrow (n-1)\sum_{i=1}^{n-1}(x_i-\overline{x}')^2\geqslant (n-2)\sum_{i=1}^{n}\Big(x_i-\overline{x})^2$$

Now

$$\begin{aligned}
(n-1)\sum_{i=1}^{n-1}(x_i-\overline{x}')^2 &= (n-1)\sum_{i=1}^{n-1}\Big(x_i-\overline{x}+\frac{1}{n-1}(x_n-\overline{x})\Big)^2\\
&=(n-1)\sum_{i=1}^{n-1}\Big((x_i-\overline{x})^2+\frac{2}{n-1}(x_i-\overline{x})(x_n-\overline{x})+\frac{1}{(n-1)^2}(x_n-\overline{x})^2\Big)\\
&=(n-1)\sum_{i=1}^{n-1}(x_i-\overline{x})^2+2(x_n-\overline{x})\sum_{i=1}^{n-1}(x_i-\overline{x})+(x_n-\overline{x})^2\\
&=(n-1)\sum_{i=1}^{n-1}(x_i-\overline{x})^2+2(x_n-\overline{x})(\overline{x}-x_n)+(x_n-\overline{x})^2\\
&=(n-1)\sum_{i=1}^{n-1}(x_i-\overline{x})^2-(x_n-\overline{x})^2\\
&=(n-2)\sum_{i=1}^{n-1}(x_i-\overline{x})^2+\sum_{i=1}^{n-1}(x_i-\overline{x})^2-(x_n-\overline{x})^2\\
&\geqslant (n-2)\sum_{i=1}^{n-1}(x_i-\overline{x})^2+(n-1)(x_n-\overline{x})^2-(x_n-\overline{x})^2=(n-2)\sum_{i=1}^{n}(x_i-\overline{x})^2
\end{aligned}$$

as we wanted to prove. This inequality is an equality if, and only if, $(x_i-\overline{x})^2=(x_n-\overline{x})^2$ for every $i=1,\dots,n-1$, and it is easy to check that this condition holds exactly when either $x_1=\cdots=x_n$ or n is even and $\{x_1,\dots,x_n\}$ consists of $n/2$ copies of two different elements. □

We prove now a series of lemmas that play in this proof the same role as Lemmas S1.3 to S1.5 in the last section.

Lemma S1.8. *Let f be a mapping $\mathbb{N}\to\mathbb{R}_{\geqslant 0}$ such that $f(k)>0$, for every $k\geqslant 2$, and let T be a tree of the form $T_1\star\cdots\star T_k$, with $k\geqslant 3$. If $\delta(T_1)$ is the value in the set $\{\delta(T_1),\dots,\delta(T_k)\}$ closest to its mean, then taking the tree $T'=T_1\star(T_2\star\cdots\star T_k)$ depicted in the left-hand side of Fig. 1, we obtain that $\mathfrak{C}(T')>\mathfrak{C}(T)$.*

Proof. Let $t_i=\delta(T_i)$, for every $i=1,\dots,k$. The only nodes in T or T' with different *bal* value in both trees are the roots and the new node v in T'. Therefore,

$$\begin{aligned}
\mathfrak{C}(T')-\mathfrak{C}(T)&=bal_{T'}(r)+bal_{T'}(v)-bal_T(r)\\
&=\frac{1}{\sqrt{2}}\Big|\sum_{i=2}^{k}t_i+f(k-1)-t_1\Big|+sd(t_2,\dots,t_k)-sd(t_1,\dots,t_k)\geqslant\frac{1}{\sqrt{2}}\Big|\sum_{i=2}^{k}t_i+f(k-1)-t_1\Big|\geqslant 0
\end{aligned}$$

Now, the first inequality is strict unless either $t_1 = t_2 = \cdots = t_k$ or (up to reordering the trees $T_2, \ldots, T_k$) $k = 2m \geqslant 4$, $t_1 = \cdots = t_m$ and $t_{m+1} = \cdots = t_k$, and in both cases

$$\Big|\sum_{i=2}^{k} t_i + f(k-1) - t_1\Big| = \sum_{i=3}^{k} t_i + f(k-1) > 0$$

Therefore, we always have that $\mathfrak{C}(T') - \mathfrak{C}(T) > 0$. □

The proof of the following lemma is the same as that of Lemma S1.4 (up to replacing the fractions 1/2 by $1/\sqrt{2}$), and we shall not repeat it here.

Lemma S1.9. *Let f be a mapping $\mathbb{N} \to \mathbb{R}_{\geqslant 0}$ such that $f(2) > 0$. Consider the trees T and T' depicted in Fig. 2, where T' is obtained from T by simply interchanging the subtrees T_l and T_{l-1}. If $\delta(T_l) < \delta(T_{l-1})$ and $\delta(T_l) \leqslant \delta(T_0)$, then $\mathfrak{C}(T') > \mathfrak{C}(T)$.* □

Lemma S1.10. *Let f be a mapping $\mathbb{N} \to \mathbb{R}_{\geqslant 0}$ such that $0 < f(k) < f(k-1) + f(2)$, for every $k \geqslant 3$, and let T be the tree depicted in Fig. 3, where $l \geqslant 1$, x_1 is the root, all nodes in the path from x_1 to x_l are binary, and $k \geqslant 3$. Assume moreover that $\delta(S_1) \leqslant \delta(S_2) \leqslant \cdots \leqslant \delta(S_l)$ and that $\delta(T_1)$ is the value in the set $\{\delta(T_1), \ldots, \delta(T_k)\}$ closest to its mean.*

(a) If $\delta(S_l) \leqslant \delta(T_1)$, then the tree T' depicted in Fig. 4, obtained by pruning the subtree T_1 and regrafting it in the arc ending in x, is such that $\mathfrak{C}(T') > \mathfrak{C}(T)$.

(b) If $\delta(S_l) > \delta(T_1)$, then the tree T'' depicted in Fig. 4, obtained by pruning the subtree T_1 and regrafting it in the arc ending in x_l, is such that $\mathfrak{C}(T'') > \mathfrak{C}(T)$.

Proof. For every $i = 1, \ldots, l$, let $s_i = \delta(S_i)$ and, for every $i = 1, \ldots, k$, $\delta(T_i) = t_i$. Let, moreover, $t = t_1 + \cdots + t_k$. So, we are assuming that $s_1 \leqslant \cdots \leqslant s_l$ and that $sd(t_1, \ldots, t_k) \leqslant sd(t_2, \ldots, t_k)$. Moreover, for every $i = 1, \ldots, l$, we shall call x_i the parent of the root of S_i in all three trees T, T', T''.

As far as assertion (a) goes, the only nodes in T or T' with different *bal* value in both trees are $x_1, \ldots, x_l$, x and the new node y. Therefore:

$$\begin{aligned}
\mathfrak{C}(T') - \mathfrak{C}(T) &= bal_{T'}(x) - bal_T(x) + bal_{T'}(y) + \sum_{i=1}^{l} \big(bal_{T'}(x_i) - bal_T(x_i)\big) \\
&= sd(t_2, \ldots, t_k) - sd(t_1, \ldots, t_k) + \frac{1}{\sqrt{2}}\Big|\sum_{i=2}^{k} t_i + f(k-1) - t_1\Big| \\
&\quad + \sum_{i=1}^{l} \frac{1}{\sqrt{2}}\Big(\Big|t + \sum_{j=i+1}^{l} s_j + f(k-1) + (l-i+1)f(2) - s_i\Big| - \Big|t + \sum_{j=i+1}^{l} s_j + f(k) + (l-i)f(2) - s_i\Big|\Big) \\
&\geqslant \frac{1}{\sqrt{2}} \sum_{i=1}^{l} \Big(\Big|t + \sum_{j=i+1}^{l} s_j + f(k-1) + (l-i+1)f(2) - s_i\Big| - \Big|t + \sum_{j=i+1}^{l} s_j + f(k) + (l-i)f(2) - s_i\Big|\Big) \\
&= \frac{l}{\sqrt{2}}(f(k-1) + f(2) - f(k)) > 0
\end{aligned}$$

where, as in the proof of Lemma S1.5.(a1), the last equality is a consequence of the fact that, for every $i = 1, \ldots, l$, $s_i \leqslant s_l \leqslant t_1 \leqslant t$.

Let us prove now assertion (b). Again, the only nodes in T or T'' with different *bal* value in both trees are

$x_1, \dots, x_l$, x and the new node y. Therefore,

$$
\begin{aligned}
\mathfrak{C}(T') - \mathfrak{C}(T) &= bal_{T''}(x) - bal_T(x) + bal_{T''}(x_l) + bal_{T''}(y) - bal_T(x_l) + \sum_{i=1}^{l-1}(bal_{T''}(x_i) - bal_T(x_i))\\
&= sd(t_2, \dots, t_k) - sd(t_1, \dots, t_k) + \frac{1}{\sqrt{2}}\Big|\sum_{i=2}^{k} t_i + f(k-1) - s_l\Big|\\
&\quad + \frac{1}{\sqrt{2}}\Big|\sum_{i=2}^{k} t_i + s_l + f(k-1) + f(2) - t_1\Big| - \frac{1}{\sqrt{2}}|t + f(k) - s_l|\\
&\quad + \frac{1}{\sqrt{2}}\sum_{i=1}^{l-1}\Big(\Big|t + \sum_{j=i+1}^{l} s_j + f(k-1) + (l-i+1)f(2) - s_i\Big| - \Big|t + \sum_{j=i+1}^{l} + f(k) + (l-i)f(2) - s_i\Big|\Big)\\
&\geqslant \frac{1}{\sqrt{2}}\Big(\Big|\sum_{i=2}^{k} t_i + s_l + f(k-1) + f(2) - t_1\Big| - |t + f(k) - s_l|\Big)\\
&\quad + \frac{1}{\sqrt{2}}\sum_{i=1}^{l-1}\Big(\Big|t + \sum_{j=i+1}^{l} s_j + f(k-1) + (l-i+1)f(2) - s_i\Big| - \Big|t + \sum_{j=i+1}^{l} s_j + f(k) + (l-i)f(2) - s_i\Big|\Big) > 0
\end{aligned}
$$

where the last strict inequality is derived as in the proof of Lemma S1.5.(a2). □

Then, using Lemmas S1.8 to S1.10 and arguing as in the proof of Corollary S1.6, we deduce that, for every non-binary tree $T \in \mathcal{T}_n^*$, there always exists a binary tree $T' \in \mathcal{T}_n^*$ such that $\mathfrak{C}(T') > \mathfrak{C}(T)$. Starting from this fact, the same argument that completes the proof of Theorem 18 for $\mathfrak{C}_{\mathrm{MDM},f}$ also completes it for $\mathfrak{C}_{sd,f}$.

C Proof of the thesis of Theorem 18 for $\mathfrak{C}_{\mathrm{var},f}$

The proof is similar to those described in the previous two sections, using Lemma S1.7 and proving a series of lemmas that show how to increase the Colless-like index of a non-binary tree by making it "more binary." To simplify the notations, in this section, we shall denote δ_f and $\mathfrak{C}_{\mathrm{var},f}$ by simply δ and $\mathfrak{C}$, respectively, and we shall denote $bal_{\mathrm{var},f}$ on a tree T by bal_T or simply by bal when it is not necessary to specify the tree.

Lemma S1.11. *Let f be a mapping $\mathbb{N} \to \mathbb{R}_{\geqslant 0}$ such that $f(k) > 0$, for every $k \geqslant 2$, and let T be a tree of the form $T_1 \star \cdots \star T_k$, with $k \geqslant 3$. If $\delta(T_1)$ is the value in the set $\{\delta(T_1), \dots, \delta(T_k)\}$ closest to its mean, then taking the tree $T' = T_1 \star (T_2 \star \cdots \star T_k)$ depicted in the left-hand side of Fig. 1, we obtain that $\mathfrak{C}(T') > \mathfrak{C}(T)$.*

Proof. Let $t_i = \delta(T_i)$, for every $i = 1, \dots, k$. The only nodes in T or T' with different bal value in both trees are the roots and the new node v in T'. Therefore,

$$
\begin{aligned}
\mathfrak{C}(T') - \mathfrak{C}(T) &= bal_{T'}(v) + bal_{T'}(r) - bal_T(r)\\
&= \mathrm{var}(t_2, \dots, t_k) + \frac{1}{2}\Big(\sum_{i=2}^{k} t_i + f(k-1) - t_1\Big)^2 - \mathrm{var}(t_1, \dots, t_k) \geqslant \frac{1}{2}\Big(\sum_{i=2}^{k} t_i + f(k-1) - t_1\Big)^2 \geqslant 0
\end{aligned}
$$

Now, the first inequality is strict unless either $t_1 = t_2 = \cdots = t_k$ or (up to reordering the trees $T_2, \dots, T_k$) $k = 2m \geqslant 4$, $t_1 = \cdots = t_m$ and $t_{m+1} = \cdots = t_k$, and in both cases the last inequality is strict (cf. the proof of Lemma S1.8). □

Lemma S1.12. *Let f be a mapping $\mathbb{N} \to \mathbb{R}_{\geqslant 0}$ such that $f(2) > 0$. Consider the trees T and T' depicted in Fig. 2, where, in both trees, the nodes in the path connecting the root r with x are binary, and T' is obtained from T by simply interchanging the subtrees T_l and T_{l-1}. If $\delta(T_l) < \delta(T_{l-1})$, then $\mathfrak{C}(T') > \mathfrak{C}(T)$.*

Proof. Let $t_i = \delta(T_i)$, for every $i = 0, \dots, l$, so that $t_l < t_{l-1}$. The only nodes in T or T' with different *bal* value in both trees are x and y, and therefore,

$$\begin{aligned}\mathfrak{C}(T') - \mathfrak{C}(T) &= bal_{T'}(x) + bal_{T'}(y) - bal_T(x) - bal_T(y) \\ &= \frac{1}{2}(t_{l-1} - t_0)^2 + \frac{1}{2}(t_{l-1} + t_0 + f(2) - t_l)^2 - \frac{1}{2}(t_l - t_0)^2 - \frac{1}{2}(t_l + t_0 + f(2) - t_{l-1})^2 \\ &= \frac{1}{2}(t_{l-1} - t_l)(t_{l-1} + t_l + 2t_0 + 4f(2)) > 0\end{aligned}$$

□

Lemma S1.13. *Let f be a mapping $\mathbb{N} \to \mathbb{R}_{\geqslant 0}$ such that $0 < f(k) < f(k-1) + f(2)$, for every $k \geqslant 3$, and let T be the tree depicted in Fig. 3, where $l \geqslant 1$, x_1 is the root, all nodes in the path from x_1 to x_l are binary, and $k \geqslant 3$. Assume moreover that $\delta(S_1) \leqslant \delta(S_2) \leqslant \cdots \leqslant \delta(S_l)$ and that $\delta(T_1)$ is the value in the set $\{\delta(T_1), \dots, \delta(T_k)\}$ closest to its mean. Then:*

(a) If $\delta(S_l) \leqslant \delta(T_1)$, then the tree T' depicted in Fig. 4, obtained by pruning the subtree T_1 and regrafting it in the arc ending in x, is such that $\mathfrak{C}(T') > \mathfrak{C}(T)$.

(b) If $\delta(S_l) > \delta(T_1)$, then the tree T'' depicted in Fig. 4, obtained by pruning the subtree T_1 and regrafting it in the arc ending in x_l, is such that $\mathfrak{C}(T'') > \mathfrak{C}(T)$.

Proof. For every $i = 1, \dots, l$, let $s_i = \delta(S_i)$ and, for every $i = 1, \dots, k$, $\delta(T_i) = t_i$, and let $t = t_1 + \cdots + t_k$. We are assuming that $s_1 \leqslant \cdots \leqslant s_l$ and that $\mathrm{var}(t_1, \dots, t_k) \leqslant \mathrm{var}(t_2, \dots, t_k)$. Moreover, for every $i = 1, \dots, l$, we shall call x_i the parent of the root of S_i in all three trees T, T', T''.

As far as assertion (a) goes, the only nodes in T or T' with different *bal* value in both trees are $x_1, \dots, x_l$, x and the new node y. Therefore,

$$\begin{aligned}\mathfrak{C}(T') - \mathfrak{C}(T) &= bal_{T'}(x) - bal_T(x) + bal_{T'}(y) + \sum_{i=1}^{l}(bal_{T'}(x_i) - bal_T(x_i)) \\ &= \mathrm{var}(t_2, \dots, t_k) - \mathrm{var}(t_1, \dots, t_k) + \frac{1}{2}\Big(\sum_{i=2}^{k} t_i + f(k-1) - t_1\Big)^2 \\ &\quad + \sum_{i=1}^{l}\Big(\frac{1}{2}\Big(t + \sum_{j=i+1}^{l} s_j + f(k-1) + (l-i+1)f(2) - s_i\Big)^2 - \frac{1}{2}\Big(t + \sum_{j=i+1}^{l} s_j + f(k) + (l-i)f(2) - s_i\Big)^2\Big) \\ &\geqslant \frac{1}{2}\sum_{i=1}^{l}\Big(\Big(t + \sum_{j=i+1}^{l} s_j + f(k-1) + (l-i+1)f(2) - s_i\Big)^2 - \Big(t + \sum_{j=i+1}^{l} s_j + f(k) + (l-i)f(2) - s_i\Big)^2\Big) \\ &= \frac{1}{2}\big(f(k-1) + f(2) - f(k)\big)\sum_{i=1}^{l}\Big(2(t + \sum_{j=i+1}^{l} s_j - s_i) + f(k-1) + f(k) + (2(l-i)+1)f(2)\Big) > 0,\end{aligned}$$

where this last expression is > 0 because $f(k-1) + f(2) - f(k) > 0$ and, for every $i = 1, \dots, l$, $s_i \leqslant s_l \leqslant t_1 \leqslant t$.

Let us prove now assertion (b). Again, the only nodes in T or T'' with different *bal* value in both trees are

$x_1, \ldots, x_l$, x and the new node y. Therefore,

$$
\begin{aligned}
\mathfrak{C}(T') - \mathfrak{C}(T) &= bal_{T''}(x) - bal_T(x) + bal_{T''}(y) + bal_{T''}(x_l) - bal_T(x_l) + \sum_{i=1}^{l-1}(bal_{T''}(x_i) - bal_T(x_i)) \\
&= \mathrm{var}(t_2, \ldots, t_k) - \mathrm{var}(t_1, \ldots, t_k) + \frac{1}{2}\Big(\sum_{i=2}^{k} t_i + s_l + f(k-1) + f(2) - t_1\Big)^2 \\
&\quad + \frac{1}{2}\Big(\sum_{i=2}^{k} t_i + f(k-1) - s_l\Big)^2 - \frac{1}{2}(t + f(k) - s_l)^2 \\
&\quad + \frac{1}{2}\sum_{i=1}^{l-1}\Big(\Big(t + \sum_{j=i+1}^{l} s_j + f(k-1) + (l-i+1)f(2) - s_i\Big)^2 - \Big(t + \sum_{j=i+1}^{l} + f(k) + (l-i)f(2) - s_i\Big)^2\Big) \\
&\geqslant \frac{1}{2}\Big(\Big(\sum_{i=2}^{k} t_i + s_l + f(k-1) + f(2) - t_1\Big)^2 - (t + f(k) - s_l)^2\Big) \\
&\quad + \frac{1}{2}\sum_{i=1}^{l-1}\Big(\Big(t + \sum_{j=i+1}^{l} s_j + f(k-1) + (l-i+1)f(2) - s_i\Big)^2 - \Big(t + \sum_{j=i+1}^{l} s_j + f(k) + (l-i)f(2) - s_i\Big)^2\Big) \\
&= \frac{1}{2}\Big(2\sum_{i=2}^{k} t_i + f(k-1) + f(2) + f(k)\Big)(f(k-1) + f(2) - f(k) + 2(s_l - t_1)) \\
&\quad + \frac{1}{2}(f(k-1) + f(2) - f(k))\sum_{i=1}^{l-1}\Big(2\Big(t + \sum_{j=i+1}^{l} s_j - s_i\Big) + f(k-1) + f(k) + (2(l-i)+1)f(2)\Big) > 0
\end{aligned}
$$

where this last expression is > 0 because $f(k-1) + f(2) - f(k) > 0$, $s_l > t_1$ and, for every $i = 1, \ldots, l-1$, $s_i \leqslant s_l$. $\square$

Then, using Lemmas S1.11 to S1.13 and arguing as in the proof of Corollary S1.6, it can be proved that, for every non-binary tree $T \in \mathcal{T}_n^*$, there always exists a binary tree $T' \in \mathcal{T}_n^*$ such that $\mathfrak{C}(T') > \mathfrak{C}(T)$. Therefore, the maximum $\mathfrak{C}$ value is reached at some binary tree. Since, for binary trees T, $\mathfrak{C}(T) = \frac{(f(0)+f(2))^2}{2} \cdot C^{(2)}(T)$ (see Proposition 7 in the main text), and $f(0) + f(2) > 0$, it remains to prove that the binary tree in $\mathcal{T}_n^*$ with maximum $C^{(2)}$ is exactly the comb. The proof of this fact follows closely that of Lemma 1 in the main text.

Corollary S1.14. *For every binary tree $T \in \mathcal{T}_n^*$, if $T \neq K_n$, then $C^{(2)}(K_n) > C^{(2)}(T)$.*

Proof. Using the argument of the proof of Lemma 1 in the main text, it is enough to prove that if T and T' are the trees depicted in Fig. 6 in the main text, then, under the assumptions therein, $C^{(2)}(T') > C^{(2)}(T)$. And, indeed (using the notations therein),

$$
\begin{aligned}
C^{(2)}(T') - C^{(2)}(T) &= (t_3 + t_4 - t_2)^2 + (t_3 + t_4 + t_2 - t_1)^2 - (t_2 - t_1)^2 - (t_3 + t_4 - t_2 - t_1)^2 \\
&= (t_3 + t_4 - t_1)(t_3 + t_4 + t_1 + 2t_2) > 0
\end{aligned}
$$

where the last inequality holds because, by assumption, $t_1, t_2, t_3, t_4 > 0$ and $t_1 + t_2 \leqslant t_3 + t_4$. $\square$

Now, it is straightforward to check that

$$
C^{(2)}(K_n) = \sum_{k=1}^{n-2} k^2 = \frac{1}{6}(n-1)(n-2)(2n-3),
$$

from where we obtain

$$
\mathfrak{C}(K_n) = \frac{(f(0) + f(2))^2}{2} \cdot C^{(2)}(K_n) = \frac{(f(0) + f(2))^2}{12}(n-1)(n-2)(2n-3),
$$

as we claimed in the statement.

D Proof of the thesis of Theorem 19 for $\mathfrak{C}_{\mathrm{MDM},e^n}$

To simplify the notations, we shall denote in this section δ_{e^n} and $\mathfrak{C}_{\mathrm{MDM},e^n}$ by δ and $\mathfrak{C}$, respectively, and we shall denote $bal_{\mathrm{MDM},f}$ on a tree T by bal_T or simply by bal when it is not necessary to specify the tree.

Lemma S1.15. *Let $n_1,\dots,n_k\in\mathbb{N}_{>0}$ and $n=n_1+\cdots+n_k$, and assume that $2\leqslant k\leqslant n-1$. Then*

$$e^{n_1}+\cdots+e^{n_k}+e^k\leqslant e^{n-k+1}+e^k+(k-1)e<e^n$$

Moreover, the first inequality is strict unless there is at most one exponent $n_i\geqslant 2$.

Proof. To begin with, notice that if $1\leqslant x\leqslant \min(a,b)$, then

$$e^a+e^b\leqslant e^{a+b-x}+e^x, \tag{1}$$

because

$$e^b-e^x=(e^{b-x}-1)e^x\leqslant(e^{b-x}-1)e^a=e^{a+b-x}-e^a.$$

Moreover, if $x<\min(a,b)$ then the inequality is clearly strict.

Now, applying $k-1$ times inequality (1) with $x=1$, we obtain

$$e^{n_1}+\cdots+e^{n_k}\leqslant e^{n_1+(n_2-1)+(n_3-1)+\cdots+(n_k-1)}+(k-1)e=e^{n-k+1}+(k-1)e,$$

which implies the first inequality. Moreover, this inequality is strict unless all n_i but, at most, one are 1, because (1) is strict if $x<a$ and $x<b$, which in this situation is translated to the existence of at least two n_i,n_j greater than 1.

As far as the second inequality goes, since $2\leqslant k\leqslant n-1$, and hence, in particular, $n\geqslant 3$ (and using, in the second inequality, that $x+1<e^x$ for every $x\in\mathbb{R}_{>0}$), we have

$$e^{n-k+1}+(k-1)e+e^k\leqslant 2e^{n-1}+(n-2)e<2e^{n-1}+e^{n-3}\cdot e=e^{n-2}(2e+1)<e^{n-2}\cdot e^2=e^n$$

as we claimed. □

Lemma S1.16. *Let $n_1,\dots,n_k,l\in\mathbb{N}$ be such that $k\geqslant 1$, $k+l\geqslant 2$, each $n_i\geqslant 2$, and let $n=n_1+\cdots+n_k$. Then*

$$e^{n_1}+\cdots+e^{n_k}+e^{k+l}<e^{n+l}$$

Proof. The case $l=0$ is a particular instance of the last lemma. So, we assume henceforth that $l\geqslant 1$. If $k=1$, so that $n=n_1$, then applying inequality (1) with $x=2$ we have

$$e^n+e^{l+1}\leqslant e^{n+l-1}+e^2$$

and the right hand side term is smaller than e^{n+l} because $n+l\geqslant 3$.

Assume finally that $k\geqslant 2$. Applying $k-1$ times inequality (1) with $x=2$, we obtain

$$e^{n_1}+\cdots+e^{n_k}+e^{k+l}\leqslant e^{n-2(k-1)}+(k-1)e^2+e^{k+l}$$

Now, since $2\leqslant k\leqslant n/2$, and hence $n\geqslant 4$,

$$\begin{aligned}e^{n-2(k-1)}+(k-1)e^2+e^{k+l}&\leqslant e^{n-2}+\left(\tfrac{n}{2}-1\right)e^2+e^{l+n/2}\\&<e^{n-2}+e^{n/2-2}\cdot e^2+e^{l+n/2}=e^{n-2}+e^{n/2}+e^{l+n/2}<3e^{n+l-2}<e^{n+l},\end{aligned}$$

as we claimed. □

Lemma S1.17. *The largest e^n-size of a tree in $\mathcal{T}_n^*$ is e^n+n, and it is reached exactly at the star FS_n.*

Proof. The cases $n = 1, 2$ are obvious, because $\mathcal{T}_n^*$ consists of a single tree. Let now $n \geqslant 3$. We shall prove that for every $T \in \mathcal{T}_n^* \setminus \{FS_n\}$, there is a tree $T' \in \mathcal{T}_n^*$ with $\delta(T') > \delta(T)$. This shows that no tree other than FS_n can have the maximum e^n-size.

So, let $T = T_1 \star \cdots \star T_m \in \mathcal{T}_n^* \setminus \{FS_n\}$, with $m \geqslant 2$. Let $l \geqslant 0$ be such that, for every $i = 1, \ldots, l$, the subtree T_i consists of a single node, and, for every $i = l+1, \ldots, m$, $T_i = T_{i,1} \star \cdots \star T_{i,n_i}$ with $n_i \geqslant 2$; cf. Fig. 6. Since $T \neq FS_n$, $l < m$. Let now T' be the tree

$$T' = T_1 \star \cdots \star T_l \star T_{l+1,1} \star \cdots \star T_{l+1,n_{l+1}} \star \cdots \star T_{m,1} \star \cdots \star T_{m,n_m}.$$

Then,

$$\delta(T) = e^m + l + \sum_{i=l+1}^{m} \Big(e^{n_i} + \sum_{j=1}^{n_i} \delta(T_{i,j})\Big) < e^{l+n_{l+1}+\cdots+n_m} + l + \sum_{i=l+1}^{m} \sum_{j=1}^{n_i} \delta(T_{i,j}) = \delta(T')$$

by Lemma S1.16. $\square$

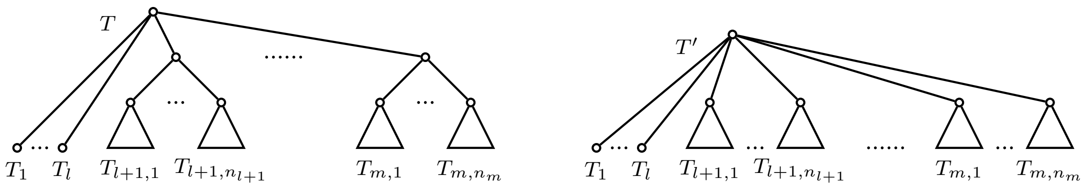

Figure 6: The trees T and T' in the proof of Lemma S1.17.

Lemma S1.18. *For every $T \in \mathcal{T}_n^*$ with $n \neq 1, 3$,*

$$2\mathfrak{C}(T) + \delta(T) \leqslant 2\mathfrak{C}(FS_n) + \delta(FS_n) = e^n + n,$$

and the inequality is strict if $T \neq FS_n$.

When $n = 1$, the maximum of $2\mathfrak{C} + \delta$ is $2\mathfrak{C}(FS_1) + \delta(FS_1) = 1$, and when $n = 3$, this maximum is $2\mathfrak{C}(K_3) + \delta(K_3) = 3e^2 + 4$ and it is reached exactly at K_3.

Proof. The cases $n = 1, 2$ are obvious, because then $\mathcal{T}_n^*$ consists of a single tree, and the cases $n = 3, 4, 5$ can be checked in the tables in supplementary file S2. Notice that $1 < e^1 + 1$ and $3e^2 + 4 < (e^3 + 3) + 4$; we shall use these two inequalities below. We prove now the general case $n \geqslant 6$ using the cases $n = 1, \ldots, 5$ and complete induction on n.

Let $T = T_1 \star \cdots \star T_k$, with $k \geqslant 2$ and $T_i \in \mathcal{T}_{n_i}^*$ for every $i = 1, \ldots, k$, so that $n = n_1 + \cdots + n_k \geqslant 6$. If $k = n$, then $n_i = 1$ for every i and $T = FS_n$, in which case $2\mathfrak{C}(T) + \delta(T) = e^n + n$. So, we shall assume that $k \leqslant n - 1$, and we shall prove that, in this case $2\mathfrak{C}(T) + \delta(T) < e^n + n$.

After renumbering the subtrees T_i if necessary, assume that there exists $l \geqslant 0$ such that $n_i = 3$ for every $i \leqslant l$ and $n_i \neq 3$ for every $i = l+1, \ldots, k$. We shall prove first of all that

$$\mathrm{MDM}(\delta(T_1), \ldots, \delta(T_k)) \leqslant \frac{1}{k} \sum_{i=1}^{k} (e^{n_i} + n_i - 2) \qquad (2)$$

Indeed, let $M = \mathit{Median}(\delta(T_1), \ldots, \delta(T_k))$. Then,

$$\mathrm{MDM}(\delta(T_1), \ldots, \delta(T_k)) = \frac{1}{k} \sum_{i=1}^{k} |\delta(T_i) - M| \leqslant \frac{1}{k} \sum_{i=1}^{k} |\delta(T_i) - 2| \leqslant \frac{1}{k} \sum_{i=1}^{k} (e^{n_i} + n_i - 2)$$

where the first inequality is due to the fact that M is the real number that minimizes the function $x \mapsto \sum_{i=1}^{k} |\delta(T_i) - x|$, and the second inequality holds because $|\delta(T_i) - 2| \leqslant e^{n_i} + n_i - 2$ for every $i = 1, \ldots, k$; on

its turn, this inequality is a consequence, when $n_i \geqslant 2$, of Lemma S1.17, and, when $n_i = 1$, of the fact that if $T_1 \in \mathcal{T}_1^*$, then $\delta(T_i) = 1$ and hence $|\delta(T_i) - 2| = 1 < e^1 + 1 - 2$. Now,

$$\begin{aligned}
2\mathfrak{C}(T) + \delta(T) &= 2\Big(\sum_{i=1}^{k} \mathfrak{C}(T_i) + \mathrm{MDM}(\delta(T_1), \ldots, \delta(T_k))\Big) + \sum_{i=1}^{k} \delta(T_i) + e^k \\
&= \sum_{i=1}^{k} (2\mathfrak{C}(T_i) + \delta(T_i)) + 2\mathrm{MDM}(\delta(T_1), \ldots, \delta(T_k)) + e^k \\
&= \sum_{i=1}^{l} (2\mathfrak{C}(T_i) + \delta(T_i)) + \sum_{i=l+1}^{k} (2\mathfrak{C}(T_i) + \delta(T_i)) + 2\mathrm{MDM}(\delta(T_1), \ldots, \delta(T_k)) + e^k \\
&\leqslant \sum_{i=1}^{l} (3e^2 + 4) + \sum_{i=l+1}^{k} (e^{n_i} + n_i) + \frac{2}{k} \sum_{i=1}^{k} (e^{n_i} + n_i - 2) + e^k \\
&\quad \text{(by the case } n = 3 \text{, the induction hypothesis, and inequality (2))} \\
&\leqslant \sum_{i=1}^{l} (e^3 + 3 + 4) + \sum_{i=l+1}^{k} (e^{n_i} + n_i) + \sum_{i=1}^{k} (e^{n_i} + n_i - 2) + e^k \\
&\quad \text{(because } k \geqslant 2 \text{ and } 3e^2 + 4 < e^3 + 3 + 4) \\
&= \sum_{i=1}^{k} (e^{n_i} + n_i) + 4l + \sum_{i=1}^{k} (e^{n_i} + n_i - 2) + e^k \\
&= 2\sum_{i=1}^{k} e^{n_i} + 2n + 4l - 2k + e^k \leqslant 2\sum_{i=1}^{k} e^{n_i} + e^k + 2n + 2k \\
&\leqslant 2e^{n-(k-1)} + e^k + 2(e+1)k + 2n - 2e \\
&\quad \text{(by the first inequality in Lemma S1.15)}
\end{aligned}$$

Thus, it remains to prove that, for every $n \geqslant 6$ and for every $2 \leqslant k \leqslant n - 1$,

$$2e^{n-(k-1)} + e^k + 2(e+1)k + 2n - 2e < e^n + n \tag{3}$$

Since, for every $n \geqslant 6$, the function

$$f_n(x) = e^n + n - \big(2e^{n-(x-1)} + e^x + 2(e+1)x + 2n - 2e\big) = e^n - 2e^{n-(x-1)} - e^x - 2(e+1)x - n + 2e$$

is concave, because $f_n''(x) < 0$, its minimum value on the closed interval $[2, n-1]$ is reached at one of its ends. So, in order to prove inequality (3) for every $n \geqslant 6$ and for every $k = 2, \ldots, n-1$, it is enough to prove that $f_n(2) > 0$ and $f_n(n-1) > 0$ for every $n \geqslant 6$. And, indeed

- $f_n(2) = e^n - 2e^{n-1} - n - e^2 - 2e - 4 > 0$ because the function $g(x) = e^x - 2e^{x-1} - x - e^2 - 2e - 4$ is increasing on $\mathbb{R}_{\geqslant 2}$ and $g(5) > 0$.
- $f_n(n-1) = e^n - e^{n-1} - (2e+3)n - 2e^2 + 4e + 2 > 0$ by a similar reason.

This finishes the proof of the statement. □

Now we can proceed with the proof of Theorem 19.(a) for $D = \mathrm{MDM}$. The cases $n = 2, 3, 4, 5$ can be checked in the tables in the supplementary file S2. Notice in particular that, when $n = 4$, the maximum is

$$\mathfrak{C}(K_4) = \frac{3}{2}(e^2 + 1) < \frac{1}{2}(e^3 + 2) + 2;$$

we shall use this inequality below. We prove now, using the cases $n = 1, \ldots, 5$ and complete induction on n, that, for every $n \geqslant 6$,

The tree in $\mathcal{T}_n^*$ with maximum $\mathfrak{C}$ is $FS_1 \star FS_{n-1}$, with $\mathfrak{C}(FS_1 \star FS_{n-1}) = \frac{1}{2}(e^{n-1} + n - 2)$

Recall that, as in the previous sections, Lemma S1.3 implies that the maximum $\mathfrak{C}$ value on $\mathcal{T}_n^*$ is reached at a tree with binary root. So, let $T = T_1 \star T_2 \in \mathcal{T}_n^*$, with $T_1 \in \mathcal{T}_{n_1}^*$ and $T_2 \in \mathcal{T}_{n_2}^*$. We must distinguish two cases:

a) Assume that $n_1 = 1$, and therefore $n_2 = n - 1 \geqslant 5$. In this case,

$$\mathfrak{C}(T) = \mathfrak{C}(T_2) + \frac{1}{2}(\delta(T_2) - 1) = \frac{1}{2}(2\mathfrak{C}(T_2) + \delta(T_2) - 1) \leqslant \frac{1}{2}(e^{n_2} + n_2 - 1) = \frac{1}{2}(e^{n-1} + n - 2)$$

by Lemma S1.18. Moreover, the equality holds only when $T_2 = FS_{n-1}$.

b) Assume that $n_1, n_2 \geqslant 2$ and, without any loss of generality, that $\delta(T_2) \leqslant \delta(T_1)$. Then,

$$\begin{aligned}\mathfrak{C}(T) &= \mathfrak{C}(T_1) + \mathfrak{C}(T_2) + \frac{1}{2}\big(\delta(T_1) - \delta(T_2)\big) \\ &< \frac{1}{2}(e^{n_1-1} + n_1 - 2) + 2 + \frac{1}{2}(e^{n_2-1} + n_2 - 2) + 2 + \frac{1}{2}(e^{n_1} + n_1 - n_2) = (*)\end{aligned}$$

This inequality is due to the following facts. On the one hand, $n_2 \leqslant \delta(T_2)$ and $\delta(T_1) \leqslant e^{n_1} + n_1$, by Lemma S1.17, and hence $\delta(T_1) - \delta(T_2) \leqslant e^{n_1} + n_1 - n_2$. On the other hand, by the induction hypothesis, $\mathfrak{C}(T_i) \leqslant \frac{1}{2}(e^{n_i-1} + n_i - 2) < \frac{1}{2}(e^{n_i-1} + n_i - 2) + 2$, unless $n_i = 4$, in which case we still have $\mathfrak{C}(T_i) \leqslant \mathfrak{C}(K_4) < \frac{1}{2}(e^{n_i-1} + n_i - 2) + 2$.

Let us continue

$$(*) = \frac{1}{2}((1 + e)e^{n_1-1} + e^{n_2-1} + 2n_1 + 4) \leqslant \frac{1}{2}((2 + e)e^{n-3} + 2n)$$

because $n_1, n_2 \leqslant n - 2$. So, it remains to prove that, for every $n \geqslant 6$,

$$(2 + e)e^{n-3} + 2n < e^{n-1} + n - 2$$

This is equivalent to

$$(e^2 - e - 2)e^{n-3} - n - 2 > 0,$$

which is easy to prove, for instance noticing that $f(x) = (e^2 - e - 2)e^{x-3} - x - 2$ is increasing on $\mathbb{R}_{\geqslant 3}$ and that $f(5) > 0$. This finishes the proof of Theorem 19 for $D = \text{MDM}$.

E Proof of the thesis of Theorem 19 for $\mathfrak{C}_{sd,e^n}$

The proof of this case follows closely that of the case when $D = \text{MDM}$ given in the last section. To begin with, it turns out that a key lemma similar to Lemma S1.18 also holds when $D = sd$. To simplify the notations, we shall denote in this section δ_{e^n} and $\mathfrak{C}_{sd,e^n}$ by δ and $\mathfrak{C}$, respectively, and we shall denote $bal_{sd,f}$ on a tree T by bal_T or simply by bal when it is not necessary to specify the tree.

Lemma S1.19. *For every $T \in \mathcal{T}_n^*$ with $n \neq 1, 3$,*

$$\sqrt{2} \cdot \mathfrak{C}(T) + \delta(T) \leqslant \sqrt{2} \cdot \mathfrak{C}(FS_n) + \delta(FS_n) = e^n + n.$$

and the inequality is strict if $T \neq FS_n$.

When $n = 1$, the maximum of $\sqrt{2} \cdot \mathfrak{C} + \delta$ is $\sqrt{2} \cdot \mathfrak{C}(FS_1) + \delta(FS_1) = 1 + e$, and when $n = 3$, this maximum is $\sqrt{2} \cdot \mathfrak{C}(K_3) + \delta(K_3) = 3e^2 + 4$.

Proof. The cases $n = 1, 2$ are obvious, and the cases $n = 3, 4, 5$ can be checked in Table 2 in the supplementary file S2. We shall use that $1 < e^1 + 1$ and the following inequalities:

$$\sqrt{2} \cdot \mathfrak{C}(K_3) + \delta(K_3) = 3e^2 + 4 < (e^3 + 3) + 4 \quad (4a)$$

$$\delta(K_3) = 2e^2 + 3 < (e^3 + 3) - 5 \quad (4b)$$

We prove the general case $n \geqslant 6$ by induction on n using an argument very similar to the one given in the proof of Lemma S1.18. Let $T = T_1 \star \cdots \star T_k$, with $k \geqslant 2$ and $T_i \in \mathcal{T}_{n_i}^*$ for every $i = 1, \ldots, k$, so that

$n = n_1 + \cdots + n_k$. If $k = n$, then $n_i = 1$ for every i and $T = FS_n$, in which case $\sqrt{2}\cdot\mathfrak{C}(T) + \delta(T) = e^n + n$. So, we shall assume that $k \leqslant n-1$. Without any loss of generality, we assume that there exists $l \geqslant 0$ such that $T_i = K_3$ if, and only if, $i \leqslant l$.

Now, it turns out that

$$sd(\delta(T_1), \dots, \delta(T_k)) \leqslant \frac{1}{\sqrt{k-1}}\Big(\sum_{i=1}^{k}(e^{n_i} + n_i - 2) - 5l\Big) \tag{5}$$

Indeed, let $m = (\delta(T_1) + \ldots + \delta(T_k))/k$. Then,

$$\operatorname{var}(\delta(T_1), \dots, \delta(T_k)) = \frac{1}{k-1}\sum_{i=1}^{k}(\delta(T_i) - m)^2 \leqslant \frac{1}{k-1}\sum_{i=1}^{k}(\delta(T_i) - 2)^2$$

because m is the real number that minimizes the function $x \mapsto \sum\limits_{i=1}^{k}(\delta(T_i) - x)^2$. Taking square roots,

$$\begin{aligned} sd(\delta(T_1), \dots, \delta(T_k)) &\leqslant \sqrt{\frac{1}{k-1}\sum_{i=1}^{k}(\delta(T_i) - 2)^2} \leqslant \frac{1}{\sqrt{k-1}}\sum_{i=1}^{k}|\delta(T_i) - 2| \\ &\leqslant \frac{1}{\sqrt{k-1}}\Big(\sum_{i=1}^{k}(e^{n_i} + n_i - 2) - 5l\Big) \end{aligned}$$

where the second last inequality is a consequence of Lemma S1.17, inequality (4b), and the fact, already used in the previous section, that if $T_1 \in \mathcal{T}_1^*$, then $|\delta(T_1) - 2| = e - 1 = e + 1 - 2$. Then

$$\begin{aligned} \sqrt{2}\mathfrak{C}(T) + \delta(T) &= \sqrt{2}\Big(\sum_{i=1}^{k}\mathfrak{C}(T_i) + sd(\delta(T_1), \dots, \delta(T_k))\Big) + \sum_{i=1}^{k}\delta(T_i) + e^k \\ &= \sum_{i=1}^{k}(\sqrt{2}\mathfrak{C}(T_i) + \delta(T_i)) + \sqrt{2}sd(\delta(T_1), \dots, \delta(T_k)) + e^k \\ &\leqslant \sum_{i=1}^{k}(e^{n_i} + n_i) + 4l + \frac{\sqrt{2}}{\sqrt{k-1}}\Big(\sum_{i=1}^{k}(e^{n_i} + n_i - 2) - 5l\Big) + e^k \\ &\quad \text{(by the induction hypothesis and inequalities (4a) and (5))} \\ &\leqslant \sum_{i=1}^{k}e^{n_i} + n + 4l + \sqrt{2}\Big(\sum_{i=1}^{k}e^{n_i} + n - 2k - 5l\Big) + e^k \\ &\leqslant (1+\sqrt{2})\sum_{i=1}^{k}e^{n_i} + (1+\sqrt{2})n - 2\sqrt{2}k + e^k \\ &\leqslant (1+\sqrt{2})e^{n-(k-1)} + (1+\sqrt{2})(k-1)e + e^k + (1+\sqrt{2})n - 2\sqrt{2}k \\ &\quad \text{(because of the first inequality in Lemma S1.15)} \\ &= (1+\sqrt{2})e^{n-(k-1)} + (1+\sqrt{2})n + e^k + (e + \sqrt{2}e - 2\sqrt{2})k - (1+\sqrt{2})e \end{aligned}$$

Thus, it remains to prove that, for every $n \geqslant 6$ and for every $2 \leqslant k \leqslant n-1$,

$$(1+\sqrt{2})e^{n-(k-1)} + (1+\sqrt{2})n + e^k + (e + \sqrt{2}e - 2\sqrt{2})k - (1+\sqrt{2})e < e^n + n \tag{6}$$

Now, for every $n \geqslant 1$, the function

$$\begin{aligned} f_n(x) &= e^n + n - \big((1+\sqrt{2})e^{n-(x-1)} + e^x + (1+\sqrt{2})n + (e + \sqrt{2}e - 2\sqrt{2})x - (1+\sqrt{2})e\big) \\ &= e^n - (1+\sqrt{2})e^{n-(x-1)} - e^x - \sqrt{2}n - (e + \sqrt{2}e - 2\sqrt{2})x + (1+\sqrt{2})e \end{aligned}$$

is concave, and therefore the minimum value of $f_n(x)$ on the closed interval $[2, n-1]$ will be reached at one of its ends. So, in order to prove inequality (6) for every $n \geqslant 6$ and every $k = 2, \dots, n-1$, it is enough to prove that $f_n(2) > 0$ and $f_n(n-1) > 0$ for every $n \geqslant 6$. And, indeed

- $f_n(2) = e^n - (1+\sqrt{2})e^{n-1} - \sqrt{2}n - (e^2 + \sqrt{2}e + e - 4\sqrt{2}) > 0$ because the function $g(x) = e^x - (1+\sqrt{2})e^{x-1} - \sqrt{2}x - (e^2+\sqrt{2}e+e-4\sqrt{2})$ is increasing on $\mathbb{R}_{\geqslant 3}$ and $g(5) > 0$.
- $f_n(n-1) = e^n - (1+\sqrt{2})e^2 - e^{n-1} - \sqrt{2}n - (e+\sqrt{2}e-2\sqrt{2})(n-1) + (1+\sqrt{2})e$ by a similar reason.

This finishes the proof of the lemma. □

From here on, the proof of Theorem 19 for $D = sd$ proceeds as the one for $D = \mathrm{MDM}$ given in the previous section, using Lemma S1.19 instead of Lemma S1.18; to ease the task of the reader we provide it. The cases $n = 2, 3, 4, 5$ can be checked in Table 2 in the supplementary file S2. Notice in particular that, when $n = 4$, the maximum $\mathfrak{C}$ value is

$$\mathfrak{C}(K_4) = \frac{3}{\sqrt{2}}(e^2+1) < \frac{1}{\sqrt{2}}(e^3+2) + 2.5 \tag{7}$$

we shall use it below.

We prove now, using the cases $n = 1, \ldots, 5$ and complete induction on n, that, for every $n \geqslant 6$,

The tree in $\mathcal{T}_n^*$ with maximum $\mathfrak{C}$ is $FS_1 \star FS_{n-1}$, with $\mathfrak{C}(FS_1 \star FS_{n-1}) = \frac{1}{\sqrt{2}}(e^{n-1}+n-2)$

To begin with, notice that Lemma S1.8 implies that the maximum $\mathfrak{C}$ value on $\mathcal{T}_n^*$ is reached at a tree with binary root. So, let $T = T_1 \star T_2 \in \mathcal{T}_n^*$, with $T_1 \in \mathcal{T}_{n_1}^*$ and $T_2 \in \mathcal{T}_{n_2}^*$. We must distinguish two cases:

a) Assume that $n_1 = 1$, and therefore $n_2 = n-1 \geqslant 5$. In this case,

$$\mathfrak{C}(T) = \mathfrak{C}(T_2) + \frac{1}{\sqrt{2}}(\delta(T_2)-1) = \frac{1}{\sqrt{2}}(\sqrt{2}\mathfrak{C}(T_2) + \delta(T_2) - 1) \leqslant \frac{1}{\sqrt{2}}(e^{n-1}+n-1-1) = \frac{1}{\sqrt{2}}(e^{n-1}+n-2)$$

by Lemma S1.18. Moreover, the equality holds only when $T_2 = FS_{n-1}$.

b) Assume that $n_1, n_2 \geqslant 2$ and, without any loss of generality, that $\delta(T_2) \leqslant \delta(T_1)$. Then,

$$\begin{aligned}\mathfrak{C}(T) &= \mathfrak{C}(T_1) + \mathfrak{C}(T_2) + \frac{1}{\sqrt{2}}\big(\delta(T_1) - \delta(T_2)\big)\\ &< \frac{1}{\sqrt{2}}(e^{n_1-1}+n_1-2) + 2.5 + \frac{1}{\sqrt{2}}(e^{n_2-1}+n_2-2) + 2.5 + \frac{1}{\sqrt{2}}(e^{n_1}+n_1-n_2) = (*)\end{aligned}$$

This inequality is due to the following facts. On the one hand, $n_2 \leqslant \delta(T_2)$ and $\delta(T_1) \leqslant e^{n_1} + n_1$, by Lemma S1.17, and hence $\delta(T_1) - \delta(T_2) \leqslant e^{n_1} + n_1 - n_2$. On the other hand, by the induction hypothesis, $\mathfrak{C}(T_i) \leqslant \frac{1}{\sqrt{2}}(e^{n_i-1}+n_i-2) < \frac{1}{\sqrt{2}}(e^{n_i-1}+n_i-2) + 2.5$, unless $n_i = 4$, in which case we still have $\mathfrak{C}(T_i) \leqslant \mathfrak{C}(K_4) < \frac{1}{\sqrt{2}}(e^{n_i-1}+n_i-2) + 2.5$ by inequality (7).

Let us continue

$$\begin{aligned}(*) &= \frac{1}{\sqrt{2}}((1+e)e^{n_1-1} + e^{n_2-1} + 2n_1 - 4) + 5 \leqslant \frac{1}{\sqrt{2}}((2+e)e^{n-3} + 2n + 5\sqrt{2} - 8)\\ &\quad \text{(because } n_1, n_2 \leqslant n-2\text{)}\\ &< \frac{1}{\sqrt{2}}((2+e)e^{n-3} + 2n) < \frac{1}{\sqrt{2}}(e^{n-1}+n-2)\end{aligned}$$

because $(2+e)e^{n-3} + 2n < e^{n-1} + n - 2$, as it was proven in the last step of the proof of Theorem 19 for $D = \mathrm{MDM}$ in the last section. This finishes the proof of Theorem 19 for $D = sd$.

F Proof of the thesis of Theorem 19 for $\mathfrak{C}_{\mathrm{var},e^n}$

The stated maximum value of $\mathfrak{C}_{\mathrm{var},e^n}$ on $\mathcal{T}_n^*$, for $n = 2, \ldots, 5$, can be checked in Table 2 in the supplementary file S2. As far as the case when $n \geqslant 6$, it is a direct consequence of the corresponding result for $D = sd$, established in the previous section.

Indeed, to begin with, notice that, since, for every node v in a tree T, $bal_{\mathrm{var},f}(v) = bal_{sd,f}(v)^2$, we have that, for every tree T,

$$\mathfrak{C}_{\mathrm{var},f}(T) = \sum_{v\in V_{int}(T)} bal_{sd,f}(v)^2 \leqslant \Big(\sum_{v\in V_{int}(T)} bal_{sd,f}(v)\Big)^2 = \mathfrak{C}_{sd,f}(T)^2$$

So, for every $T \in \mathcal{T}_n^*$ with $n \geqslant 6$,

$$\begin{aligned}\mathfrak{C}_{\mathrm{var},e^n}(T) &\leqslant \mathfrak{C}_{sd,e^n}(T)^2 \leqslant \mathfrak{C}_{sd,e^n}(FS_1 \star FS_{n-1})^2 = \tfrac{1}{2}(e^{n-1}+n-2)^2\\ &= \mathfrak{C}_{\mathrm{var},e^n}(FS_1 \star FS_{n-1})\end{aligned}$$

where the second inequality is strict if $T \neq FS_1 \star FS_{n-1}$.
