# Supplementary material for: Sound Colless-like balance indices for multifurcating trees
Source: PLoS One. 2018 Sep 25;13(9):e0203401. doi: 10.1371/journal.pone.0203401 (PMC6155497; doi:10.1371/journal.pone.0203401)
Supplement: S2 File — The file provides two tables, quoted in the main text, with the values of several Colless-like indices on Tn* for n = 2, 3, 4, 5. (PDF) [file pone.0203401.s002.pdf]

Sound Colless-like balance indices for multifurcating trees. Supplementary file S2: Some tables

Arnau Mir, Francesc Rosselló, Lucía Rotger

Tree	δ_f	$\mathfrak{C}_{\mathrm{MDM},f}$	$\mathfrak{C}_{\mathrm{var},f}$	$\mathfrak{C}_{sd,f}$
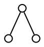	$x_2 + 2x_0$	0	0	0
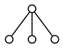	$x_3 + 3x_0$	0	0	0
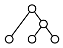	$2x_2 + 3x_0$	$\frac{1}{2}(x_2 + x_0)$	$\frac{1}{2}(x_2 + x_0)^2$	$\frac{1}{\sqrt{2}}(x_2 + x_0)$
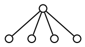	$x_4 + 4x_0$	0	0	0
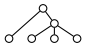	$x_3 + x_2 + 4x_0$	$\frac{1}{2}(x_3 + 2x_0)$	$\frac{1}{2}(x_3 + 2x_0)^2$	$\frac{1}{\sqrt{2}}(x_3 + 2x_0)$
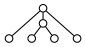	$x_3 + x_2 + 4x_0$	$\frac{1}{3}(x_2 + x_0)$	$\frac{1}{3}(x_2 + x_0)^2$	$\frac{1}{\sqrt{3}}(x_2 + x_0)$
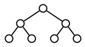	$3x_2 + 4x_0$	0	0	0
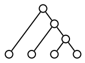	$3x_2 + 4x_0$	$\frac{3}{2}(x_2 + x_0)$	$\frac{5}{2}(x_2 + x_0)^2$	$\frac{3}{\sqrt{2}}(x_2 + x_0)$
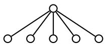	$x_5 + 5x_0$	0	0	0
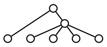	$x_4 + x_2 + 5x_0$	$\frac{1}{2}(x_4 + 3x_0)$	$\frac{1}{2}(x_4 + 3x_0)^2$	$\frac{1}{\sqrt{2}}(x_4 + 3x_0)$
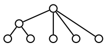	$x_4 + x_2 + 5x_0$	$\frac{1}{4}(x_2 + x_0)$	$\frac{1}{4}(x_2 + x_0)^2$	$\frac{1}{2}(x_2 + x_0)$
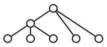	$2x_3 + 5x_0$	$\frac{1}{3}(x_3 + 2x_0)$	$\frac{1}{3}(x_3 + 2x_0)^2$	$\frac{1}{\sqrt{3}}(x_3 + 2x_0)$
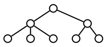	$x_3 + 2x_2 + 5x_0$	$\frac{1}{2}\lvert x_3 - x_2 + x_0\rvert$	$\frac{1}{2}(x_3 - x_2 + x_0)^2$	$\frac{1}{\sqrt{2}}\lvert x_3 - x_2 + x_0\rvert$
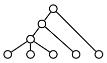	$x_3 + 2x_2 + 5x_0$	$\frac{1}{2}(2x_3 + x_2 + 5x_0)$	$\frac{1}{2}(x_3 + 2x_0)^2 +$ $\frac{1}{2}(x_3 + x_2 + 3x_0)^2$	$\frac{1}{\sqrt{2}}(2x_3 + x_2 + 5x_0)$
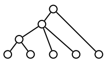	$x_3 + 2x_2 + 5x_0$	$\frac{1}{6}(3x_3 + 5x_2 + 11x_0)$	$\frac{1}{3}(x_2 + x_0)^2 +$ $\frac{1}{2}(x_3 + x_2 + 3x_0)^2$	$\frac{1}{\sqrt{3}}(x_2 + x_0) +$ $\frac{1}{\sqrt{2}}(x_3 + x_2 + 3x_0)$
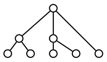	$x_3 + 2x_2 + 5x_0$	$\frac{1}{3}(x_2 + x_0)$	$\frac{1}{3}(x_2 + x_0)^2$	$\frac{1}{\sqrt{3}}(x_2 + x_0)$
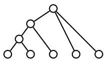	$x_3 + 2x_2 + 5x_0$	$\frac{7}{6}(x_2 + x_0)$	$\frac{11}{6}(x_2 + x_0)^2$	$\frac{\sqrt{3}+2\sqrt{2}}{\sqrt{6}}(x_2 + x_0)$
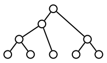	$4x_2 + 5x_0$	$x_2 + x_0$	$(x_2 + x_0)^2$	$\sqrt{2}(x_2 + x_0)$
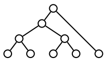	$4x_2 + 5x_0$	$\frac{3}{2}(x_2 + x_0)$	$\frac{9}{2}(x_2 + x_0)^2$	$\frac{3}{\sqrt{2}}(x_2 + x_0)$
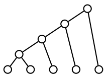	$4x_2 + 5x_0$	$3(x_2 + x_0)$	$7(x_2 + x_0)^2$	$3\sqrt{2}(x_2 + x_0)$

Table A: Abstract values of δ_f, $\mathfrak{C}_{\mathrm{MDM},f}$, $\mathfrak{C}_{\mathrm{var},f}$, and $\mathfrak{C}_{sd,f}$ on $\mathcal{T}^*_n$ for $n = 2, 3, 4, 5$. We denote $f(i)$ by x_i.

Tree	$\delta_{\ln}$	$\mathfrak{C}_{\mathrm{M},\ln}$	Pos.	$\mathfrak{C}_{\mathrm{var},\ln}$	Pos.	$\mathfrak{C}_{sd,\ln}$	Pos.	δ_{e^n}	$\mathfrak{C}_{\mathrm{M},e^n}$	Pos.	$\mathfrak{C}_{\mathrm{var},e^n}$	Pos.	$\mathfrak{C}_{sd,e^n}$	Pos.
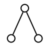	3.5514	0	(1)	0	(1)	0	(1)	9.3891	0	(1)	0	(1)	0	(1)
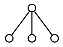	4.7437	0	(1)	0	(1)	0	(1)	23.0855	0	(1)	0	(1)	0	(1)
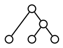	6.1029	1.2757	(2)	3.2549	(2)	1.8041	(2)	17.7781	4.1945	(2)	35.1881	(2)	5.9320	(2)
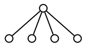	5.9048	0	(1)	0	(1)	0	(1)	58.5982	0	(1)	0	(1)	0	(1)
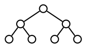	8.6543	0	(1)	0	(1)	0	(1)	26.1672	0	(1)	0	(1)	0	(1)
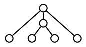	7.2951	0.8505	(2)	2.1700	(2)	1.4731	(2)	31.4746	2.7964	(2)	23.4588	(2)	4.8434	(2)
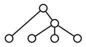	7.2951	1.8718	(3)	7.0075	(3)	2.6472	(3)	31.4746	11.0428	(3)	243.8855	(4)	15.6168	(3)
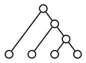	8.6543	3.8272	(4)	16.2747	(4)	5.4124	(4)	26.1672	12.5836	(4)	175.9407	(3)	17.7959	(4)
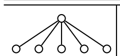	7.0436	0	(1)	0	(1)	0	(1)	153.4132	0	(1)	0	(1)	0	(1)
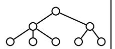	9.8466	0.5961	(2)	0.7107	(2)	0.8430	(2)	39.8636	6.8482	(4)	93.7968	(5)	9.6849	(4)
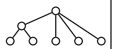	8.4563	0.6379	(3)	1.6275	(3)	1.2757	(3)	66.9872	2.0973	(2)	17.5941	(2)	4.1945	(2)
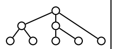	9.8466	0.8505	(4)	2.1700	(4)	1.4731	(4)	39.8636	2.7964	(3)	23.4588	(3)	4.8434	(3)
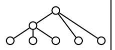	8.4873	1.2479	(5)	4.6717	(5)	2.1614	(5)	45.1711	7.3618	(5)	162.5903	(7)	12.7511	(6)
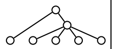	8.4563	2.4524	(6)	12.0287	(7)	3.4682	(6)	66.9872	28.7991	(12)	1658.7734	(12)	40.7280	(12)
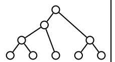	11.2058	2.5514	(7)	6.5099	(6)	3.6083	(7)	34.5562	8.3891	(6)	70.3763	(4)	11.8639	(5)
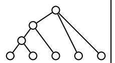	9.8466	2.9767	(8)	11.9348	(8)	4.7503	(8)	39.8636	9.7872	(7)	129.0231	(6)	15.6188	(7)
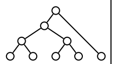	11.2058	3.8272	(9)	29.2944	(11)	5.4124	(9)	34.5562	12.5836	(8)	316.6932	(8)	17.7959	(8)
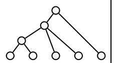	9.8466	3.9980	(10)	21.9842	(9)	5.9244	(10)	39.8636	18.0336	(9)	487.8092	(9)	26.3922	(9)
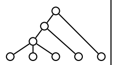	9.8466	5.0194	(11)	26.8218	(10)	7.0985	(11)	39.8636	26.2801	(11)	708.2359	(11)	37.1656	(11)
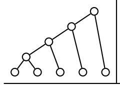	11.2058	7.6543	(12)	45.5691	(12)	10.8249	(12)	34.5562	25.1672	(10)	492.6338	(10)	35.5918	(10)

Table B: Numerical values (rounded to 4 decimal places) of δ_f (for $f(n) = \ln(n+e)$ and $f(n) = e^n$) and of $\mathfrak{C}_{D,f}$ (for every combination of D = MDM, var or sd and $f(n) = \ln(n+e)$ or $f(n) = e^n$) on $\mathcal{T}_n^*$, for every $n = 2, 3, 4, 5$. To win some horizontal space, in the subscripts we have replaced MDM by simply M, and we have denoted $\ln(n+e)$ by ln. The columns labelled "Pos." give the position of the tree in its $\mathcal{T}_n^*$ in increasing order of the Colless-like balance index corresponding to the column on its left. The rows are sorted, for each n, in increasing order of $\mathfrak{C}_{\mathrm{MDM},\ln}$.
